# Structural Characterization and Functional Annotation of Hypothetical Proteins in the Multidrug‐Resistant Strains of *Pseudomonas aeruginosa*

**DOI:** 10.1155/bmri/2974616

**Published:** 2026-02-02

**Authors:** Fatemeh Nasiri Khoonsari, Zahra Zafari

**Affiliations:** ^1^ Department of Biology, Shahed University, Tehran, Iran, shahed.ac.ir

**Keywords:** drug resistance, hypothetical proteins, *Pseudomonas aeruginosa*, vaccine candidate

## Abstract

**Introduction:**

*Pseudomonas aeruginosa* is a leading cause of severe nosocomial infections worldwide. This opportunistic pathogen is associated with increased morbidity and mortality rates due to its high levels of antibiotic resistance. The identification of novel therapeutic targets is therefore a pressing global health priority. This study aims to identify potential drug targets and vaccine candidates through structural characterization and functional annotation of hypothetical proteins (HPs) commonly found in multidrug‐resistant *Pseudomonas aeruginosa* strains.

**Methods:**

The multidrug‐resistant and carbapenem‐resistant strains of *Pseudomonas aeruginosa* were retrieved from genomic databases and 15 common HPs among these resistant strains, with a minimum length of 200 amino acids, were obtained and bioinformatics tools were employed to predict the structural, functional, and immunological properties of these common HPs.

**Results:**

Two common HPs (gene ID: 2877781443 and 2877781545) were identified as the most promising drug and vaccine candidates among the investigated HPs based on their structural and physicochemical properties, functional domains, signals peptides, subcellular localization, pathogenicity factors, toxicity, antigenicity, and allergenicity.

**Conclusion:**

The findings of this study will contribute to the development of novel vaccine and drug candidates against *Pseudomonas aeruginosa* through experimental validations.

## 1. Introduction

*Pseudomonas aeruginosa* (*P. aeruginosa*) is a leading cause of severe nosocomial infections ranging from acute to life‐threatening chronic conditions. The mortality rate associated with these infections is notably high, particularly in immunocompromised individuals or those with chronic diseases such as cystic fibrosis, cancer, HIV/AIDS, bronchiectasis, chronic wounds, or chronic urinary tract infections [1–3]. *P. aeruginosa* is renowned for its intrinsic and acquired antibiotic resistance. The growing number of multidrug‐resistant (MDR) and extensively drug‐resistant (XDR) strains of *P. aeruginosa* has significantly complicated treatment options, necessitating the urgent development of novel therapeutic approaches [4]. The carbapenem group of antibiotics including Meropenem, Doripenem, Ertapanem, and Imipenem are currently the last line of defense against these infections, but their effectiveness is waning due to increasing resistance. Furthermore, *P. aeruginosa* is a gram‐negative, aerobic, and facultative anaerobic bacillus, exhibiting remarkable adaptability and survival capabilities in diverse environments and ecological niches. The high variability of this microorganism may represent the main obstacle to the development of an effective vaccine and the absence of a *P. aeruginosa* licensed vaccine exacerbates the problem [5, 6]. Therefore, the World Health Organization (WHO) has prioritized the development of new treatment approaches for carbapenem‐resistant *P. aeruginosa* [3, 7–9].

*P. aeruginosa* possesses a large genome, ranging from 5.5 to 7 megabases, which endows it with a higher degree of complexity compared to other bacterial species [10, 11]. The highly adaptable genome of *P. aeruginosa* broadly comprises three categories of genes: (a) genes with established functional characteristics, (b) conserved hypothetical genes shared across multiple organisms, and (c) genes encoding hypothetical proteins (HPs) of unknown function. Proteomic studies of *P. aeruginosa* have revealed that approximately 25% of its proteins are classified as HPs. Many of these HPs are implicated in human diseases, highlighting their significance. Despite their unknown specific functions, the investigation of these HPs can provide valuable insights into the bacterium’s biochemical and physiological pathways. Such research can uncover new structures, functions, domains, motifs, and markers, ultimately helping to the discovery of novel strategies for early detection or treatment of infectious diseases, and new vaccine and drug candidates [12, 13]. While HPs in *P. aeruginosa* have been functionally annotated in the previous studies, the structural characterization and functional annotation of common HPs among resistant strains remain unexplored. Furthermore, the focus of the existing reports has been typically limited to a single HP or a single strain. In this study, we consider common HPs across to MDR, XDR and carbapenem‐resistant strains of *P. aeruginosa* and performed structural, functional, and immunoinformatic analysis with the power of different databases and bioinformatics tools to identify potential drug and vaccine candidates against the resistant strains of *P. aeruginosa*.

## 2. Materials and Methods

### 2.1. Study Design and Sequence Retrieval

**Figure 1:**
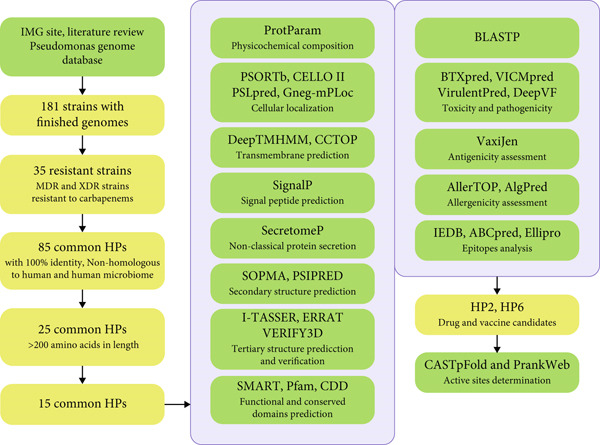
A schematic workflow for in silico analysis of common hypothetical proteins (HPs) associated with resistant strains of *P. aeruginosa.* Structural, functional, and immunological prediction with different bioinformatic tools (green rectangles) were performed for 15 common HPs and ultimately, two common HPs were identified as drug and vaccine candidates.

All *P. aeruginosa* strains with finished genomes were obtained from the Integrated Microbial Genome and Microbiomes (IMG/M) database (https://img.jgi.doe.gov/), a comprehensive resource for the analysis of microbial genomes and metagenomes [14]. The strains exhibiting MDR and XDR phenotypes were identified through literature review and the Pseudomonas Genome database (https://www.pseudomonas.com/) [15]. This selection process focused on strains resistant to carbapenem antibiotics, including Imipenem, Doripenem, Meropenem, and Ertapenem. Common HPs among these highly resistant strains were identified through IMG/M database as mentioned in our previously published manuscript [3]. Subsequently, 15 common HPs with the length of 200 amino acids or more, with 100% identity and nonhomologous to human and human microbiome were selected and their sequences were retrieved from IMG/M for further detailed analysis with online bioinformatic servers and databases (Figure 1) [15, 16].

### 2.2. Primary Structural Analysis of HPs

The various physical and chemical parameters of the selected common HPs were predicted by ProtParam (https://web.expasy.org/protparam/) [17, 18]. Subcellular localization of HPs was predicted through the utilization of four bioinformatic servers: PSORTb 3.0.3 (https://www.psort.org/psortb/), CELLO II 2.5 (http://cello.life.nctu.edu.tw/), PSLpred (http://webs.iiitd.edu.in/raghava/pslpred/), and Gneg‐mPLoc (http://www.csbio.sjtu.edu.cn/bioinf/Gneg-multi/) [19–22]. DeepTMHMM 1.0 (https://services.healthtech.dtu.dk/services/DeepTMHMM-1.0/) and The Consensus Constrained TOPology prediction (CCTOP) (https://cctop.ttk.hu/job/submit) were employed to investigate the presence or absence of transmembrane helices. Transmembrane helices are critical structural elements in membrane proteins [23, 24]. DeepTMHMM is a deep learning model‐based algorithm, offering a reliable prediction with high accuracy. CCTOP is a more advanced method, leveraging structural information from PDBTM, TOPDB, and TOPDOM databases, in conjunction with 10 additional advanced prediction algorithms [23]. The SignalP 6.0 database (https://services.healthtech.dtu.dk/services/SignalP-6.0/) was utilized to predict the presence of signal peptides and their probable cleavage site in HPs [25, 26]. Signal peptides are short peptides responsible for the secretion of proteins across biological membranes. However, some bacterial proteins employ nonclassical secretion pathways, bypassing the requirement for a signal peptide. The SecretomeP 2.0 database (https://services.healthtech.dtu.dk/services/SecretomeP-2.0/) was used to predict the nonclassical protein secretion pathway of 15 HPs. This server integrates various post‐translational modification (PTM) data along with protein localization information to enhance its predictive accuracy [27].

### 2.3. Secondary Structural Analysis of HPs

To predict the secondary structure of the HPs, we employed the SOPMA (https://npsa-prabi.ibcp.fr/cgi-bin/npsa_automat.pl?page=/NPSA/npsa_sopma_f.html) and PSIPRED database (http://bioinf.cs.ucl.ac.uk/psipred/) [28, 29]. SOPMA, a widely utilized tool for the secondary structure prediction of proteins, achieves an accuracy of approximately 69.5% in predicting the three‐state secondary structure, which includes alpha helices, beta sheets, and coils [28, 30].

### 2.4. Tertiary Structural Analysis of HPs

A protein’s biological function is primarily determined by its tertiary structure. In this study, the tertiary structure of HPs was predicted by the I‐TASSER (https://zhanggroup.org/I-TASSER/). This server employs a hierarchical approach to construct 3D atomic models of proteins, utilizing their amino acid sequences as input [31, 32]. The quality of these predicted structures was validated by ERRAT and VERIFY 3D tools available on the SAVES 6.1 database (https://saves.mbi.ucla.edu/). A higher ERRAT score generally corresponds to a more reliable structure, making it a crucial resource for validating computational protein models and experimental structures [33].

### 2.5. Functional Annotation of HPs

Understanding the domain architecture of proteins is a cornerstone of functional characterization [34]. This study employed the SMART 10 (https://smart.embl.de/) and Pfam 38.0 (http://pfam.xfam.org/) databases to predict the functional domains of HPs [35, 36]. SMART is a comprehensive web resource dedicated to the identification and annotation of protein domains, as well as the analysis of protein domain architectures. The latest version, includes over 1300 manually curated models for various protein domains and synchronizes its underlying protein databases with major resources like UniProt and STRING [36, 37]. On the other hand, Pfam, which is now hosted by InterPro, is a widely utilized database that catalogs protein families and domains, providing essential annotations for genomic sequences. By classifying proteins into homologous families, Pfam aids researchers in understanding protein function and evolution [35, 38]. To further delve into the structural and functional properties of the HPs, their predicted conserved domains were evaluated by the Conserved Domain Database (CDD) (https://www.ncbi.nlm.nih.gov/Structure/cdd/cdd.shtml) [39]. CDD is an integral part of NCBI’s Entrez search and retrieval system. It enables similarity searches to identify conserved domain structures within the NCBI Entrez protein database. By recognizing these evolutionarily conserved domains, CDD provides valuable insights into the functional properties of proteins [30, 40, 41]. To identify potential homologs and infer the function of HPs, BLASTP (Basic Local Alignment Search Tool for proteins) (https://blast.ncbi.nlm.nih.gov/Blast.cgi) searches were conducted. By comparing the query sequence against a database of known proteins, this analysis can reveal conserved domains and identify potential functional similarities with characterized proteins [42].

### 2.6. Toxicity and Pathogenicity Evaluation of HPs

BTXpred database (https://webs.iiitd.edu.in/raghava/btxpred/) employs machine learning algorithms to analyze the amino acid sequences of proteins and assess the toxicity of HPs. Bacterial toxins are among the primary causes of disease, as they induce damage during infections and are responsible for the manifestation of disease symptoms. Within this platform, toxins are classified into two main categories: exotoxins, which are toxins secreted by bacteria in a soluble form within host tissues, and endotoxins, which are toxins associated with the bacterial cell wall and released into host tissues, ultimately leading to cell death. The system provides accurate and reliable predictions for bacterial toxins and their classifications [43]. Additionally, VICMpred (https://webs.iiitd.edu.in/raghava/vicmpred/), VirulentPred 2.0 (https://bioinfo.icgeb.res.in/virulent2/), and DeepVF (https://deepvf.erc.monash.edu/index.jsp) were utilized to evaluate the pathogenicity of HPs [44]. Understanding the mechanisms of pathogenesis through the analysis of pathogenic factors can serve as a critical step in identifying new and promising therapeutic targets. VICMpred categorizes microbial proteins into four functional classes: disease‐causing agents, metabolism, information storage and processing, and cellular processes [44, 45]. VirulentPred and DeepVF classify proteins into virulent and nonvirulent factors.

### 2.7. Immunoinformatic Analysis of HPs

#### 2.7.1. Antigenicity Assessment

The VaxiJen 2.0 database (https://www.ddg-pharmfac.net/vaxijen/VaxiJen/VaxiJen.html) is a valuable tool for the prediction of conserved antigens based on the physicochemical properties of proteins [46]. Predicting conserved antigens is of paramount importance for the development of effective vaccine candidates [47, 48].

#### 2.7.2. Allergenicity Assessment

In this study, the allergenicity of the investigated proteins was evaluated using the AllerTOP 2.1 (https://www.ddg-pharmfac.net/allertop_test/) and AlgPred 2.0 servers (https://webs.iiitd.edu.in/raghava/algpred2/batch.html). The AllerTOP server predicts allergens based on the main physicochemical characteristics of proteins, while the AlgPred server predicts immune profiles, including the sensitization potential of predicted epitopes, with high accuracy. Both databases were used with protein sequences in plain format [49, 50].

#### 2.7.3. Analysis of Epitopes Recognized by T Cells

Major histocompatibility complex (MHC) molecules are essential for adaptive immunity, enabling the presentation of short peptide fragments known as epitopes to T cells. MHC molecules are classified into two main groups: MHC class I molecules are involved in presenting epitopes to cytotoxic T cells (CD8+ T cells), while MHC class II molecules present epitopes to helper T lymphocytes (CD4+ T cells). Predicting epitopes with high MHC binding affinity is essential for vaccine development [51]. The IEDB database (https://www.iedb.org/) was used to predict MHC‐binding epitopes in the HPs in this study [52]. For this analysis, the default list of alleles provided by the server was employed.

#### 2.7.4. Analysis of Epitopes Recognized by B Cells

The ABCpred (https://webs.iiitd.edu.in/raghava/abcpred/index.html) and Ellipro (http://tools.iedb.org/ellipro/) servers were used to predict continuous and discontinuous epitopes of HPs recognized by B cells. The ABCpred database predicts linear epitopes of B lymphocytes with an accuracy of over 65%. On the other hand, the Ellipro server predicts discontinuous epitopes of B lymphocytes based on the geometrical properties of protein structures. For the ABCpred server, protein sequences were uploaded in plain format, while the tertiary structures of HPs were uploaded in PDB format for the Ellipro server [53, 54].

#### 2.7.5. Active Site Determination

The Computed Atlas of Surface Topography of the universe of protein Folds (CASTpFold) (https://cfold.bme.uic.edu/castpfold/compute) server was used to predict the active sites for candidate HPs. This enhanced CASTp server poses new features for the whole protein universe [55]. Pockets were also predicted by PrankWeb 4 (https://prankweb.cz/), a web server for protein–ligand binding site prediction [56]. Top I‐TASSER predicted model for each HP was uploaded to these servers in standard PDB format.

## 3. Results

### 3.1. Sequence Retrieval

Among 181 resistant strains of *P. aeruginosa*, 35 carbapenem‐resistant strains with finished genomes were detected as previously mentioned [3] and 15 common HPs were selected for further investigation (Table 1).

**Table 1:** 15 common HPs among resistant strains of *P. aeruginosa.*

**Name of HPs**	**Amino acid sequence length of HPs**	**IMG gene ID**
HP1	204	2877781935
HP2	206	2877781443
HP3	218	2877782511
HP4	239	2877782084
HP5	250	2877781665
HP6	277	2877781545
HP7	303	2877782616
HP8	334	2877782174
HP9	346	2877781709
HP10	354	2877781914
HP11	356	2877781791
HP12	444	2877781478
HP13	455	2877782544
HP14	459	2877781704
HP15	461	2877781705

### 3.2. Primary Structural Analysis of HPs

Physicochemical properties, including molecular weight, isoelectric point (pI), instability index, aliphatic index, and grand average of hydropathicity (GRAVY) value were computed for each of the 15 HPs through ProtParam (Table 2). The subcellular localization of HPs, the presence of their transmembrane helices predicted by DeepTMHMM and CCTOP, the presence of signal peptides predicted by SignalP, and the potential of nonclassical secretory pathway use predicted by SecretomP were also depicted in Table 3. DeepTMHMM, which is a newer version of TMHMM 2.0, also predicted HP6 and HP12 as *β*‐barrel proteins with no helices.

**Table 2:** Physicochemical properties of investigated HPs.

**Name**	**Molecular weight**	**Instability index**	**Isoelectric point (pI)**	**Aliphatic index**	**GRAVY**
HP1	22484.09	47.99	5.24	78.68	−0.417
HP2	22367.34	44.84	7.65	76.21	−0.316
HP3	24402.24	43.18	9.05	132.94	−0.833
HP4	27198.07	53.33	9.69	89.04	−0.586
HP5	27021.72	37.71	6.75	100.88	−0.172
HP6	31071.01	20.88	9.28	62.71	−0.467
HP7	33540.33	52.43	4.57	69.67	−0.477
HP8	36357.45	34.43	5.01	96.71	0.041
HP9	37811.61	51.44	5.79	98.06	−0.003
HP10	41087.08	58.43	7.66	97.01	−0.444
HP11	40858.60	42.08	6.50	94.10	−0.333
HP12	48840.71	29.76	4.97	70.11	−0.482
HP13	51229.54	33.96	8.57	76.18	−0.564
HP14	49950.90	35.57	6.26	105.14	−0.233
HP15	49655.53	49.85	6.92	119.15	0.168

**Table 3:** Primary structural properties for investigated HPs.

**Name**	**PSORTb**	**CELLO II**	**PSLpred**	**Gneg-mPLoc**	**TMHMM**	**CCTOP**	**SignalP**	**SecretomP**
HP1	N	C	C	P	No	No	No	No
HP2	N	P	P	P	No	No	Yes	Yes
HP3	CM	IM	IM	IM	Yes	Yes	No	No
HP4	C	C	C	IM. C	Yes	Yes	No	No
HP5	N	P	P	E. P	No	No	Yes	No
HP6	N	OM	OM	OM	No	No	Yes	Yes
HP7	N	P	C	P	No	No	No	Yes
HP8	C	C	C	IM. C	No	No	No	No
HP9	N	IM	P	IM	No	No	Yes	No
HP10	N	C	C	IM	No	No	No	No
HP11	C	C	C	IM	No	No	No	No
HP12	OM	OM	OM	OM	No	No	Yes	Yes
HP13	N	P	P	IM	No	No	Yes	Yes
HP14	C	C	C	C	No	No	No	No
HP15	CM	IM	C	IM	Yes	Yes	No	No

*Note:* The presence or absence of transmembrane helices by DeepTMHMM and CCTOP, signal peptide by SignalP and nonclassical secretory pathway by SecretomP were indicated by Yes and No.

Abbreviations: C: cytoplasmic, IM: inner membrane, N: unknown, OM: outer membrane, P: periplasmic.

### 3.3. Secondary and Tertiary Structural Analysis of HPs

**Figure 2:**
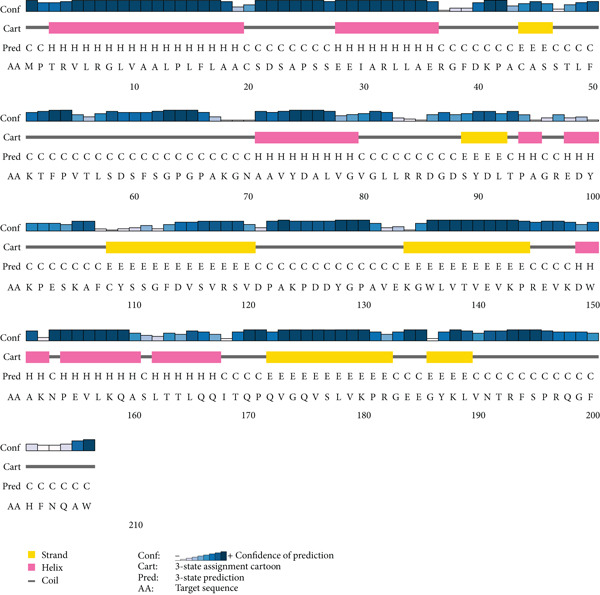
The secondary structure of HP2 predicted by PSIPRED server. The predicted secondary structures for other HPs are given in Figures S1 and S2.

**Figure 3:**
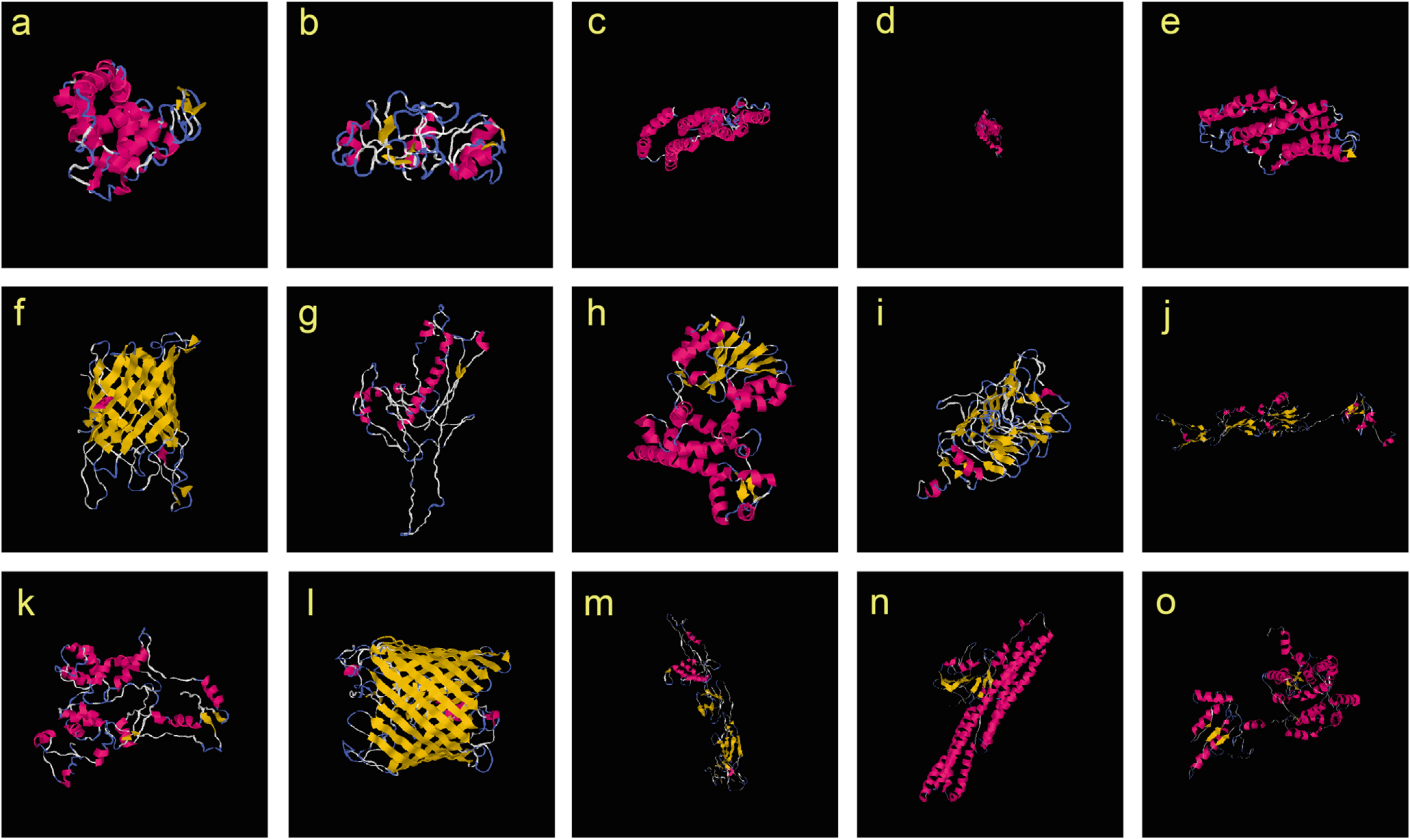
(a‐o) Tertiary structural models predicted by I‐TASSER server for 15 HPs.

The prediction of secondary structures performed by SOPMA and PSIPRED revealed three states of helices, strands, and coils for investigated HPs based on their amino acid sequences. The SOPMA server also agreed with the PSIPRED’s evaluation (Figure 2 and Figures S1 and S2). The tertiary structural models predicted by I‐TASSER server for HPs are shown in Figure 3. All the tertiary structures obtained a score above 50 in the ERRAT tool, confirming the quality and reliability of these predicted three‐dimensional structures.

### 3.4. Functional Analysis of HPs

The functional domains of HPs predicted by SMART and Pfam servers along with their conserved domains predicted by the CDD database are presented in Table 4. Notably, both SMART and Pfam yielded identical results for most HPs. HP1 belongs to DMP19 protein family, which contains DMP19 conserved protein in Neisseria, acting as a DNA mimic. This protein family was formerly known as DUF4375 [57]. HP6 was predicted to have a nucleoside‐specific outer membrane channel protein Tsx, functioning as a substrate‐specific channel for nucleosides and deoxy‐nucleosides. HP8 was predicted to have dimerization domain found in some O‐methyltransferases, and Methyltransf_2 domain, which functions as O‐methyltransferase. HP12 was predicted to contain outer membrane porin D (OprD) domain, indicating porin activity. DUF1329 and LolA_like family were identified for HP13. The first domain is a protein of unknown function and the latter contains uncharacterized proteins similar to the several periplasmic and outer membrane proteins. DUF7847, DUF6776, DUF3482, and DUF2868 predicted, respectively, for HP3, HP14, and HP15 have also an unknown function. Another domain predicted for HP14 was MMR_HSR1, which is a GTPase and GTP binding protein in related to 50s Ribosome. No functional or conserved domain was predicted for HP2, HP5, HP7, and HP9‐HP11.

**Table 4:** The predicted functional and conserved domains of HPs.

**Name**	**SMART**	**Pfam**	**CDD**
HP1	DUF4375	DMP19	DUF4375 (DMP19 family protein)
HP2	—	—	—
HP3	—	DUF7847	—
HP4	DUF6776	DUF6776	DUF6776
HP5	—	—	—
HP6	—	—	Tsx
HP7	—	—	—
HP8	Demerisation2 Methyltransf_2	Demerisation2 Methyltransf_2	Demerization2 Methyltransf_2
HP9	—	—	—
HP10	—	—	—
HP11	—	—	—
HP12	OprD	OprD	OprD
HP13	DUF1329	DUF1329	LolA_fold‐like
HP14	MMR_HSR1 DUF3482	MMR_HSR1 DUF3482	MMR_HSR1 DUF3482
HP15	DUF2868	DUF2868	DUF2868

Amino acid sequences alignment of 15 HPs through BLASTP identified homologous sequences with uncharacterized proteins in other bacterial species f33or the majority of the investigated HPs (Table S1). None of these 15 HPs were predicted to be exotoxins by the BTXpred server. Pathogenicity prediction using VICMpred database classified HP2, HP3, HP4, and HP12 as metabolism‐related proteins, while HP14 and HP15 were predicted as information and storage proteins. The remaining HPs were categorized under cellular processes. All HPs except HP1 were also predicted as virulent factors by both VirulentPred and DeepVF. Among these, HP2 and HP5 exhibited the highest probability scores.

### 3.5. Immunoinformatic Analysis

The antigenicity evaluation by the VaxiJen database identified HP2, HP3, HP4, HP6, HP7, HP9, HP12, and HP13 as antigenic proteins. In this database, a hypothetical threshold of above 0.4 is used to classify proteins as antigens. The allergenicity assessment of the HPs using the AllerTOP and Algpred servers revealed that HP1, HP4, HP9, and HP10 were identified as allergens by the AllerTOP server, while Algpred predicted HP12 and HP13 as allergens. Epitopes recognized by T and B lymphocytes are given in the Tables S2 and S3, respectively.

### 3.6. Active Site Determination

**Figure 4:**
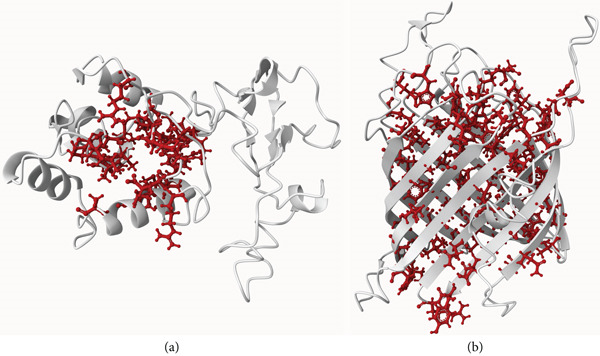
The top ranked active site predicted by PrankWeb for (a) HP2 and (b) HP6.

The CASTpFold server predicted 20 and 37 different active sites for HP2 and HP6, respectively. All surface pockets were recognized with this server and all atoms involved in their formation were described. The exact size and area for each pocket were also calculated. The top active site of the HP2 was identified with the surface area of 503.022 and the surface volume of 288.796 Å^2^. The top active site of the HP6 was predicted with the area of 1450.878 and the volume of 1007.724 Å^2^. Active site prediction by PrankWeb revealed six binding pockets for both HP2 and HP6 with the scores of 14.74 and 125.31 and the probabilities of 0.741 and 0.998 for their top ranked pockets, respectively (Figure 4). The top pockets for HP2 and HP6 predicted by both above servers was the same.

## 4. Discussion

Through a comparison of genomic sequences of MDR, XDR, and carbapenem‐resistant strains of *P. aeruginosa* with the finished genome sequences, 15 common HPs were selected for further analysis. The analyzed HPs were not present in either humans or the human microbiome, making them preferable targets for species‐specific antibacterial therapeutics and substantially reducing the likelihood of cross‐reactivity with host proteins that could potentially induce autoimmune responses or other adverse effects. Furthermore, lacking homology to human microbiome proteins is an important consideration since disruption of gut microbiota can lead to severe health complications [30]. The amino acid sequence and total length of a protein are critical factors influencing its structures and functions. Shorter proteins often fail to fold properly due to an insufficient number of interactions between residues, whereas longer proteins have a greater likelihood of forming multiple secondary and tertiary structures [58]. Consequently, in this study, HPs with the lengths exceeding 200 amino acids were selected for analysis. Since HPs are abundant proteins and may play important roles in the survival and pathogenicity of a pathogen, there is a clear need for the functional annotations of these type of proteins [59].

A protein’s function is closely tied to its location within the cell, making the prediction of its subcellular localization a critical step in understanding its biological role. Furthermore, a protein’s localization can indicate its potential as a drug or vaccine candidate. For instance, cytoplasmic proteins may serve as intracellular therapeutic targets, whereas membrane or cell surface proteins are often ideal candidates for vaccine development [30, 60]. In bacterial cells, outer membrane proteins, in particular, have been claimed as highly effective candidates for inducing immunity [47, 61]. The databases used in this study for subcellular localization prediction employ diverse computational approaches, achieving prediction accuracies exceeding 90% [19–21, 62].

Additionally, the physicochemical properties of proteins play a crucial role in drug and vaccine candidate selection. The charge distribution of a protein, which is influenced by its subcellular environment, reflects regulatory adaptations to specific pH and ionic conditions. Numerous examples demonstrate how proteins undergo regulatory modifications to maintain stability and function under particular pH conditions. This suggests that structural adaptations are likely optimized for the unique physicochemical environment of each subcellular compartment [63]. In this study, all investigated HPs except HP2, HP3, HP4, HP6, HP10, and HP13 were acidic, as indicated by their predicted pI. HP3 exhibited the highest thermostability, as reflected by its elevated aliphatic index—a metric positively correlated with protein stability at higher temperatures. Hydrophobicity analysis via the GRAVY index revealed that HP3, HP8, and HP15 displayed positive values, suggesting hydrophobic properties. Transmembrane helices predictions based on both DeepTMHMM and CCTOP servers, further identified HP3, HP4, and HP15 as transmembrane proteins. DeepTMHMM also predicted HP6 and HP12 as *β*‐barrel proteins, confirming their predicted locations in outer membrane and their tertiary models by I‐TASSER (Figure 3). In contrast, HP2, HP5, HP6, HP9, HP12, and HP13 were predicted to contain signal peptides through SignalP, implicating their involvement in classical secretory pathways. Notably, HP2, HP6, HP7, HP12, and HP13 likely follow nonclassical secretion, as their sequences scores exceeded the threshold of 0.5 (Table 3).

Another critical step in inferring the biological function of a protein is the identification of its structure [30]. Protein structure directly determines its biological activity as even subtle structural changes modulate protein function [64]. Moreover, accurate structure and function prediction can facilitate the identification of novel targets for therapeutic development [65]. The secondary structure of a protein, which is primarily determined by interactions between adjacent amino acid residues, serves as the foundation for its three‐dimensional structure, as the folding of secondary structural elements ultimately gives rise to the protein’s tertiary structure [28]. In this study, HP1, HP3, HP4, HP5, HP8, HP11, HP14, and HP15 were predicted to have a greater proportion of Alpha‐helices but HP2, HP6, HP7, HP9, HP10, HP12, and HP13 were identified to have a greater proportion of random coils (Figure S1 and Figure S2). Functional and conserved domain analysis of HPs revealed conserved domains for HP1, HP3, HP4, HP6, HP8, HP12, HP13, HP14, and HP15 by different servers (Table 4). The lack of conserved domains for remaining HPs may reflect current limitations in analytical servers and databases. Advancements in computational algorithms and updating domain databases annotate domains in proteins that previously unresolved. Furthermore, domain prediction is predominantly based on sequence similarities but conserved structural regions often exceed sequence conservation. In addition, novel proteins may lack sufficient similarity to known domains [66]. BLASTP results also agreed mostly with domain prediction results for above HPs.

The identification of immunogenic T‐ and B‐cell epitopes constitutes another fundamental step for vaccine design. Computational prediction of conserved epitopes enables the precise selection of antigenic regions that elicit robust immune responses, while simultaneously excluding non‐immunogenic sequences. This bioinformatic approach significantly enhances the efficiency of downstream experimental validation by minimizing both developmental time and production costs [67].

**Figure 5:**
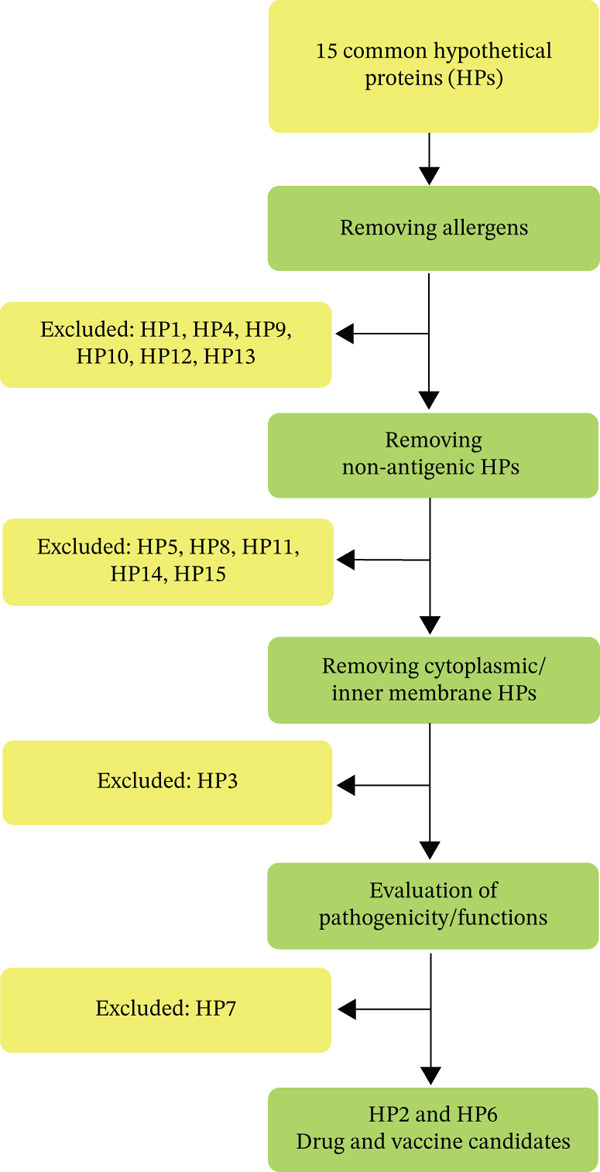
The multistep filtering strategy for prioritizing hypothetical proteins (HPs).

Among the initial investigated HPs, a systematic and multistep filtering strategy was employed to prioritize candidate HPs for drug and vaccine development (Figure 5). The selection was based on the five most critical factors established in the literatures for effective drug and vaccine candidates: (1) localization, (2) antigenicity, (3) non‐allergenicity to human, (4) functional annotation, and (5) exotoxin properties [68, 69]. HP1, HP4, HP9, HP10, HP12, and HP13 were excluded immediately due to their documented allergenic properties. HPs lacking antigenic potential (HP5, HP8, HP11, HP14, and HP15) were also discarded. Antigenicity is a prerequisite for inducing immunity. The absence of antigenicity in HP1 and HP10 was another reason to exclude these two proteins. The remaining HPs were assessed for subcellular localization and functional annotation. Cytoplasmic proteins and inner membrane proteins are generally unsuitable for drug and vaccine development due to their inaccessibility and challenges in extraction [70]. Moreover, determining the three‐dimensional structure of inner membrane proteins under native conditions is inherently difficult, hindering the identification of active sites crucial for drug targeting [71]. The intricate biogenesis and membrane integration processes of these proteins further impede effective drug interaction [72]. Consequently, the predicted inner membrane protein of HP3 was excluded from the final candidate selection. The predicted cytoplasmic location for HP1, HP10, HP14, and HP15 also resulted in their exclusion. Proteins involved in cellular processes may serve as viable candidates for vaccines or therapeutics only if they fulfill two key criteria: (1) a functional role in pathogenicity and (2) localization in the outer membrane. Thus, HP7 was disqualified because it resides in the periplasmic space rather than the outer membrane, leading to its removal from the pool of potential candidates [73, 74].

Following the above stringent filtering, HP2 and HP6 (Gene ID: 2877781443 and 2877781545) were finally selected as potential drug and vaccine candidates, respectively. They were both non‐allergenic and exhibited antigenicity with the scores of 0.54 and 0.60, respectively. HP2 was identified as a periplasmic secretory protein while HP6 was localized to the outer membrane. Neither was predicted to be an exotoxin. The absence of transmembrane helices in both HP2 and HP6 suggest favorable characteristic, facilitating their purification. HP6 with its outer membrane localization, higher stability and superior antigenicity was selected as our promising vaccine candidate in this study. Although, the secretory protein of HP2 also fulfils the above five criteria for a vaccine candidate, but its periplasmic location along with the highest predicted probability of being a virulent factor among other investigated HPs, designate it as the foremost therapeutic target identified in this research. Periplasmic proteins are clearly more accessible than cytoplasmic proteins and inhibition of virulent proteins involved in metabolism, may be helpful to combat infections [75]. BLASTP analysis confirmed the conservation of both HP2 and HP6 across some other gram‐negative, drug‐resistant bacteria especially *Acinetobacter baumannii*, suggesting their potential as broad‐spectrum vaccine and therapeutic candidates. This sequence analysis provides a framework for elucidating sequence–function relationships and enhance our understanding of molecular mechanisms in organisms.

## 5. Conclusion

*P. aeruginosa* represents a significant public health concern, with carbapenem‐resistant strains prioritized by the World Health Organization as a critical target for the development of new therapeutics. Given the potential biological functions of HPs, this study focused on their potential as novel therapeutic drug and vaccine candidates. Through the structural characterization and functional annotation of 15 common HPs across MDR strains of *P. aeruginosa*, two HPs (gene ID: 2877781443 and 2877781545) were computationally predicted as potential candidates for drug and vaccine development. While our comprehensive predictive analysis highlights the value of integrating genomic data with structural and functional annotation for target identification, further experimental validation is required. Concrete next steps include recombinant protein expression, epitope mapping, and immunogenicity assays to confirm the therapeutic potential of these candidates against *P. aeruginosa* infections.

## Conflicts of Interest

The authors declare no conflicts of interest.

## Author Contributions

Fatemeh Nasiri Khoonsari performed the analysis and prepared the draft of manuscript. Zahra Zafari designed the study and revised the manuscript.

## Funding

No funding was received for this manuscript.

## Supporting Information

Additional supporting information can be found online in the Supporting Information section.

## Supporting information


**Supporting Information 1** Figure S1. Secondary structures of hypothetical proteins predicted by SOPMA.

**Supporting Information 2** Figure S2. Secondary structures of hypothetical proteins predicted by PSIPRED.

**Supporting Information 3** Table S1. BLASTP analysis results of hypothetical proteins.

**Supporting Information 4** Table S2. T Cell epitopes identified in hypothetical proteins.

**Supporting Information 5** Table S3. B cell epitopes identified in hypothetical proteins.

## Data Availability

The data that supports the findings of this study are included within this article and also available upon request.
