# Supplementary material for: Structural Characterization and Functional Annotation of Hypothetical Proteins in the Multidrug‐Resistant Strains of Pseudomonas aeruginosa
Source: Biomed Res Int. 2026 Feb 2;2026:2974616. doi: 10.1155/bmri/2974616 (PMC12864544; doi:10.1155/bmri/2974616)

Supplementary Figure S1. Secondary structures of hypothetical proteins predicted by SOPMA

**HP1:**


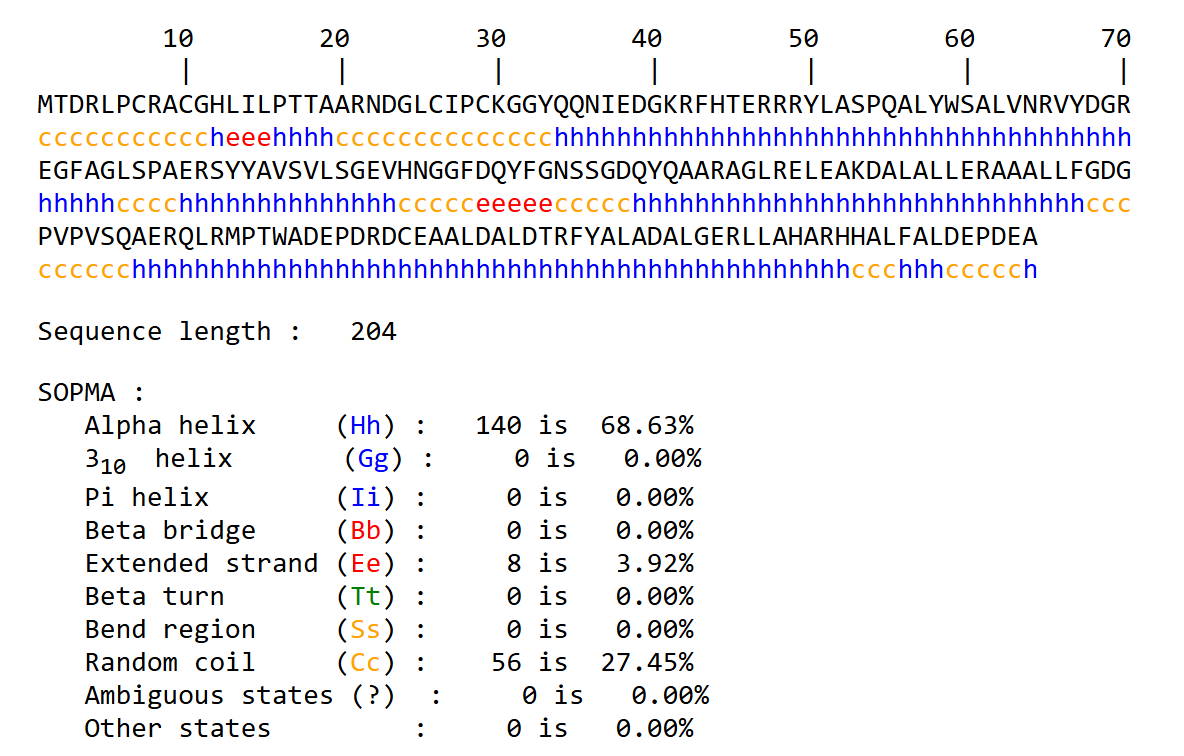


**HP2:**


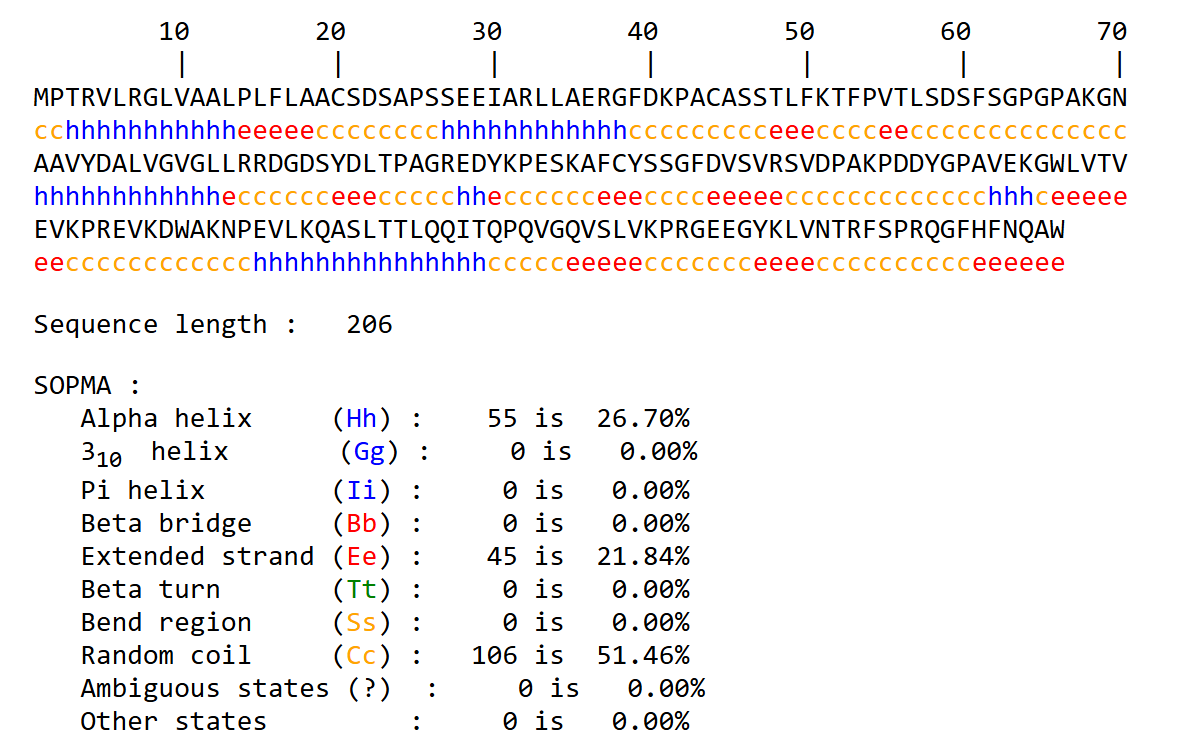


**HP3:**


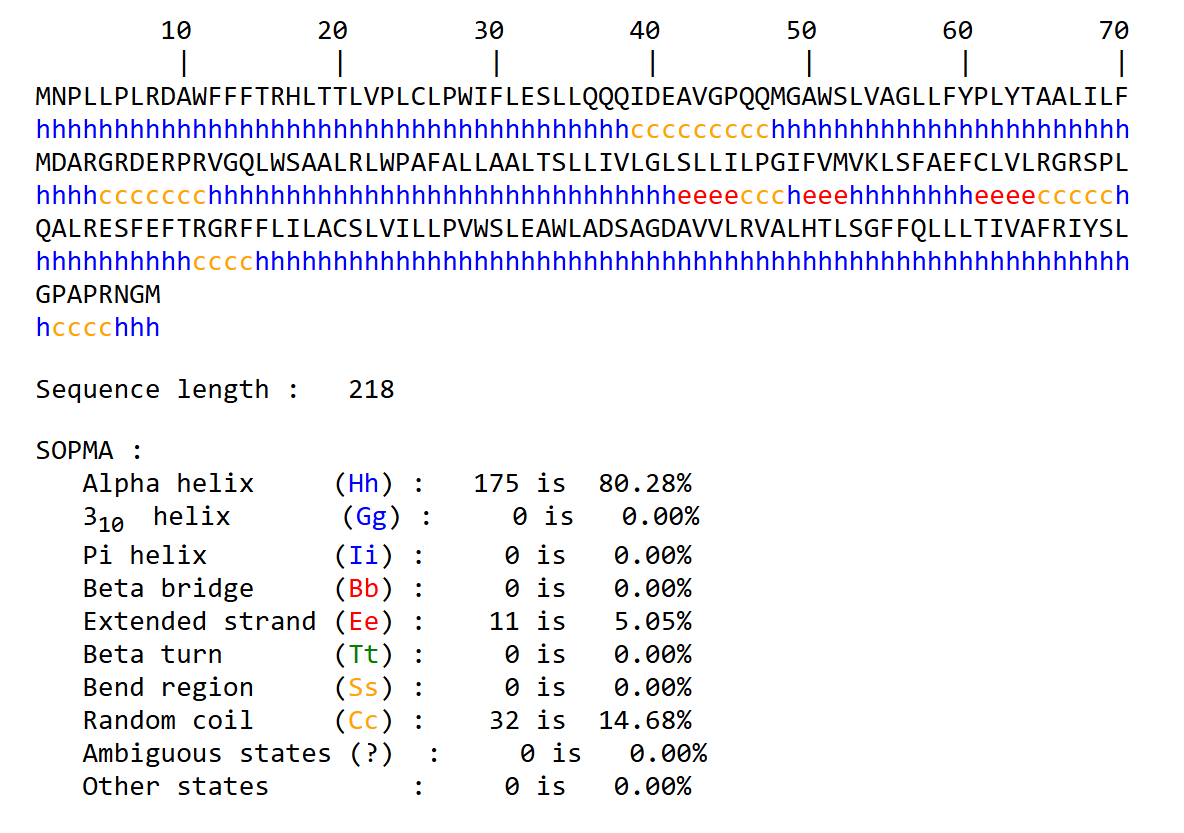


**HP4:**


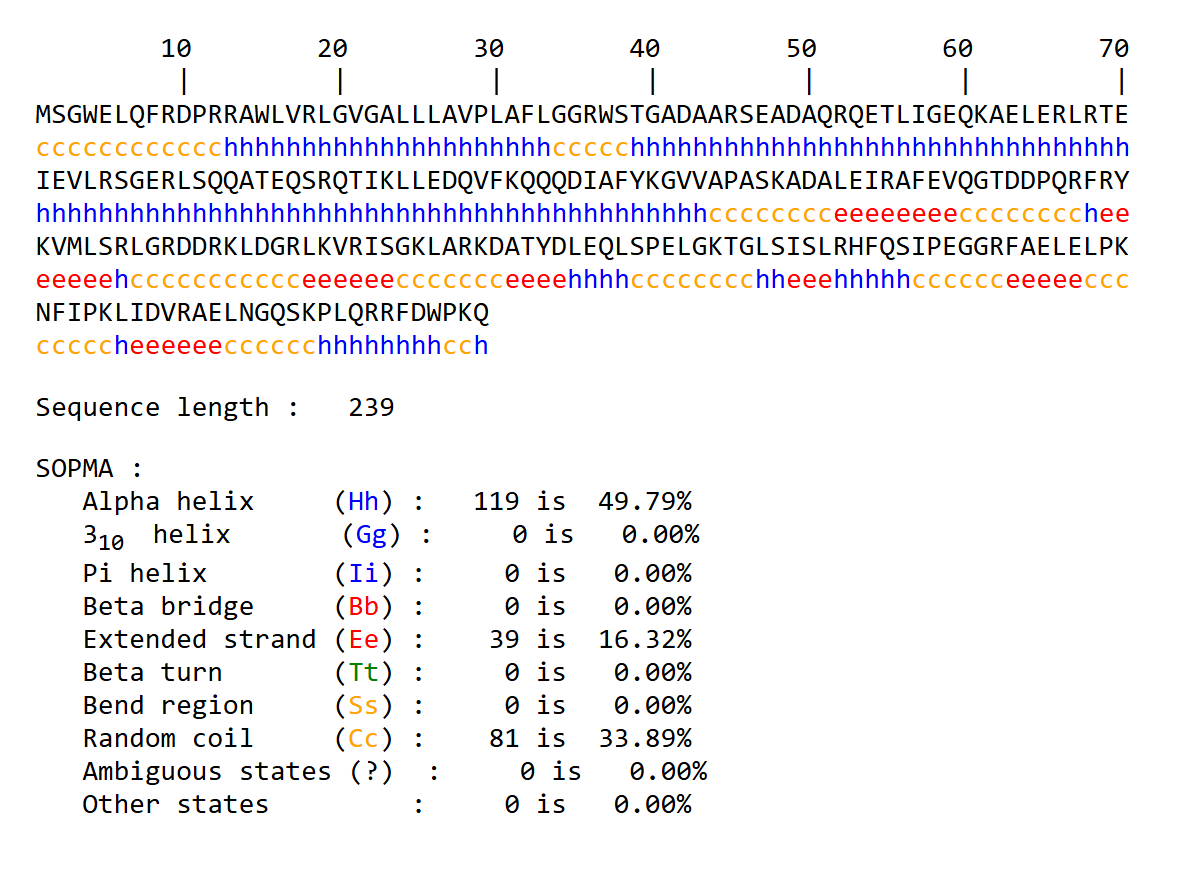


**HP5:
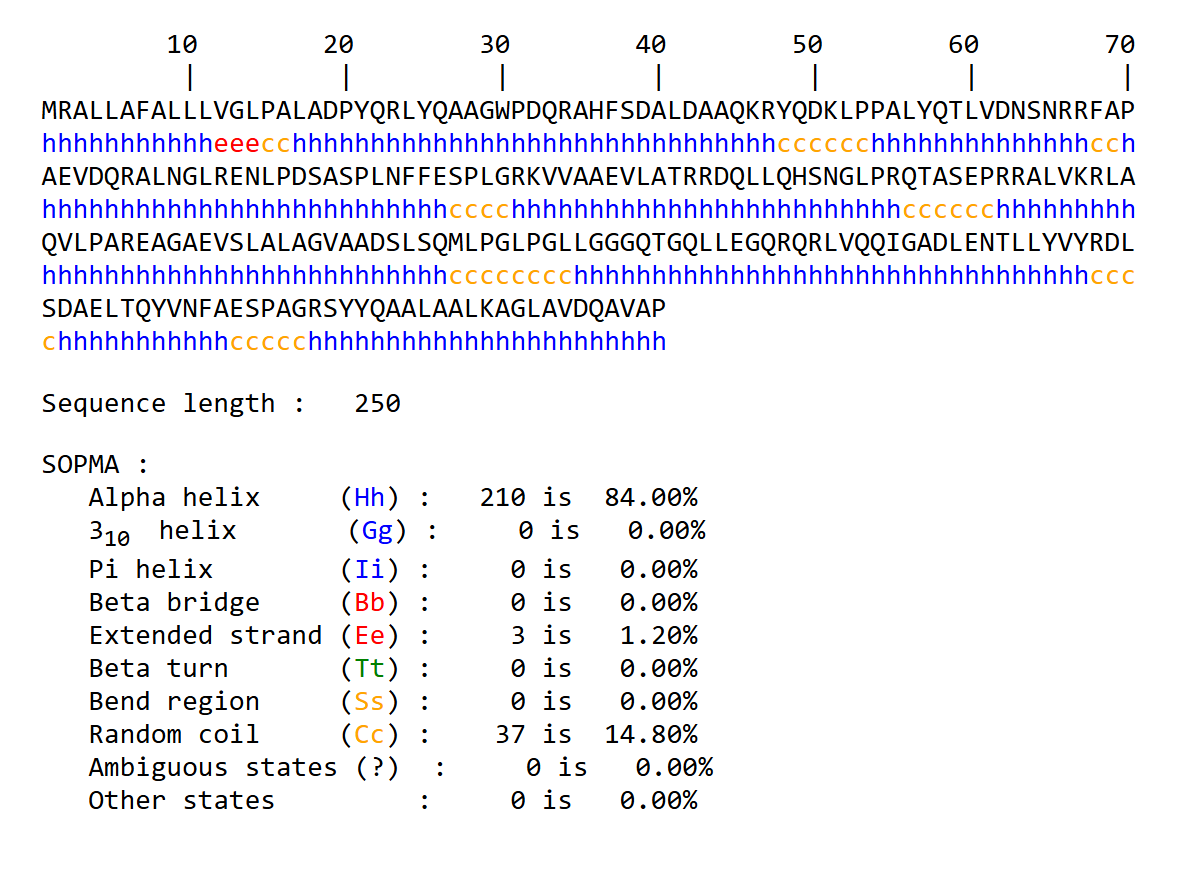
**

**HP6:
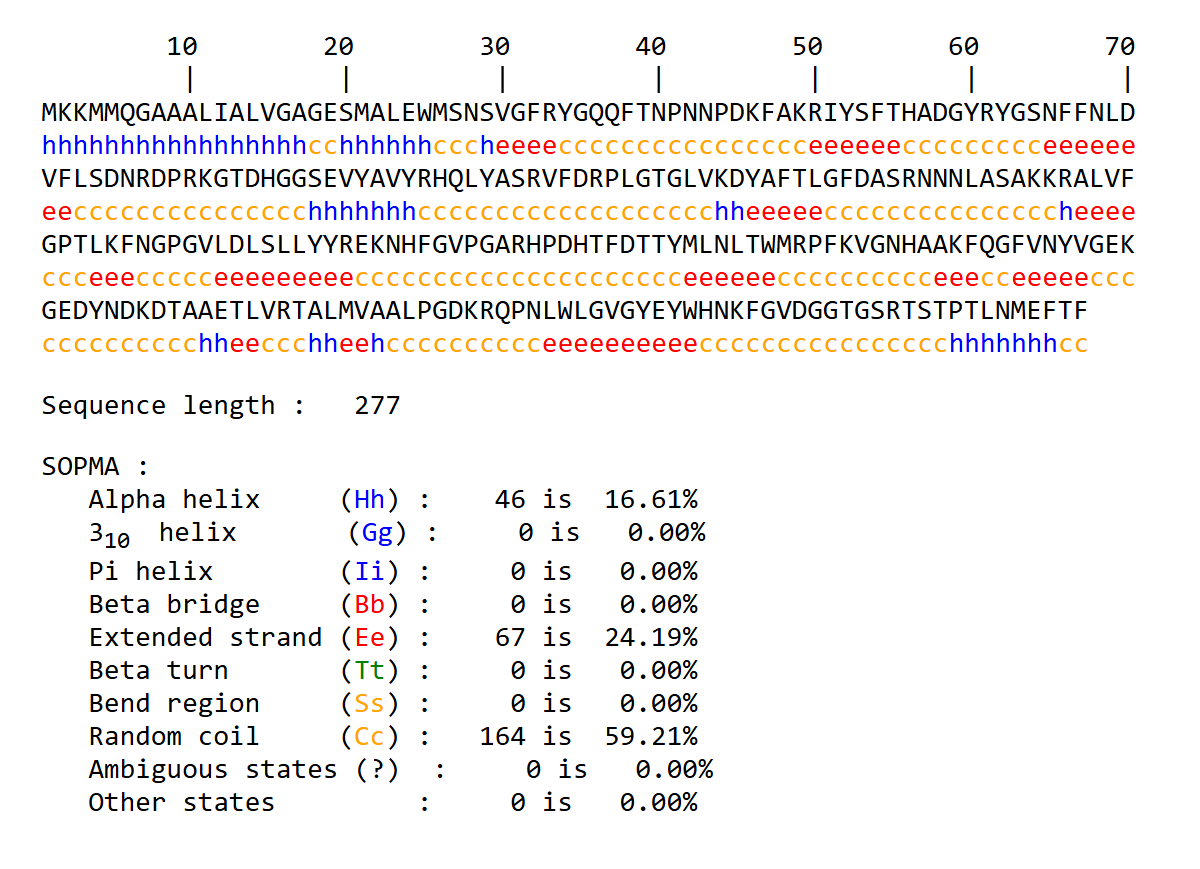
**

**HP7:**


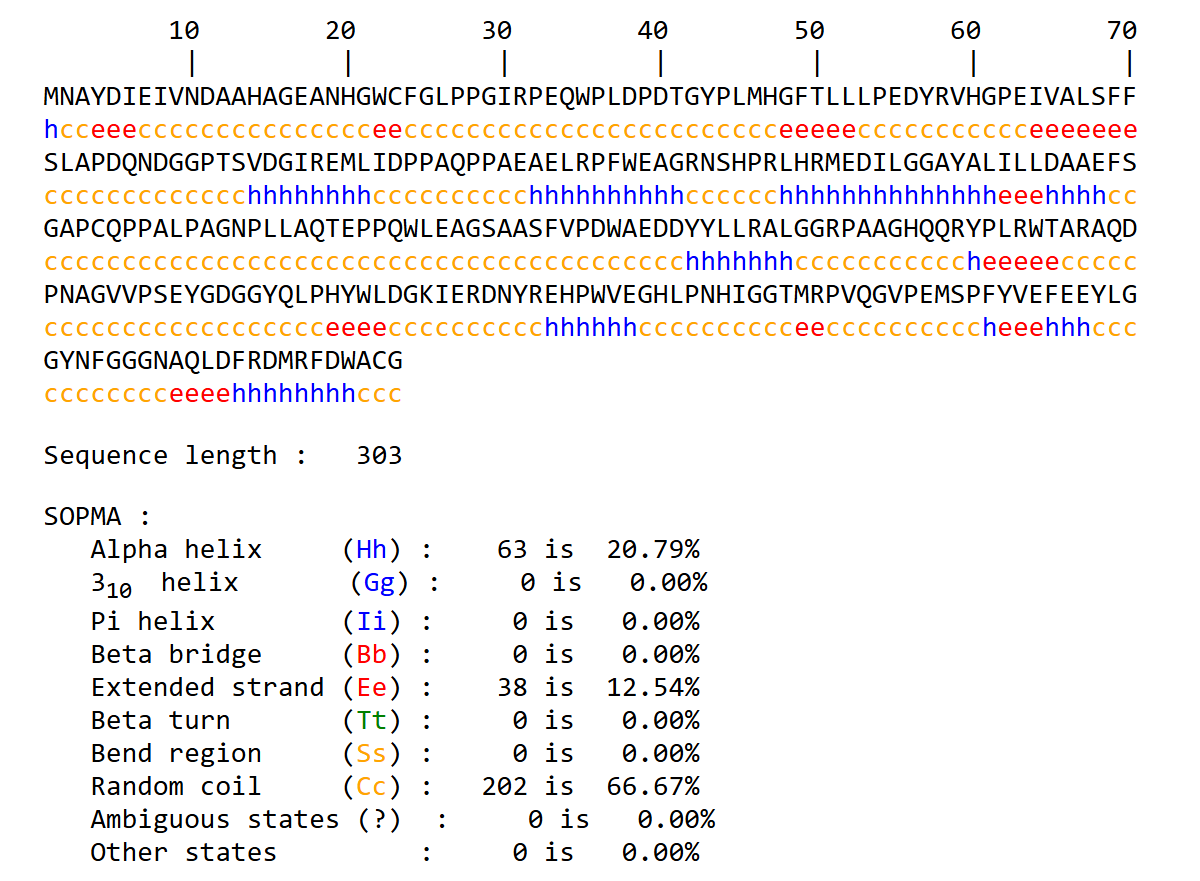


**HP8:
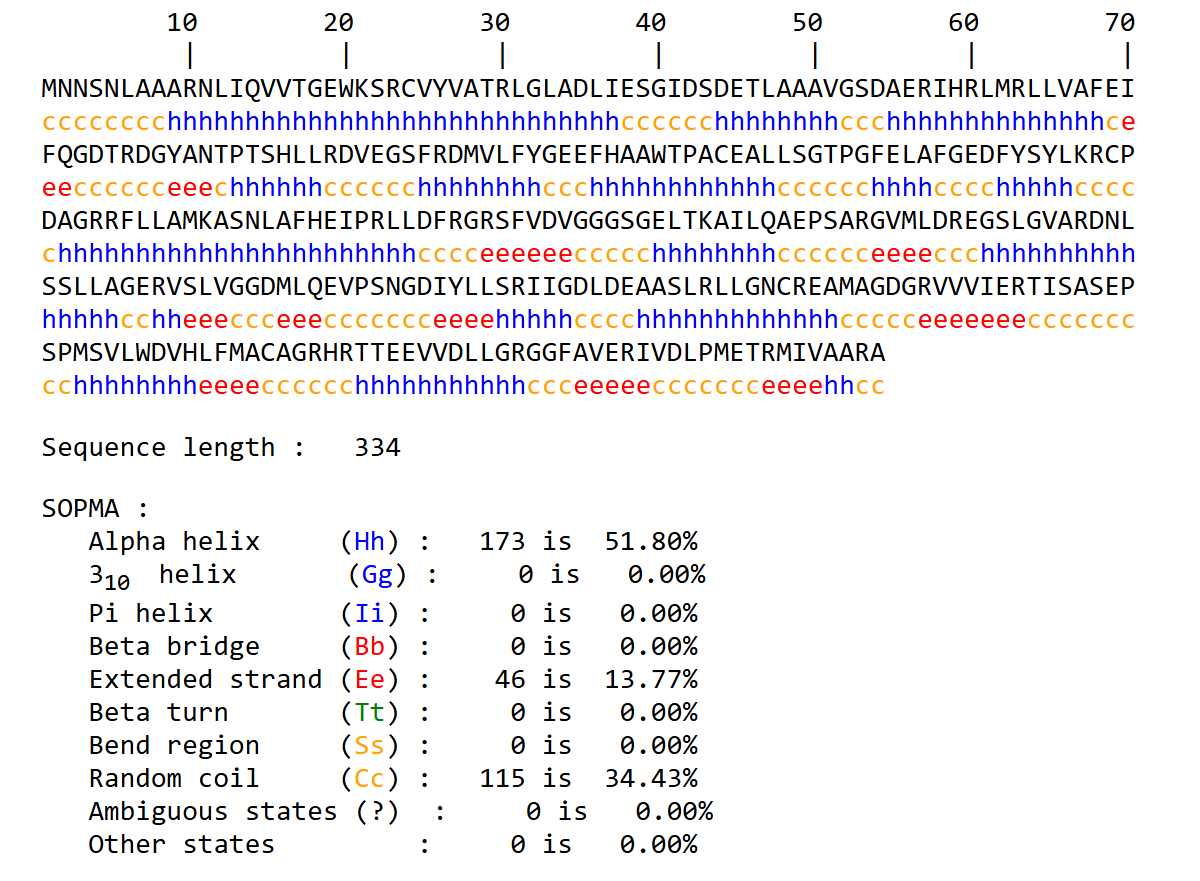
**

**HP9:
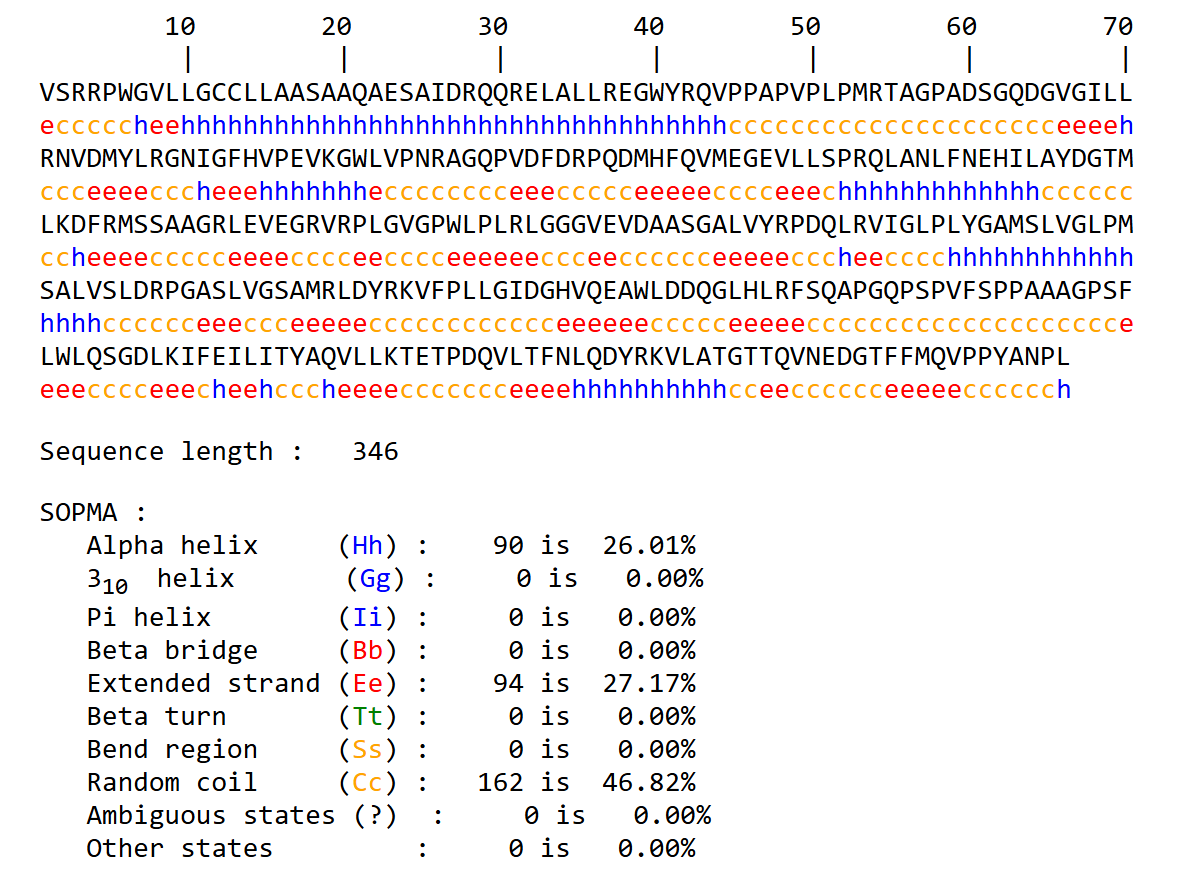
**

**HP10:**

**
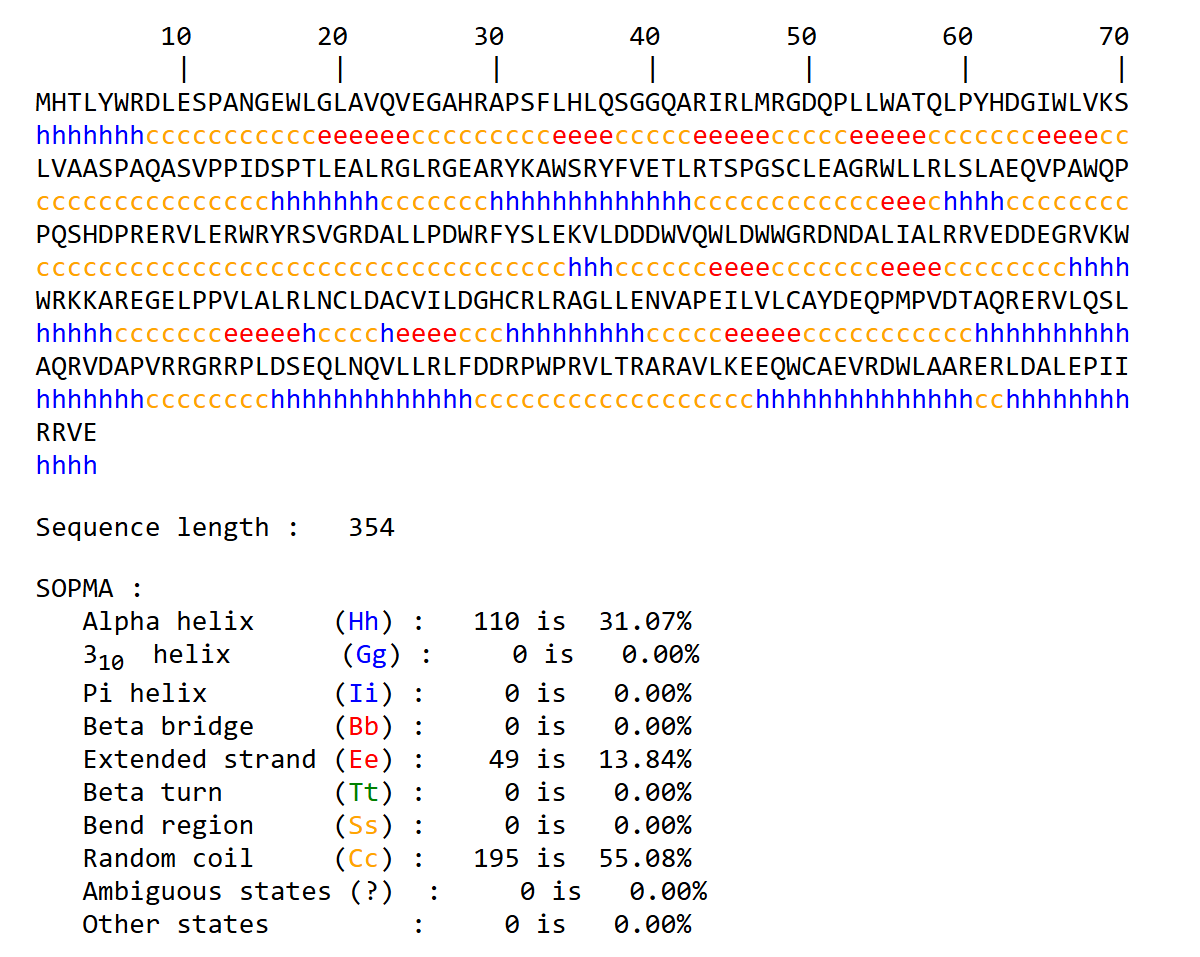
**

**HP11:**

**
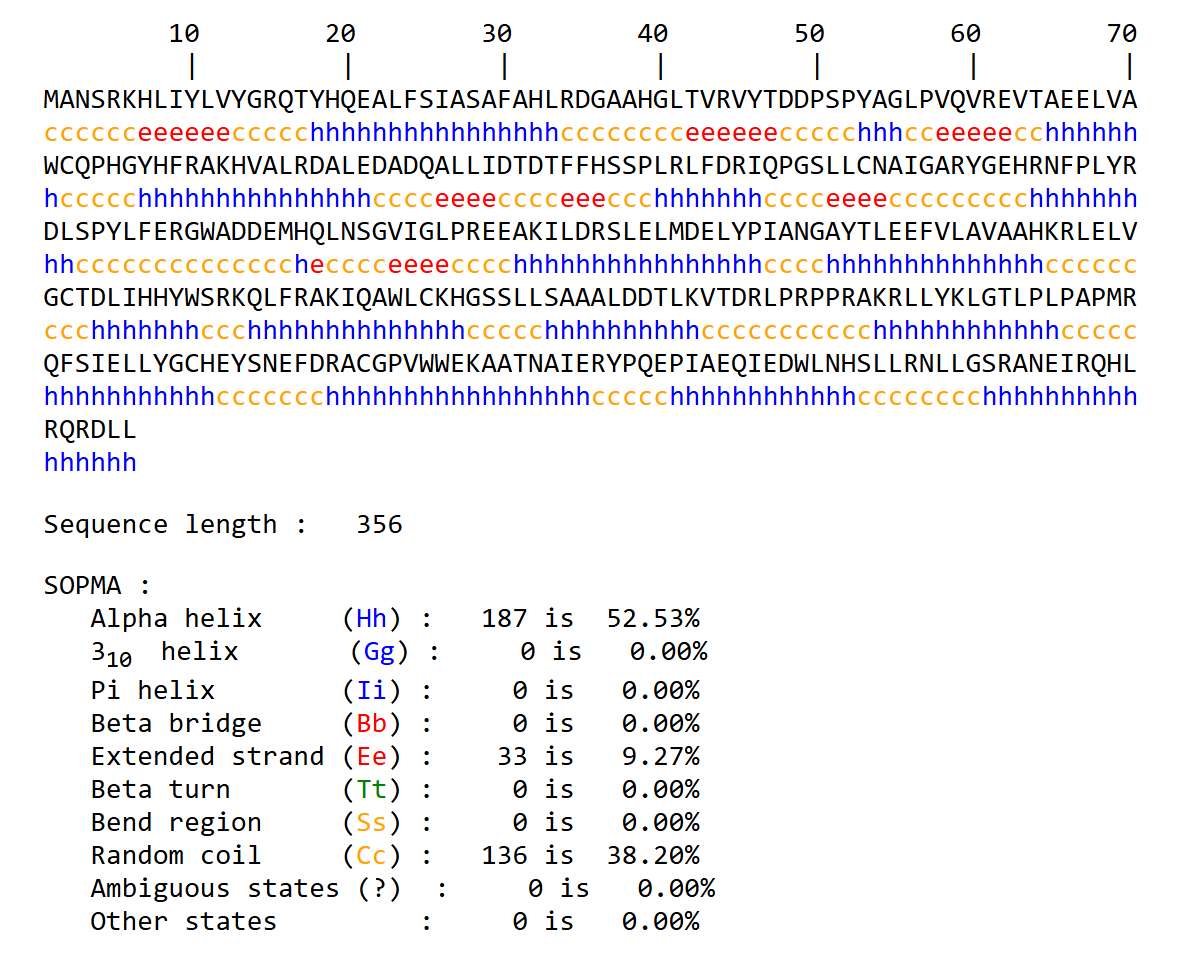
**

**HP12:
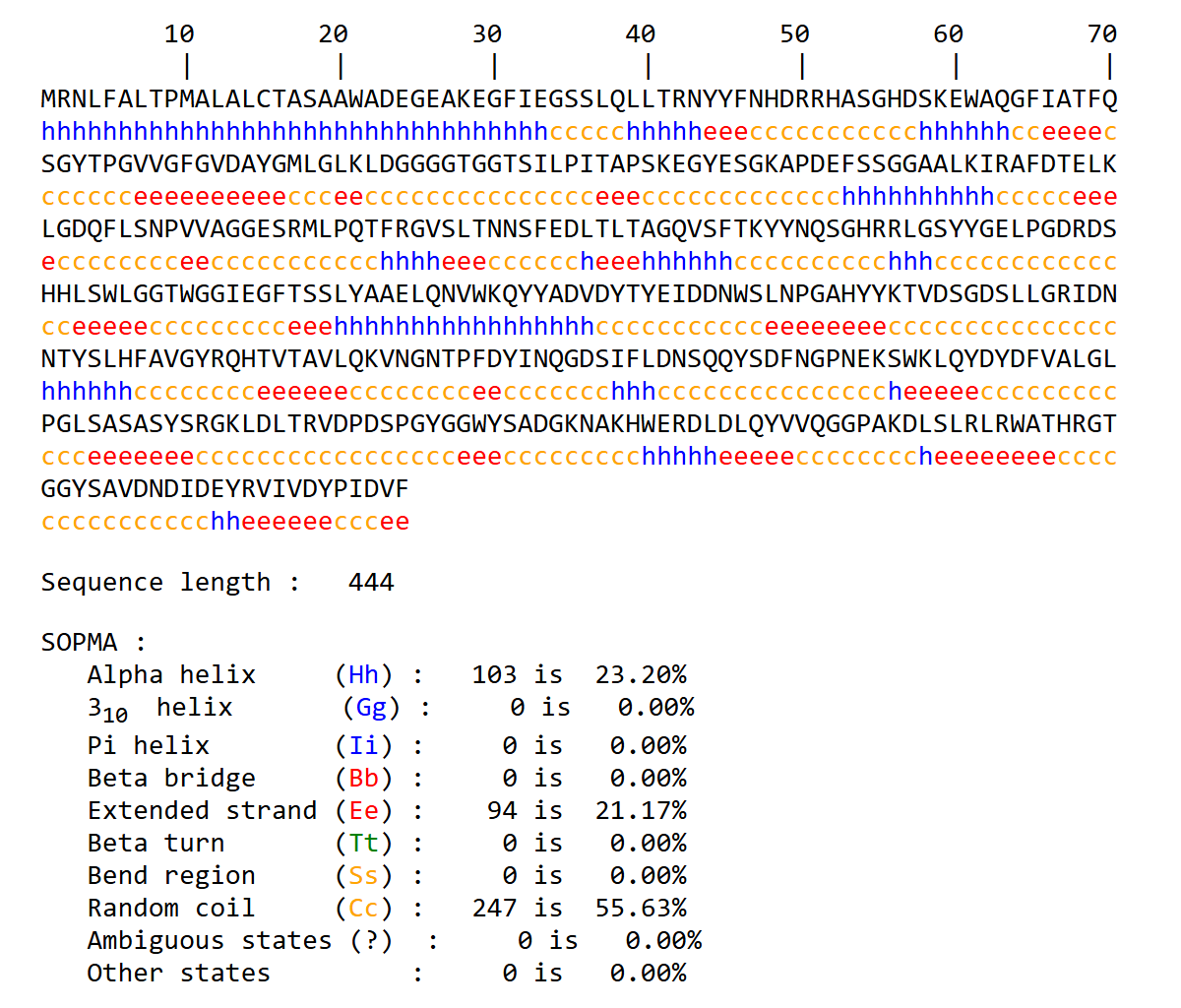
**

**HP13:
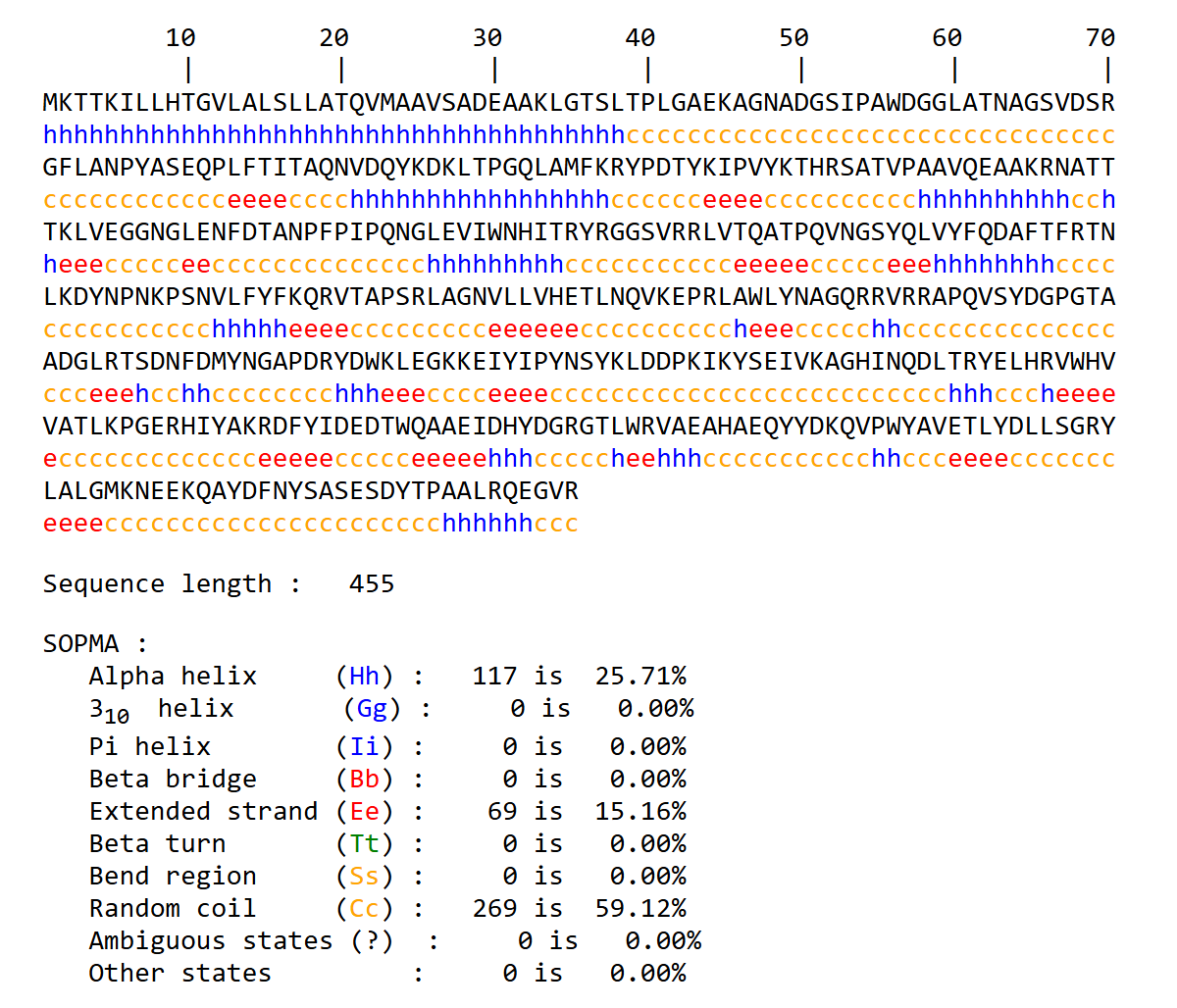
**

**HP14:
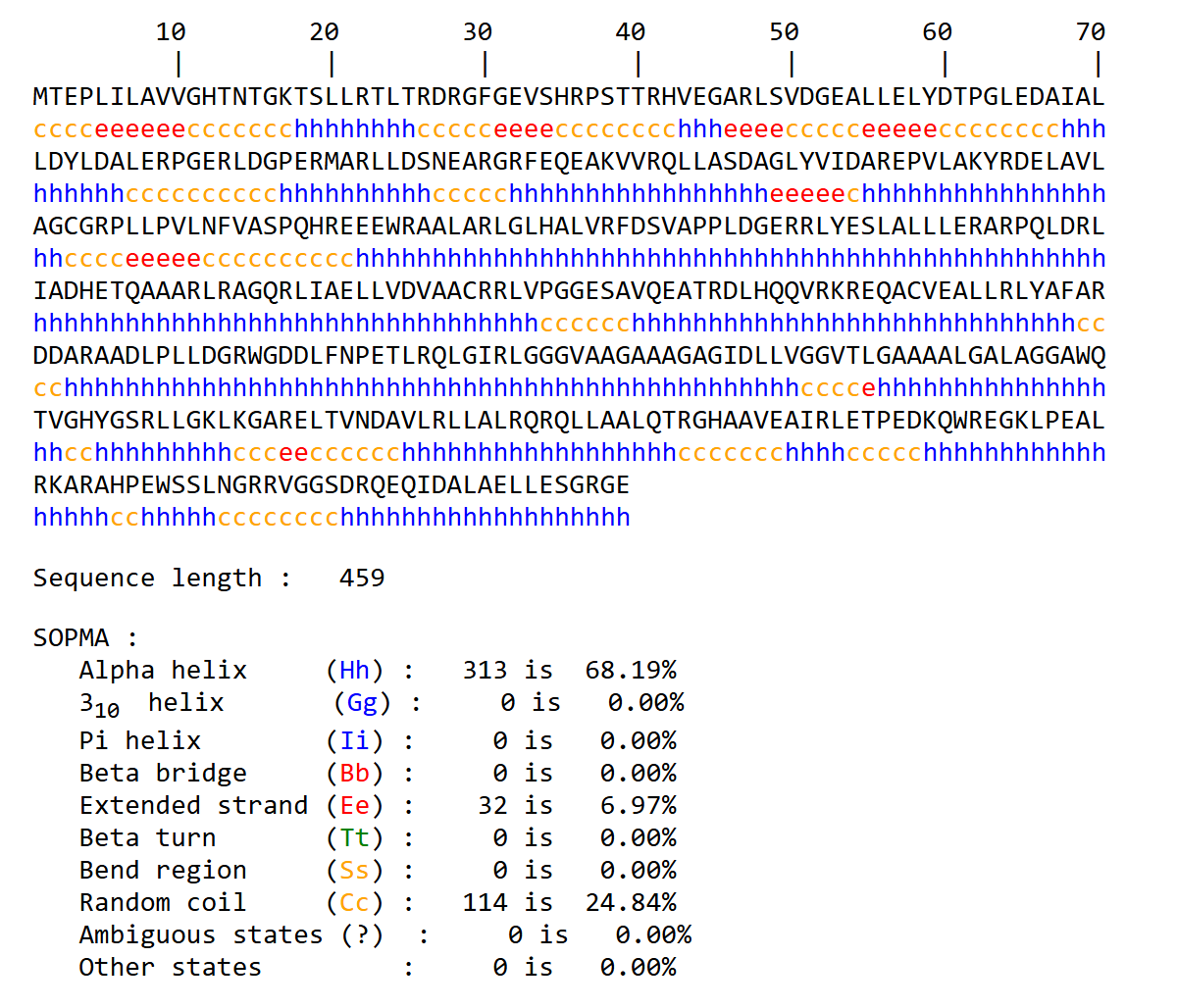
**

**HP15:**


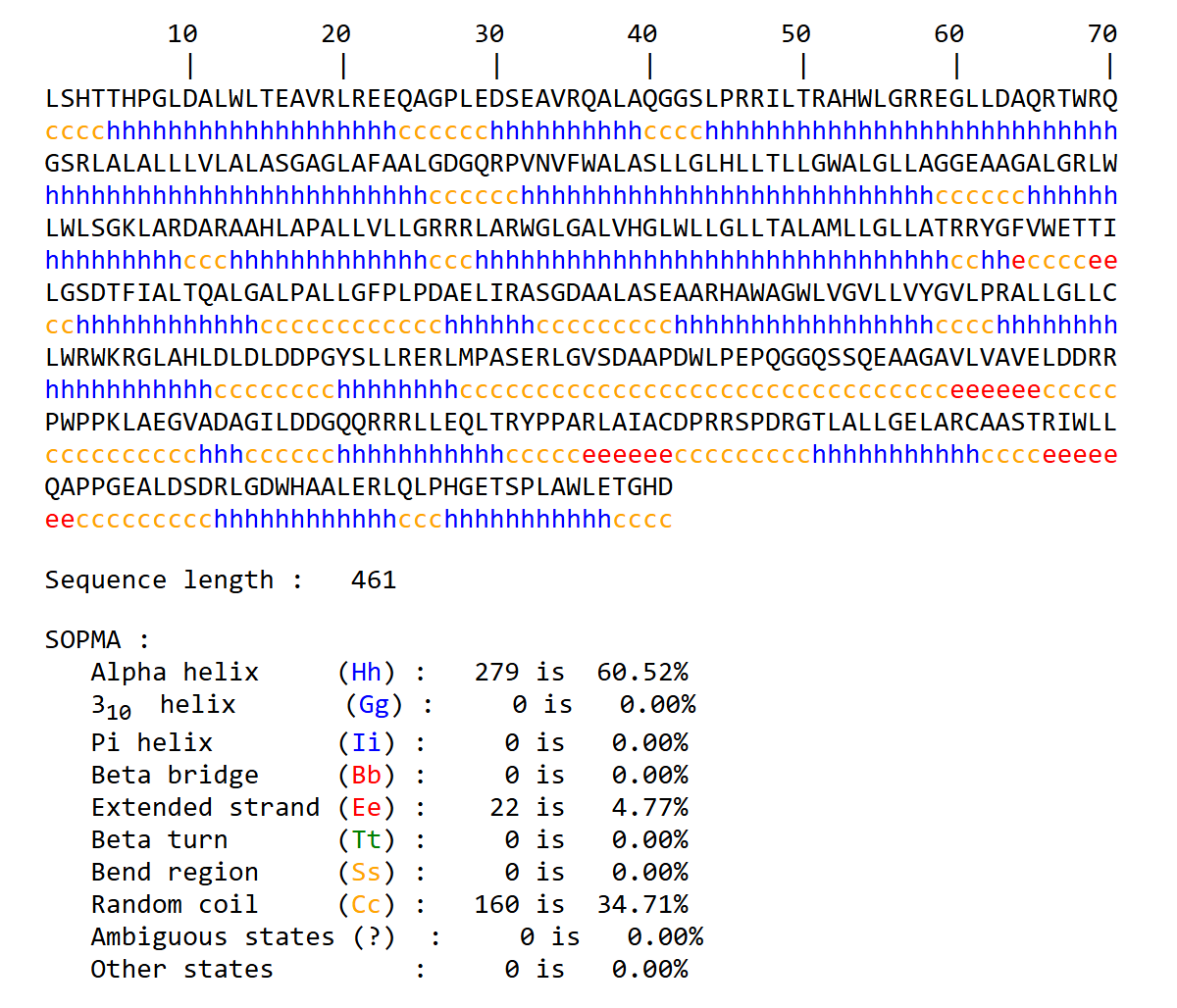

Supplement: Supplementary file 1 — Supporting Information 1 Figure S1. Secondary structures of hypothetical proteins predicted by SOPMA. [file BMRI-2026-2974616-s001.docx]
