# Supplementary material for: Structural Characterization and Functional Annotation of Hypothetical Proteins in the Multidrug‐Resistant Strains of Pseudomonas aeruginosa
Source: Biomed Res Int. 2026 Feb 2;2026:2974616. doi: 10.1155/bmri/2974616 (PMC12864544; doi:10.1155/bmri/2974616)

Supplementary Figure S2. Secondary structures of hypothetical proteins predicted by PSIPRED

HP1:


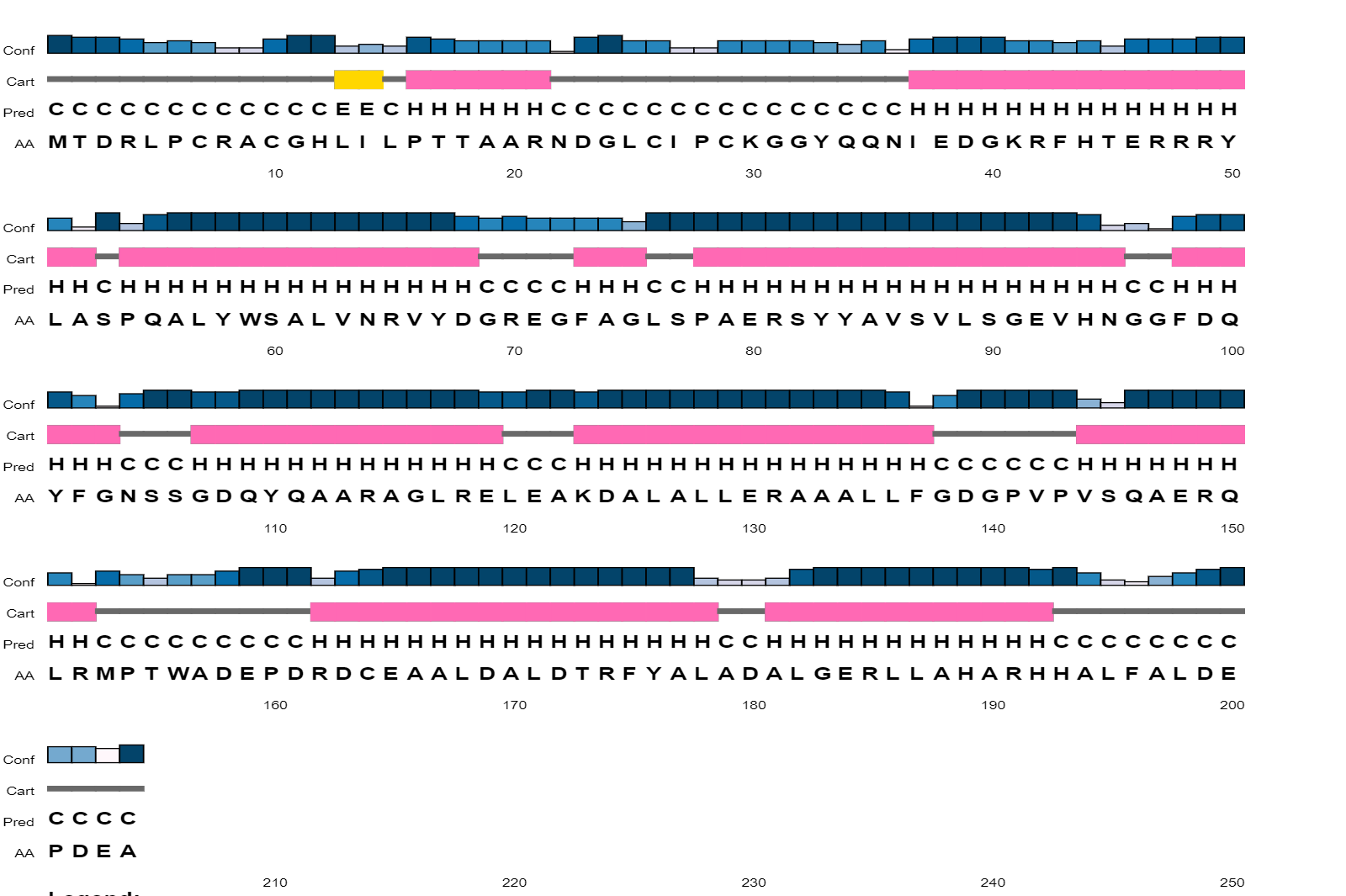


HP2:


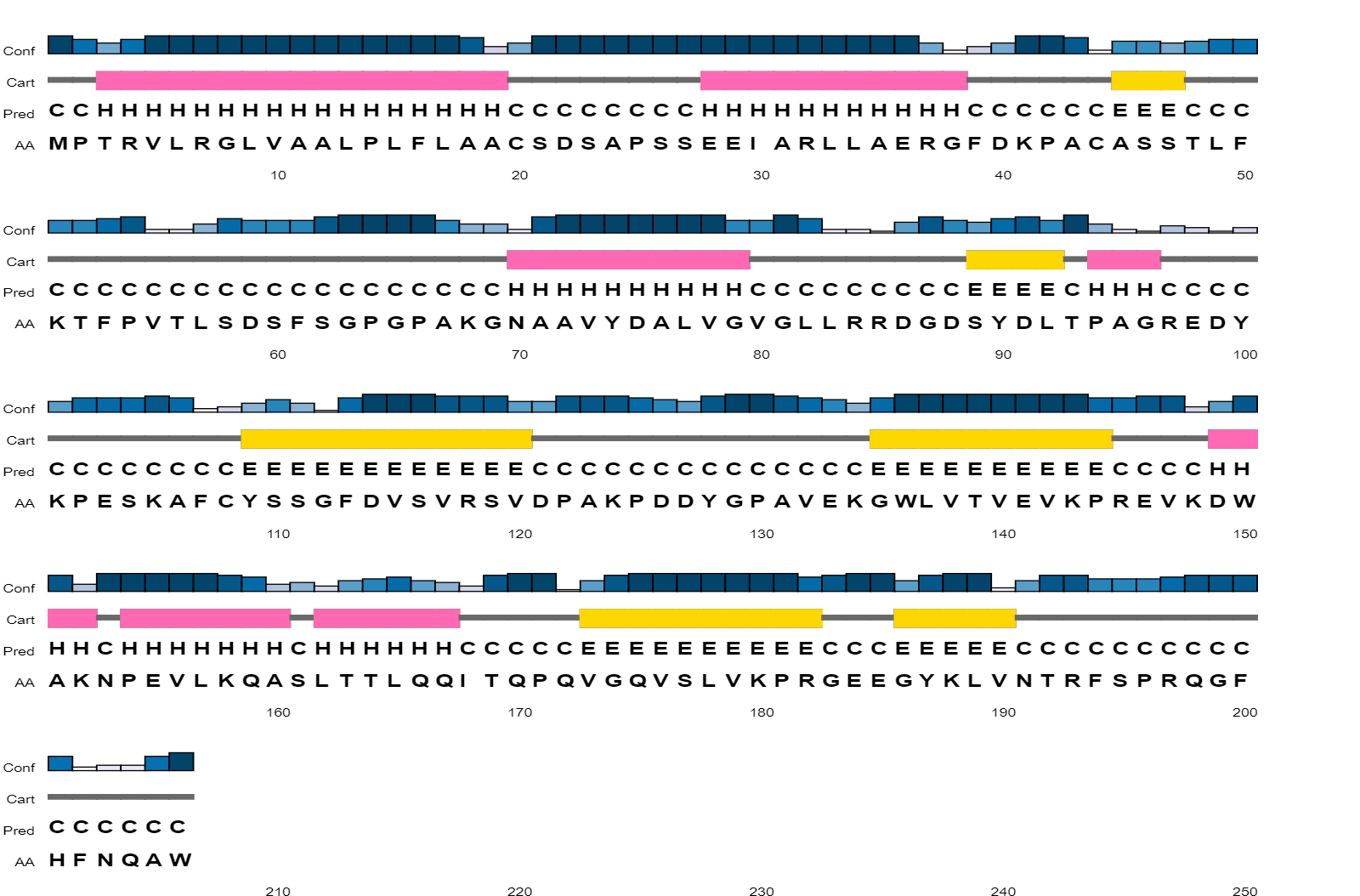


HP3:


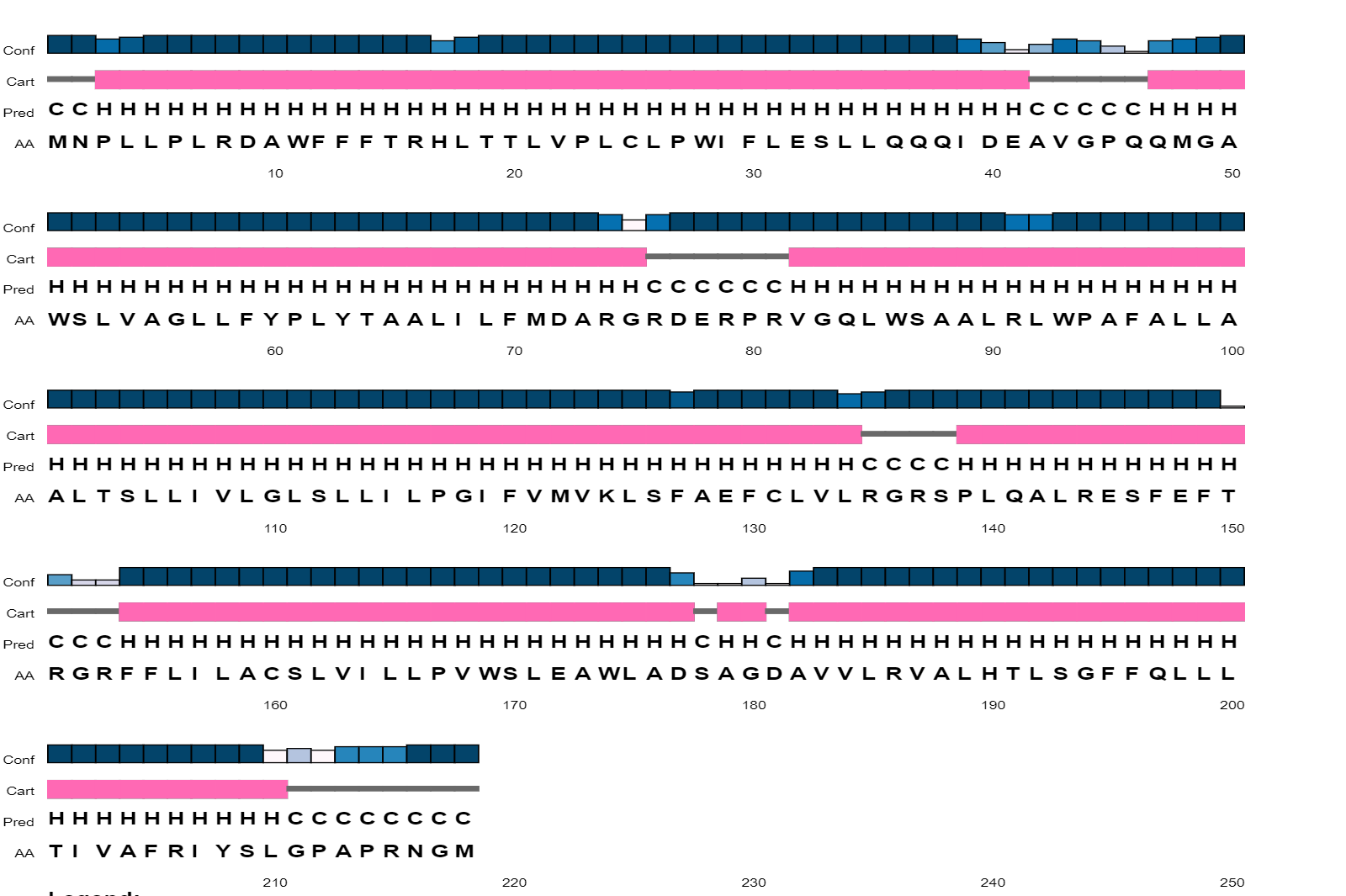


HP4:

HP5:

HP6:

HP7:

HP8:

HP9:

HP10:

HP11:

HP12:

HP13:

HP14:

HP15:

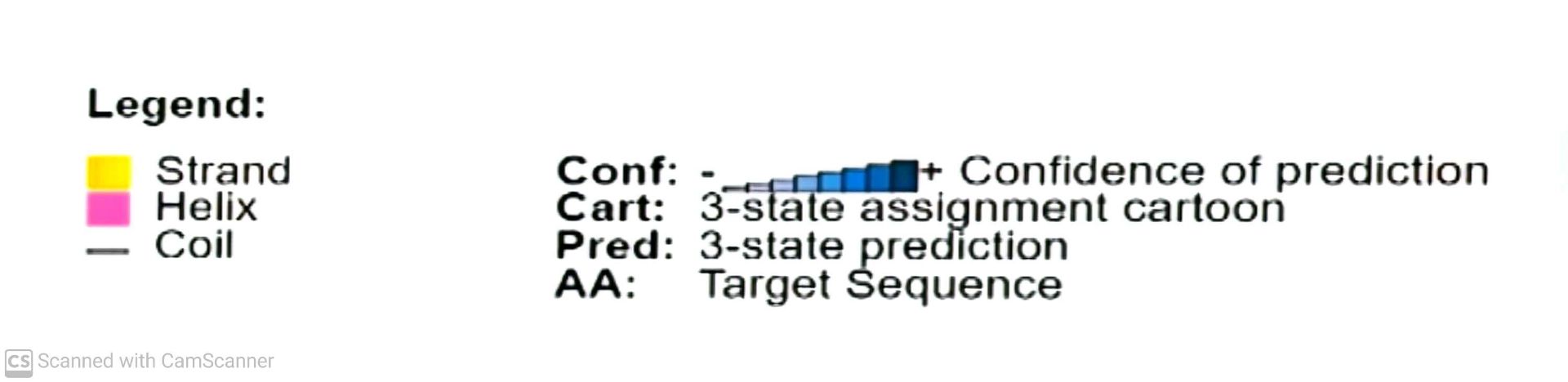

Supplement: Supplementary file 2 — Supporting Information 2 Figure S2. Secondary structures of hypothetical proteins predicted by PSIPRED. [file BMRI-2026-2974616-s002.docx]
