# Supplementary material for: Structural Characterization and Functional Annotation of Hypothetical Proteins in the Multidrug‐Resistant Strains of Pseudomonas aeruginosa
Source: Biomed Res Int. 2026 Feb 2;2026:2974616. doi: 10.1155/bmri/2974616 (PMC12864544; doi:10.1155/bmri/2974616)
Supplement: Supplementary file 3 — Supporting Information 3 Table S1. BLASTP analysis results of hypothetical proteins. [file BMRI-2026-2974616-s003.docx]

**Supplementary Table S1. BLASTP analysis results of hypothetical proteins**

| Accession | Per.Ident | E-value | Description | Protein |
| --- | --- | --- | --- | --- |
| SSU20174.1 | 100% | 5.00e^-146^ | Uncharacterised protein [Acinetobacter baumannii] | HP1 |
| MCL3028383.1 | 98.53% | 3.00e^-143^ | DMP19 family protein [Klebsiella pneumoniae] |  |
| AAT49932.1 | 98.04% | 4.00e^-143^ | PA0571 [synthetic construct] |  |

| Accession | Per.Ident | E-value | Description | Protein |
| --- | --- | --- | --- | --- |
| MCL3027736.1 | 100% | 6.00e^-148^ | hypothetical protein [Klebsiella pneumoniae] | HP2 |
| AAT49889.1 | 100% | 8.00e^-148^ | PA0126 [synthetic construct] |  |
| SST11030.1 | 100% | 2.00e^-147^ | Uncharacterised protein [Acinetobacter baumannii] |  |
| ECA4546812.1 | 99.51% | 4.00e^-147^ | hypothetical protein [Salmonella enterica subsp. enterica serovar Typhimurium] |  |

| Accession | Per.Ident | E-value | Description | Protein |
| --- | --- | --- | --- | --- |
| SCX97014.1 | 100% | 4.00e^-149^ | Uncharacterised protein [Acinetobacter baumannii] | HP3 |
| SAI98113.1 | 99.54% | 2.00e^-148^ | Uncharacterised protein family (UPF0259) [Enterobacter cloacae] |  |
| SST09068.1 | 99.54% | 3.00e^-148^ | Uncharacterised protein family (UPF0259) [Acinetobacter baumannii] |  |
| AAT49773.1 | 99.54% | 4.00e^-148^ | PA3955 [synthetic construct] |  |

| Accession | Per.Ident | E-value | Description | Protein |
| --- | --- | --- | --- | --- |
| SCY84920.1 | 100% | 5.00e^-168^ | Uncharacterised protein [Acinetobacter baumannii] | HP4 |
| AAT51682.1 | 100% | 7.00e^-168^ | PA0663 [synthetic construct] |  |
| SAJ28647.1 | 99.58% | 3.00e^-167^ | Uncharacterised protein [Enterobacter cloacae] |  |

| Accession | Per.Ident | E-value | Description | Protein |
| --- | --- | --- | --- | --- |
| SCZ09011.1 | 100% | 8.00e^-173^ | Uncharacterised protein [Acinetobacter baumannii] | HP5 |
| SAJ24629.1 | 99.6% | 1.00e^-172^ | Uncharacterized protein conserved in bacteria [Enterobacter cloacae] |  |
| SST08415.1 | 99.6% | 2.00e^-172^ | Uncharacterized protein conserved in bacteria [Acinetobacter baumannii] |  |

| Accession | Per.Ident | E-value | Description | Protein |
| --- | --- | --- | --- | --- |
| SST11141.1 | 100% | 0.0 | Uncharacterised protein [Acinetobacter baumannii] | HP6 |
| SSU01960.1 | 100% | 0.0 | Uncharacterised protein [Acinetobacter baumannii] |  |
| SCY43122.1 | 99.64% | 0.0 | Uncharacterised protein [Acinetobacter baumannii] |  |

| Accession | Per.Ident | E-value | Description | Protein |
| --- | --- | --- | --- | --- |
| SST09536.1 | 100% | 0.0 | Uncharacterised protein [Acinetobacter baumannii] | HP7 |
| SAJ32927.1 | 99.34% | 0.0 | Uncharacterised protein [Enterobacter cloacae] |  |
| SCZ12104.1 | 99.67% | 0.0 | Uncharacterised protein [Acinetobacter baumannii] |  |

| Accession | Per.Ident | E-value | Description | Protein |
| --- | --- | --- | --- | --- |
| UXX42322.1 | 100% | 0.0 | phenazine specific methyltransferase [Shuttle vector pUCP24-phzMS] | HP8 |
| AAT51474.1 | 100% | 0.0 | PA4209 [synthetic construct] |  |
| SST08793.1 | 99.7% | 0.0 | Multifunctional cyclase-dehydratase-3-O-methyl transferase tcmN [Acinetobacter baumannii] |  |

| Accession | Per.Ident | E-value | Description | Protein |
| --- | --- | --- | --- | --- |
| SCZ09364.1 | 99.71% | 0.0 | Uncharacterised protein [Acinetobacter baumannii] | HP9 |

| Accession | Per.Ident | E-value | Description | Protein |
| --- | --- | --- | --- | --- |
| SCY82520.1 | 100% | 0.0 | Uncharacterised protein [Acinetobacter baumannii] | HP10 |

| Accession | Per.Ident | E-value | Description | Protein |
| --- | --- | --- | --- | --- |
| SST08543.1 | 100% | 0.0 | Uncharacterised protein [Acinetobacter baumannii] | HP11 |
| SAJ01607.1 | 100% | 0.0 | Uncharacterised protein [Enterobacter cloacae] |  |

| Accession | Per.Ident | E-value | Description | Protein |
| --- | --- | --- | --- | --- |
| SCY99384.1 | 100% | 0.0 | Porin D precursor [Acinetobacter baumannii] | HP12 |
| SAJ29342.1 | 99.77% | 0.0 | outer membrane porin [Enterobacter cloacae] |  |

| Accession | Per.Ident | E-value | Description | Protein |
| --- | --- | --- | --- | --- |
| SAI98396.1 | 100% | 0.0 | Protein of uncharacterised function (DUF1329) [Enterobacter cloacae] | HP13 |

| Accession | Per.Ident | E-value | Description | Protein |
| --- | --- | --- | --- | --- |
| SST08455.1 | 100 | 0.0 | Predicted GTPase [Acinetobacter baumannii] | HP14 |
| SAJ24755.1 | 99.78 | 0.0 | Predicted GTPase [Enterobacter cloacae] |  |

| Accession | Per.Ident | E-value | Description | Protein |
| --- | --- | --- | --- | --- |
| SCZ09329.1 | 99.78% | 0.0 | putative membrane protein [Acinetobacter baumannii] | HP15 |
