# Supplementary material for: Structural Characterization and Functional Annotation of Hypothetical Proteins in the Multidrug‐Resistant Strains of Pseudomonas aeruginosa
Source: Biomed Res Int. 2026 Feb 2;2026:2974616. doi: 10.1155/bmri/2974616 (PMC12864544; doi:10.1155/bmri/2974616)
Supplement: Supplementary file 4 — Supporting Information 4 Table S2. T Cell epitopes identified in hypothetical proteins. [file BMRI-2026-2974616-s004.docx]

**Supplementary Table S2. T Cell Epitopes Identified in Hypothetical Proteins**

| HP1 | | | | |
| --- | --- | --- | --- | --- |
| allele | position | length | peptide | score |
| HLA-B*58:01 | 51-59 | 9 | LASPQALYW | 0.997657 |
| HLA-B*57:01 | 51-59 | 9 | LASPQALYW | 0.995031 |
| HLA-B*40:01 | 118-126 | 9 | RELEAKDAL | 0.989937 |
| HLA-B*08:01 | 187-195 | 9 | LAHARHHAL | 0.9727 |
| HLA-B*58:01 | 50-59 | 10 | YASPQALYW | 0.969665 |
| HLA-B*57:01 | 50-59 | 10 | YASPQALYW | 0.959102 |
| HLA-B*53:01 | 51-59 | 9 | LASPQALYW | 0.946177 |
| HLA-B*40:01 | 120-128 | 9 | LEAKDALAL | 0.936436 |
| HLA-A*32:01 | 65-73 | 9 | RVYDGREGF | 0.929557 |
| HLA-B*15:01 | 50-58 | 9 | YLASPQALY | 0.928923 |
| HLA-B*15:01 | 75-83 | 9 | GLSPAERSY | 0.905609 |
| HLA-A*26:01 | 92-101 | 10 | EVHGGFDQY | 0.901471 |
| HLA-A*24:02 | 49-57 | 9 | RYLASPQAL | 0.891034 |
| HLA-A*68:01 | 165-174 | 10 | EAALDALDTR | 0.883227 |
| HLA-A*02:01 | 135-144 | 10 | LLFGDGPVPV | 0.878469 |
| HLA-A*01:01 | 50-58 | 9 | YLASPQALY | 0.864834 |
| HLA-A*02:01 | 127-135 | 9 | ALLERAAAL | 0.857867 |
| HLA-B*15:01 | 65-73 | 9 | RVYDGREGF | 0.850448 |
| HLA-B*44:02 | 147-156 | 10 | AERQLRMPTW | 0.8492 |
| HLA-A*01:01 | 167-176 | 10 | ALDALDTRFY | 0.843874 |

| HP2 | | | | |
| --- | --- | --- | --- | --- |
| allele | position | length | peptide | score |
| HLA-B*35:01 | 53-61 | 9 | FPVTLSDSF | 0.991907 |
| HLA-A*02:06 | 72-80 | 9 | AVYDALVGV | 0.988454 |
| HLA-A*02:03 | 72-80 | 9 | AVYDALVGV | 0.966249 |
| HLA-A*68:01 | 29-37 | 9 | EIARLLAER | 0.965732 |
| HLA-A*02:01 | 72-80 | 9 | AVYDALVGV | 0.95841 |
| HLA-A*68:01 | 88-97 | 10 | DSYDLTPAGR | 0.95166 |
| HLA-B*07:02 | 170-178 | 9 | QPQVGQVSL | 0.949656 |
| HLA-B*07:02 | 41-49 | 9 | KPACASSTL | 0.949365 |
| HLA-B*53:01 | 53-61 | 9 | FPVTLSDSF | 0.937688 |
| HLA-B*57:01 | 45-53 | 9 | ASSTLFKTF | 0.934352 |
| HLA-B*07:02 | 180-189 | 10 | KPRGEEGYKL | 0.923613 |
| HLA-A*01:01 | 119-128 | 10 | SVDPAKPDDY | 0.904206 |
| HLA-B*58:01 | 45-53 | 9 | ASSTLFKTF | 0.864214 |
| HLA-A*24:02 | 193-202 | 10 | RFSPRQGFHF | 0.862946 |
| HLA-B*15:01 | 82-90 | 9 | LLRRDGDSY | 0.85536 |
| HLA-A*30:01 | 116-124 | 9 | SVRSVDPAK | 0.85525 |
| HLA-B*57:01 | 198-206 | 9 | QGFHFNQAW | 0.846749 |
| HLA-A*68:01 | 59-68 | 10 | DSFSGPGPAK | 0.844196 |
| HLA-A*68:02 | 72-80 | 9 | AVYDALVGV | 0.83685 |
| HLA-A*02:06 | 71-80 | 10 | AAVYDALVGV | 0.812756 |

| HP3 | | | | |
| --- | --- | --- | --- | --- |
| allele | position | length | peptide | score |
| HLA-A*68:01 | 145-153 | 9 | ESFEFTRGR | 0.981042 |
| HLA-A*68:01 | 177-186 | 10 | DSAGDAVVLR | 0.948789 |
| HLA-B*53:01 | 166-174 | 9 | LPVWSLEAW | 0.921736 |
| HLA-A*24:02 | 62-70 | 9 | LYTAALILF | 0.920495 |
| HLA-A*23:01 | 62-70 | 9 | LYTAALILF | 0.902692 |
| HLA-B*57:01 | 19-28 | 10 | TTLVPLCLPW | 0.897154 |
| HLA-A*68:01 | 178-186 | 9 | SAGDAVVLR | 0.88954 |
| HLA-B*44:03 | 77-86 | 10 | DERPRVGQLW | 0.887237 |
| HLA-A*02:01 | 97-105 | 9 | ALLAALTSL | 0.875139 |
| HLA-B*44:02 | 77-86 | 10 | DERPRVGQLW | 0.867725 |
| HLA-A*02:01 | 115-123 | 9 | ILPGIFVMV | 0.857898 |
| HLA-B*51:01 | 60-68 | 9 | YPLYTAALI | 0.851212 |
| HLA-B*53:01 | 5-13 | 9 | LPLRDAWFF | 0.825571 |
| HLA-A*02:01 | 91-99 | 9 | RLWPAFALL | 0.821258 |
| HLA-A*02:06 | 115-123 | 9 | ILPGIFVMV | 0.816597 |
| HLA-A*03:01 | 206-215 | 10 | RIYSLGPAPR | 0.812736 |
| HLA-A*31:01 | 206-215 | 10 | RIYSLGPAPR | 0.809417 |
| HLA-A*02:03 | 115-123 | 9 | ILPGIFVMV | 0.794835 |
| HLA-A*33:01 | 145-153 | 9 | ESFEFTRGR | 0.786068 |
| HLA-B*08:01 | 202-210 | 9 | IVAFRIYSL | 0.784009 |

| HP4 | | | | |
| --- | --- | --- | --- | --- |
| allele | position | length | peptide | score |
| HLA-B*44:03 | 204-212 | 9 | AELELPKNF | 0.996148 |
| HLA-B*44:02 | 204-212 | 9 | AELELPKNF | 0.992726 |
| HLA-A*01:01 | 131-140 | 10 | GTDDPQRFRY | 0.986928 |
| HLA-B*07:02 | 114-122 | 9 | APASKADAL | 0.977259 |
| HLA-B*57:01 | 226-234 | 9 | QSKPLQRRF | 0.945747 |
| HLA-A*03:01 | 161-169 | 9 | RISGKLARK | 0.945508 |
| HLA-B*07:02 | 179-187 | 9 | SPELGKTGL | 0.93016 |
| HLA-A*02:01 | 215-223 | 9 | KLIDVRAEL | 0.927339 |
| HLA-B*15:01 | 101-109 | 9 | KQQQDIAFY | 0.920816 |
| HLA-A*31:01 | 141-149 | 9 | KVMLSRLGR | 0.903216 |
| HLA-B*15:01 | 165-173 | 9 | KLARKDATY | 0.02 |
| HLA-A*03:01 | 93-101 | 9 | KLLEDQVFK | 0.03 |
| HLA-A*02:03 | 215-223 | 9 | KLIDVRAEL | 0.03 |
| HLA-B*51:01 | 208-216 | 9 | LPKNFIPKL | 0.02 |
| HLA-A*03:01 | 157-165 | 9 | RLKVRISGK | 0.04 |
| HLA-A*30:02 | 101-109 | 9 | KQQQDIAFY | 0.01 |
| HLA-B*07:02 | 197-206 | 10 | IPEGGRFAEL | 0.07 |
| HLA-A*02:06 | 215-223 | 9 | KLIDVRAEL | 0.06 |
| HLA-B*40:01 | 221-230 | 10 | AELNGQSKPL | 0.1 |
| HLA-B*40:01 | 58-67 | 10 | GEQKAELERL | 0.1 |

| HP5 | | | | |
| --- | --- | --- | --- | --- |
| allele | position | length | peptide | score |
| HLA-B*44:03 | 222-230 | 9 | AESPAGRSY | 0.996431 |
| HLA-B*44:02 | 222-230 | 9 | AESPAGRSY | 0.995286 |
| HLA-A*01:01 | 210-218 | 9 | LSDAELTQY | 0.994364 |
| HLA-B*40:01 | 146-154 | 9 | REAGAEVSL | 0.993335 |
| HLA-B*44:03 | 222-231 | 10 | AESPAGRSYY | 0.98693 |
| HLA-B*44:02 | 222-231 | 10 | AESPAGRSYY | 0.984392 |
| HLA-A*01:01 | 17-25 | 9 | LADPYQRLY | 0.977345 |
| HLA-A*02:03 | 134-142 | 9 | ALVKRLAQV | 0.972315 |
| HLA-A*68:01 | 104-112 | 9 | VAAEVLATR | 0.960565 |
| HLA-A*01:01 | 196-205 | 10 | GADLENTLLY | 0.953207 |
| HLA-A*02:03 | 16-24 | 9 | ALADPYQRL | 0.9467 |
| HLA-B*40:01 | 145-154 | 10 | AREAGAEVSL | 0.946016 |
| HLA-A*01:01 | 209-218 | 10 | DLSDAELTQY | 0.932765 |
| HLA-A*02:06 | 48-56 | 9 | YQDKLPPAL | 0.929613 |
| HLA-B*07:02 | 69-78 | 10 | APAEVDQRAL | 0.928025 |
| HLA-A*02:01 | 166-174 | 9 | QMLPGLPGL | 0.926709 |
| HLA-B*44:03 | 213-221 | 9 | AELTQYVNF | 0.926665 |
| HLA-B*15:01 | 48-57 | 10 | YQDKLPPALY | 0.924626 |
| HLA-A*68:02 | 151-159 | 9 | EVSLALAGV | 0.924493 |
| HLA-A*02:06 | 166-174 | 9 | QMLPGLPGL | 0.90544 |

| HP6 | | | | |
| --- | --- | --- | --- | --- |
| allele | position | length | peptide | score |
| HLA-B*57:01 | 179-187 | 9 | TTYMLNLTW | 0.989882 |
| HLA-B*58:01 | 179-187 | 9 | TTYMLNLTW | 0.984404 |
| HLA-B*40:01 | 221-229 | 9 | AETLVRTAL | 0.981385 |
| HLA-A*01:01 | 82-91 | 10 | GTDHGGSEVY | 0.970771 |
| HLA-B*35:01 | 172-181 | 10 | HPDHTFDTTY | 0.966133 |
| HLA-A*01:01 | 151-159 | 9 | VLDLSLLYY | 0.963368 |
| HLA-A*68:02 | 175-183 | 9 | HTFDTTYML | 0.958016 |
| HLA-A*24:02 | 46-54 | 9 | KFAKRIYSF | 0.949564 |
| HLA-A*24:02 | 157-165 | 9 | LYYREKNHF | 0.943464 |
| HLA-A*23:01 | 46-54 | 9 | KFAKRIYSF | 0.937307 |
| HLA-A*68:01 | 218-226 | 9 | DTAAETLVR | 0.936745 |
| HLA-A*23:01 | 157-165 | 9 | LYYREKNHF | 0.934743 |
| HLA-A*24:02 | 61-69 | 9 | RYGSNFFNL | 0.929295 |
| HLA-A*01:01 | 26-34 | 9 | MSNSVGFRY | 0.920128 |
| HLA-A*03:01 | 137-145 | 9 | ALVFGPTLK | 0.03 |
| HLA-B*08:01 | 132-140 | 9 | SAKKRALVF | 0.02 |
| HLA-A*68:01 | 118-126 | 9 | FTLGFDASR | 0.1 |
| HLA-A*24:02 | 90-98 | 9 | VYAVYRHQL | 0.04 |
| HLA-B*15:01 | 96-104 | 9 | HQLYASRVF | 0.03 |
| HLA-A*01:01 | 54-62 | 9 | FTHADGYRY | 0.05 |

| HP7 | | | | |
| --- | --- | --- | --- | --- |
| allele | position | length | peptide | score |
| HLA-B*44:03 | 100-108 | 9 | AEAELRPFW | 0.996989 |
| HLA-B*44:02 | 100-108 | 9 | AEAELRPFW | 0.996234 |
| HLA-B*40:01 | 218-227 | 10 | SEYGDGGYQL | 0.988317 |
| HLA-B*58:01 | 156-165 | 9 | LAQTEPPQW | 0.98644 |
| HLA-B*57:01 | 156-164 | 9 | LAQTEPPQW | 0.975254 |
| HLA-B*44:03 | 276-284 | 9 | EEYLGGYNF | 0.961816 |
| HLA-B*07:02 | 260-268 | 9 | RPVQGVPEM | 0.96127 |
| HLA-B*35:01 | 35-43 | 9 | WPLDPDTGY | 0.958742 |
| HLA-B*58:01 | 14-22 | 9 | HAGEANHGW | 0.95154 |
| HLA-B*35:01 | 260-268 | 9 | RPVQGVPEM | 0.938965 |
| HLA-B*57:01 | 223-231 | 9 | GGYQLPHYW | 0.922378 |
| HLA-A*02:01 | 51-59 | 9 | LLLPEDYRV | 0.921844 |
| HLA-B*35:01 | 43-51 | 9 | YPLMHGFTL | 0.920084 |
| HLA-B*57:01 | 14-22 | 9 | HAGEANHGW | 0.913846 |
| HLA-B*35:01 | 212-220 | 9 | NAGVVPSEY | 0.911104 |
| HLA-B*44:02 | 276-284 | 9 | EEYLGGYNF | 0.909895 |
| HLA-B*58:01 | 169-177 | 9 | SAASFVPDW | 0.90851 |
| HLA-B*44:03 | 16-24 | 9 | GEANHGWCF | 0.900184 |
| HLA-B*58:01 | 223-231 | 9 | GGYQLPHYW | 0.06 |
| HLA-B*07:02 | 251-259 | 9 | LPNHIGGTM | 0.04 |

| HP8 | | | | |
| --- | --- | --- | --- | --- |
| allele | position | length | peptide | score |
| HLA-B*53:01 | 279-287 | 9 | EPSPMSVLW | 0.991383 |
| HLA-B*44:03 | 278-287 | 10 | SEPSPMSVLW | 0.977614 |
| HLA-B*15:01 | 149-157 | 9 | AMKASNLAF | 0.975989 |
| HLA-B*44:02 | 278-287 | 10 | SEPSPMSVLW | 0.972812 |
| HLA-B*53:01 | 278-287 | 10 | SEPSPMSVLW | 0.923149 |
| HLA-B*40:01 | 278-286 | 9 | SEPSPMSVL | 0.916547 |
| HLA-A*68:01 | 325-333 | 9 | ETRMIVAAR | 0.910761 |
| HLA-A*33:01 | 325-333 | 9 | ETRMIVAAR | 0.906802 |
| HLA-B*44:03 | 158-166 | 9 | HEIPRLLDF | 0.904841 |
| HLA-B*57:01 | 93-101 | 9 | GSFRDMVLF | 0.896762 |
| HLA-B*51:01 | 322-330 | 9 | LPMETRMIV | 0.895903 |
| HLA-B*07:02 | 138-147 | 10 | RCPDAGRRFL | 0.882045 |
| HLA-A*32:01 | 60-68 | 9 | RLMRLLVAF | 0.872603 |
| HLA-B*15:01 | 87-95 | 9 | LLRDVEGSF | 0.860045 |
| HLA-A*68:01 | 81-89 | 9 | NTPTSHLLR | 0.850835 |
| HLA-B*07:02 | 188-197 | 10 | AEPSARGVML | 0.841396 |
| HLA-B*44:02 | 158-166 | 9 | HEIPRLLDF | 0.835664 |
| HLA-B*07:02 | 322-330 | 9 | LPMETRMIV | 0.830658 |
| HLA-B*58:01 | 93-101 | 9 | GSFRDMVLF | 0.824704 |
| HLA-B*44:03 | 18-26 | 9 | GEWKSRCVY | 0.809105 |

| HP9 | | | | |
| --- | --- | --- | --- | --- |
| allele | position | length | peptide | score |
| HLA-B*58:01 | 274-282 | 9 | AAAGPSFLW | 0.99364 |
| HLA-B*57:01 | 274-282 | 9 | AAAGPSFLW | 0.990021 |
| HLA-A*03:01 | 295-303 | 9 | ITYAQVLLK | 0.980745 |
| HLA-B*35:01 | 179-187 | 9 | DAASGALVY | 0.976149 |
| HLA-B*07:02 | 208-216 | 9 | LPMSALVSL | 0.973516 |
| HLA-A*02:03 | 321-329 | 9 | VLATGTTQV | 0.968032 |
| HLA-A*11:01 | 295-303 | 9 | ITYAQVLLK | 0.964995 |
| HLA-A*03:01 | 133-142 | 10 | ILAYDGTMLK | 0.962828 |
| HLA-A*26:01 | 305-313 | 9 | ETPDQVLTF | 0.95989 |
| HLA-B*07:02 | 262-270 | 9 | APGQPSPVF | 0.952311 |
| HLA-A*68:01 | 179-188 | 10 | DAASGALVYR | 0.949999 |
| HLA-B*07:02 | 121-129 | 9 | SPRQLANLF | 0.94964 |
| HLA-A*32:01 | 289-297 | 9 | KIFEILITY | 0.948837 |
| HLA-A*11:01 | 134-142 | 9 | LAYDGTMLK | 0.92541 |
| HLA-B*57:01 | 158-167 | 10 | RVRPLGVGPW | 0.925235 |
| HLA-B*07:02 | 105-114 | 10 | RPQDMHFQVM | 0.916341 |
| HLA-B*07:02 | 188-197 | 10 | RPDQLRVIGL | 0.898358 |
| HLA-B*35:01 | 208-216 | 9 | LPMSALVSL | 0.897803 |
| HLA-A*68:01 | 311-319 | 9 | LTFNLQDYR | 0.891739 |

| HP10 | | | | |
| --- | --- | --- | --- | --- |
| allele | position | length | peptide | score |
| HLA-B*40:01 | 218-226 | 9 | GELPPVLAL | 0.996418 |
| HLA-B*40:01 | 216-224 | 9 | REGELPPVL | 0.989851 |
| HLA-B*58:01 | 321-329 | 9 | RAVLKEEQW | 0.984716 |
| HLA-B*57:01 | 321-329 | 9 | RAVLKEEQW | 0.982451 |
| HLA-B*57:01 | 129-138 | 10 | LSLAEQVPAW | 0.977241 |
| HLA-B*40:01 | 297-305 | 9 | SEQLNQVLL | 0.976473 |
| HLA-B*57:01 | 175-184 | 10 | KVLDDDWVQW | 0.975712 |
| HLA-A*31:01 | 149-157 | 9 | RVLERWRYR | 0.971448 |
| HLA-A*31:01 | 275-283 | 9 | RVLQSLAQR | 0.969058 |
| HLA-B*58:01 | 175-184 | 10 | KVLDDDWVQW | 0.96809 |
| HLA-A*68:01 | 344-352 | 9 | DALEPIIRR | 0.957782 |
| HLA-B*44:02 | 201-210 | 10 | VEDDEGRVKW | 0.956998 |
| HLA-B*44:03 | 201-210 | 10 | VEDDEGRVKW | 0.951086 |
| HLA-B*58:01 | 129-138 | 10 | LSLAEQVPAW | 0.94406 |
| HLA-B*40:01 | 248-256 | 9 | LENVAPEIL | 0.936844 |
| HLA-A*33:01 | 332-340 | 9 | EVRDWLAAR | 0.934553 |
| HLA-B*07:02 | 286-295 | 10 | APVRRGRRPL | 0.924853 |
| HLA-A*68:01 | 219-227 | 9 | ELPPVLALR | 0.917621 |
| HLA-A*31:01 | 305-315 | 10 | RLFDDRPWPR | 0.91429 |
| HLA-A*68:01 | 332-340 | 9 | EVRDWLAAR | 0.90831 |

| HP11 | | | | |
| --- | --- | --- | --- | --- |
| allele | position | length | peptide | score |
| HLA-B*58:01 | 63-71 | 9 | VTAEELVAW | 0.989122 |
| HLA-B*57:01 | 63-71 | 9 | VTAEELVAW | 0.98661 |
| HLA-B*07:02 | 257-266 | 10 | LPRPPRAKRL | 0.978262 |
| HLA-B*07:02 | 277-286 | 10 | APMRQFSIEL | 0.965115 |
| HLA-B*57:01 | 212-220 | 9 | CTDLIHHYW | 0.959329 |
| HLA-A*30:02 | 280-288 | 9 | RQFSIELLY | 0.957259 |
| HLA-B*40:01 | 326-334 | 9 | IEDWLNHSL | 0.956095 |
| HLA-B*15:01 | 280-288 | 9 | RQFSIELLY | 0.952362 |
| HLA-A*24:02 | 218-226 | 9 | HYWSRKQLF | 0.947272 |
| HLA-A*23:01 | 218-226 | 9 | HYWSRKQLF | 0.940174 |
| HLA-A*68:01 | 102-111 | 10 | DTFFHSSPLR | 0.935674 |
| HLA-B*58:01 | 212-220 | 9 | CTDLIHHYW | 0.934913 |
| HLA-A*03:01 | 256-264 | 9 | RLPRPPRAK | 0.934 |
| HLA-B*15:01 | 22-30 | 9 | ALFSIASAF | 0.930558 |
| HLA-B*57:01 | 211-220 | 10 | GCTDLIHHYW | 0.924327 |
| HLA-B*44:03 | 131-139 | 9 | GEHRNFPLY | 0.922174 |
| HLA-A*33:01 | 214-222 | 9 | DLIHHYWSR | 0.92211 |
| HLA-B*08:01 | 78-86 | 9 | HFRAKHVAL | 0.921478 |
| HLA-B*07:02 | 166-174 | 9 | LPREEAKIL | 0.920231 |
| HLA-B*15:01 | 10-18 | 9 | YLVYGRQTY | 0.915586 |

| HP12 | | | | |
| --- | --- | --- | --- | --- |
| allele | position | length | peptide | score |
| HLA-B*58:01 | 231-239 | 9 | YAAELQNVW | 0.988126 |
| HLA-B*44:03 | 233-242 | 10 | AELQNVWKQY | 0.985137 |
| HLA-B*40:01 | 250-258 | 9 | YEIDDNWSL | 0.980381 |
| HLA-B*53:01 | 231-239 | 9 | YAAELQNVW | 0.977364 |
| HLA-B*44:02 | 233-242 | 10 | AELQNVWKQY | 0.974194 |
| HLA-B*58:01 | 248-256 | 9 | YTYEIDDNW | 0.970799 |
| HLA-B*57:01 | 231-239 | 9 | YAAELQNVW | 0.966582 |
| HLA-A*30:02 | 257-265 | 9 | SLNPGAHYY | 0.957364 |
| HLA-B*57:01 | 248-256 | 9 | YTYEIDDNW | 0.956625 |
| HLA-B*44:03 | 333-341 | 9 | NEKSWKLQY | 0.950961 |
| HLA-A*68:01 | 294-302 | 9 | HTVTAVLQK | 0.948021 |
| HLA-A*11:01 | 294-302 | 9 | HTVTAVLQK | 0.941364 |
| HLA-A*01:01 | 425-433 | 9 | AVDNDIDEY | 0.938147 |
| HLA-A*01:01 | 368-376 | 9 | RVDPDSPGY | 0.937423 |
| HLA-B*15:01 | 181-189 | 9 | GQVSFTKYY | 0.928975 |
| HLA-A*68:02 | 295-303 | 9 | TVTAVLQKV | 0.922709 |
| HLA-A*02:06 | 69-77 | 9 | FQSGYTPGV | 0.922254 |
| HLA-A*68:02 | 281-289 | 9 | NTYSLHFAV | 0.919316 |
| HLA-B*15:01 | 257-265 | 9 | SLNPGAHYY | 0.918378 |
| HLA-B*57:01 | 230-239 | 10 | LYAAELQNVW | 0.908638 |

| HP13 | | | | |
| --- | --- | --- | --- | --- |
| allele | position | length | peptide | score |
| HLA-A*68:01 | 111-119 | 9 | DTYKIPVYK | 0.992448 |
| HLA-A*23:01 | 199-207 | 9 | VYFQDAFTF | 0.991644 |
| HLA-A*24:02 | 199-207 | 9 | VYFQDAFTF | 0.988863 |
| HLA-A*26:01 | 167-176 | 10 | EVIWNHITRY | 0.986191 |
| HLA-B*44:03 | 392-400 | 9 | AEAHAEQYY | 0.984349 |
| HLA-B*07:02 | 232-241 | 10 | APSRLAGNVL | 0.98269 |
| HLA-B*57:01 | 339-348 | 10 | LTRYELHRVW | 0.981393 |
| HLA-A*68:01 | 167-175 | 9 | EVIWNHITR | 0.980697 |
| HLA-B*07:02 | 215-223 | 9 | NPNKPSNVL | 0.978088 |
| HLA-A*68:01 | 410-419 | 10 | ETLYDLLSGR | 0.971516 |
| HLA-B*44:02 | 392-400 | 9 | AEAHAEQYY | 0.965679 |
| HLA-A*24:02 | 76-84 | 9 | PYASEQPLF | 0.95675 |
| HLA-A*26:01 | 309-317 | 9 | EIYIPYNSY | 0.950692 |
| HLA-A*01:01 | 317-326 | 10 | YKLDDPKIKY | 0.949508 |
| HLA-B*35:01 | 109-118 | 10 | YPDTYKIPVY | 0.947449 |
| HLA-A*01:01 | 318-326 | 9 | KLDDPKIKY | 0.940807 |
| HLA-A*68:01 | 441-450 | 10 | ESDYTPAALR | 0.940697 |
| HLA-B*40:01 | 440-449 | 10 | SESDYTPAAL | 0.925923 |
| HLA-A*01:01 | 285-293 | 9 | RTSDNFDMY | 0.923424 |
| HLA-B*07:02 | 214-223 | 10 | YNPNKPSNVL | 0.914952 |

| HP14 | | | | |
| --- | --- | --- | --- | --- |
| allele | position | length | peptide | score |
| HLA-B*44:03 | 105-113 | 9 | QEAKVVRQL | 0.977879 |
| HLA-B*40:01 | 160-168 | 9 | REEEWRAAL | 0.97455 |
| HLA-B*44:02 | 105-113 | 9 | QEAKVVRQL | 0.971189 |
| HLA-B*40:01 | 105-113 | 9 | QEAKVVRQL | 0.97081 |
| HLA-A*68:01 | 32-41 | 10 | EVSHRPSTTR | 0.965745 |
| HLA-B*40:01 | 412-420 | 9 | REGKLPEAL | 0.958342 |
| HLA-B*40:01 | 214-222 | 9 | HETQAAARL | 0.957767 |
| HLA-A*68:01 | 247-255 | 9 | ESAVQEATR | 0.937811 |
| HLA-A*68:01 | 181-190 | 10 | SVAPPLDGER | 0.936203 |
| HLA-A*02:01 | 69-77 | 9 | ALLDYLDAL | 0.935669 |
| HLA-A*02:01 | 191-199 | 9 | RLYESLALL | 0.934228 |
| HLA-A*68:01 | 368-377 | 10 | ELTVNDAVLR | 0.929624 |
| HLA-A*68:01 | 194-202 | 9 | ESLALLLER | 0.926872 |
| HLA-B*08:01 | 373-381 | 9 | DAVLRLLAL | 0.925888 |
| HLA-A*02:03 | 191-199 | 9 | RLYESLALL | 0.924806 |
| HLA-B*44:03 | 403-411 | 9 | LETPEDKQW | 0.920524 |
| HLA-A*68:01 | 215-223 | 9 | ETQAAARLR | 0.920192 |
| HLA-A*68:01 | 298-306 | 9 | DLFNPETLR | 0.919532 |
| HLA-B*40:01 | 264-272 | 9 | REQACVEAL | 0.913271 |
| HLA-B*40:01 | 443-452 | 10 | QEQIDALAEL | 0.895766 |

| HP15 | | | | |
| --- | --- | --- | --- | --- |
| allele | position | length | peptide | score |
| HLA-B*57:01 | 134-142 | 9 | GALGRLWLW | 0.990721 |
| HLA-B*57:01 | 198-206 | 9 | ATRRYGFVW | 0.984118 |
| HLA-B*57:01 | 4-13 | 10 | TTHPGLDALW | 0.983721 |
| HLA-B*07:02 | 396-404 | 9 | SPDRGTLAL | 0.971302 |
| HLA-B*57:01 | 132-140 | 9 | AAGALGRLW | 0.969824 |
| HLA-B*58:01 | 134-142 | 9 | GALGRLWLW | 0.966483 |
| HLA-B*58:01 | 4-13 | 10 | TTHPGLDALW | 0.964717 |
| HLA-B*07:02 | 445-453 | 9 | LPHGETSPL | 0.958506 |
| HLA-B*57:01 | 133-142 | 10 | AGALGRLWLW | 0.953842 |
| HLA-B*57:01 | 273-282 | 10 | RALLGLLCLW | 0.94883 |
| HLA-B*57:01 | 248-257 | 10 | LASEAARHAW | 0.940274 |
| HLA-B*58:01 | 132-140 | 9 | AAGALGRLW | 0.939334 |
| HLA-B*44:03 | 344-352 | 9 | VELDDRRPW | 0.93637 |
| HLA-B*07:02 | 396-405 | 10 | SPDRGTLALL | 0.931628 |
| HLA-B*44:02 | 343-352 | 10 | AVELDDRRPW | 0.928925 |
| HLA-B*44:02 | 344-352 | 9 | VELDDRRPW | 0.923686 |
| HLA-B*44:03 | 343-352 | 10 | AVELDDRRPW | 0.922375 |
| HLA-B*07:02 | 271-279 | 9 | LPRALLGLL | 0.915353 |
| HLA-B*57:01 | 410-418 | 9 | RCAASTRIW | 0.912983 |
| HLA-B*58:01 | 248-257 | 10 | LASEAARHAW | 0.904853 |

**MHCII**

| HP1 | | | | |
| --- | --- | --- | --- | --- |
| allele | position | length | peptide | Percentile rank |
| HLA-DRB5*01:01 | 105-119 | 15 | SSGDQYQAARAGLRE | 0.01 |
| HLA-DRB5*01:01 | 108-121 | 14 | DQYQAARAGLRELE | 0.01 |
| HLA-DRB5*01:01 | 106-119 | 14 | SGDQYQAARAGLRE | 0.01 |
| HLA-DRB5*01:01 | 106-120 | 15 | SGDQYQAARAGLREL | 0.01 |
| HLA-DRB5*01:01 | 107-120 | 14 | GDQYQAARAGLREL | 0.01 |
| HLA-DRB5*01:01 | 107-121 | 15 | GDQYQAARAGLRELE | 0.01 |
| HLA-DRB5*01:01 | 108-122 | 15 | DQYQAARAGLRELEA | 0.04 |
| HLA-DRB5*01:01 | 70-83 | 14 | REGFAGLSPAERSY | 0.06 |
| HLA-DRB5*01:01 | 70-84 | 15 | REGFAGLSPAERSYY | 0.06 |
| HLA-DRB5*01:01 | 69-83 | 15 | GREGFAGLSPAERSY | 0.08 |
| HLA-DRB5*01:01 | 69-82 | 14 | GREGFAGLSPAERS | 0.11 |
| HLA-DRB1*04:01 | 172-185 | 14 | DTRFYALADALGER | 0.11 |
| HLA-DRB5*01:01 | 68-82 | 15 | DGREGFAGLSPAERS | 0.13 |
| HLA-DRB1*04:01 | 171-185 | 15 | LDTRFYALADALGER | 0.13 |
| HLA-DRB5*01:01 | 71-84 | 14 | EGFAGLSPAERSYY | 0.14 |
| HLA-DRB5*01:01 | 105-118 | 14 | SSGDQYQAARAGLR | 0.14 |
| HLA-DRB1*04:05 | 172-185 | 14 | DTRFYALADALGER | 0.14 |
| HLA-DRB1*04:01 | 80-93 | 14 | ERSYYAVSVLSGEV | 0.17 |
| HLA-DRB1*04:01 | 172-186 | 15 | DTRFYALADALGERL | 0.18 |
| HLA-DRB1*04:01 | 80-94 | 15 | ERSYYAVSVLSGEVH | 0.18 |

| HP2 | | | | |
| --- | --- | --- | --- | --- |
| allele | position | length | peptide | Percentile rank |
| HLA-DQA1*04:01/DQB1*04:02 | 184-198 | 15 | EEGYKLVNTRFSPRQ | 0.08 |
| HLA-DQA1*04:01/DQB1*04:02 | 184-197 | 14 | EEGYKLVNTRFSPR | 0.11 |
| HLA-DRB1*04:01 | 184-197 | 14 | EEGYKLVNTRFSPR | 0.15 |
| HLA-DRB1*08:02 | 184-198 | 15 | EEGYKLVNTRFSPRQ | 0.17 |
| HLA-DRB1*04:01 | 184-198 | 15 | EEGYKLVNTRFSPRQ | 0.18 |
| HLA-DRB1*08:02 | 184-197 | 14 | EEGYKLVNTRFSPR | 0.2 |
| HLA-DQA1*04:01/DQB1*04:02 | 183-197 | 15 | GEEGYKLVNTRFSPR | 0.22 |
| HLA-DQA1*04:01/DQB1*04:02 | 185-198 | 14 | EGYKLVNTRFSPRQ | 0.26 |
| HLA-DRB1*04:01 | 183-197 | 15 | GEEGYKLVNTRFSPR | 0.26 |
| HLA-DQA1*04:01/DQB1*04:02 | 183-196 | 14 | GEEGYKLVNTRFSP | 0.29 |
| HLA-DRB1*01:01 | 71-85 | 15 | AAVYDALVGVGLLRR | 0.29 |
| HLA-DRB1*08:02 | 183-197 | 15 | GEEGYKLVNTRFSPR | 0.34 |
| HLA-DRB1*04:01 | 183-196 | 14 | GEEGYKLVNTRFSP | 0.38 |
| HLA-DRB1*04:01 | 185-198 | 14 | EGYKLVNTRFSPRQ | 0.39 |
| HLA-DRB1*04:01 | 47-60 | 14 | STLFKTFPVTLSDS | 0.43 |
| HLA-DRB1*08:02 | 185-198 | 14 | EGYKLVNTRFSPRQ | 0.47 |
| HLA-DRB1*01:01 | 72-85 | 14 | AVYDALVGVGLLRR | 0.51 |
| HLA-DQA1*04:01/DQB1*04:02 | 182-196 | 15 | RGEEGYKLVNTRFSP | 0.52 |
| HLA-DRB1*08:02 | 183-196 | 14 | GEEGYKLVNTRFSP | 0.54 |
| HLA-DRB4*01:01 | 112-126 | 15 | GFDVSVRSVDPAKPD | 0.56 |

| HP3 | | | | |
| --- | --- | --- | --- | --- |
| allele | position | length | peptide | Percentile rank |
| HLA-DRB4*01:01 | 30-43 | 14 | FLESLLQQQIDEAV | 0.01 |
| HLA-DRB4*01:01 | 31-44 | 14 | LESLLQQQIDEAVG | 0.01 |
| HLA-DRB4*01:01 | 31-45 | 15 | LESLLQQQIDEAVGP | 0.01 |
| HLA-DRB4*01:01 | 32-45 | 14 | ESLLQQQIDEAVGP | 0.01 |
| HLA-DRB4*01:01 | 32-46 | 15 | ESLLQQQIDEAVGPQ | 0.03 |
| HLA-DRB4*01:01 | 30-44 | 15 | FLESLLQQQIDEAVG | 0.04 |
| HLA-DRB1*15:01 | 87-100 | 14 | SAALRLWPAFALLA | 0.1 |
| HLA-DRB4*01:01 | 29-43 | 15 | IFLESLLQQQIDEAV | 0.11 |
| HLA-DRB1*15:01 | 87-101 | 15 | SAALRLWPAFALLAA | 0.13 |
| HLA-DRB4*01:01 | 33-46 | 14 | SLLQQQIDEAVGPQ | 0.14 |
| HLA-DRB5*01:01 | 65-78 | 14 | AALILFMDARGRDE | 0.15 |
| HLA-DRB5*01:01 | 65-79 | 15 | AALILFMDARGRDER | 0.22 |
| HLA-DRB1*15:01 | 88-101 | 14 | AALRLWPAFALLAA | 0.22 |
| HLA-DRB1*15:01 | 86-100 | 15 | WSAALRLWPAFALLA | 0.23 |
| HLA-DRB4*01:01 | 29-42 | 14 | IFLESLLQQQIDEA | 0.27 |
| HLA-DRB5*01:01 | 66-79 | 14 | ALILFMDARGRDER | 0.29 |
| HLA-DRB1*15:01 | 86-99 | 14 | WSAALRLWPAFALL | 0.31 |
| HLA-DRB5*01:01 | 64-78 | 15 | TAALILFMDARGRDE | 0.35 |
| HLA-DRB5*01:01 | 204-217 | 14 | AFRIYSLGPAPRNG | 0.39 |
| HLA-DRB4*01:01 | 33-47 | 15 | SLLQQQIDEAVGPQQ | 0.41 |

| HP4 | | | | |
| --- | --- | --- | --- | --- |
| allele | position | length | peptide | Percentile rank |
| HLA-DRB1*15:01 | 104-118 | 15 | QDIAFYKGVVAPASK | 0.01 |
| HLA-DRB1*15:01 | 101-115 | 15 | KQQQDIAFYKGVVAP | 0.01 |
| HLA-DRB1*15:01 | 102-115 | 14 | QQQDIAFYKGVVAP | 0.01 |
| HLA-DRB1*15:01 | 104-117 | 14 | QDIAFYKGVVAPAS | 0.01 |
| HLA-DRB1*15:01 | 102-116 | 15 | QQQDIAFYKGVVAPA | 0.01 |
| HLA-DRB1*15:01 | 103-116 | 14 | QQDIAFYKGVVAPA | 0.01 |
| HLA-DRB1*15:01 | 103-117 | 15 | QQDIAFYKGVVAPAS | 0.01 |
| HLA-DRB1*12:01 | 89-103 | 15 | RQTIKLLEDQVFKQQ | 0.02 |
| HLA-DQA1*04:01/DQB1*04:02 | 121-135 | 15 | ALEIRAFEVQGTDDP | 0.03 |
| HLA-DQA1*04:01/DQB1*04:02 | 120-134 | 15 | DALEIRAFEVQGTDD | 0.04 |
| HLA-DRB1*13:02 | 157-171 | 15 | RLKVRISGKLARKDA | 0.05 |
| HLA-DRB1*12:01 | 88-102 | 15 | SRQTIKLLEDQVFKQ | 0.05 |
| HLA-DQA1*04:01/DQB1*04:02 | 122-135 | 14 | LEIRAFEVQGTDDP | 0.06 |
| HLA-DQA1*04:01/DQB1*04:02 | 121-134 | 14 | ALEIRAFEVQGTDD | 0.06 |
| HLA-DQA1*01:02/DQB1*06:02 | 35-49 | 15 | GRWSTGADAARSEAD | 0.06 |
| HLA-DRB1*12:01 | 89-102 | 14 | RQTIKLLEDQVFKQ | 0.06 |
| HLA-DQA1*01:02/DQB1*06:02 | 35-48 | 14 | GRWSTGADAARSEA | 0.07 |
| HLA-DRB1*13:02 | 157-170 | 14 | RLKVRISGKLARKD | 0.08 |
| HLA-DQA1*05:01/DQB1*03:01 | 105-118 | 14 | DIAFYKGVVAPASK | 0.09 |
| HLA-DQA1*01:01/DQB1*05:01 | 103-116 | 14 | QQDIAFYKGVVAPA | 0.1 |

| HP5 | | | | |
| --- | --- | --- | --- | --- |
| allele | position | length | NSNRRFAPAEVDQRA | Percentile rank |
| HLA-DPA1*02:01/DPB1*14:01 | 63-77 | 15 | NSNRRFAPAEVDQR | 0.02 |
| HLA-DPA1*02:01/DPB1*14:01 | 63-76 | 14 | DNSNRRFAPAEVDQR | 0.03 |
| HLA-DPA1*02:01/DPB1*14:01 | 62-76 | 15 | RSYYQAALAALKAG | 0.04 |
| HLA-DRB5*01:01 | 228-241 | 14 | SNRRFAPAEVDQRA | 0.04 |
| HLA-DPA1*02:01/DPB1*14:01 | 64-77 | 14 | GRSYYQAALAALKAG | 0.05 |
| HLA-DRB5*01:01 | 227-241 | 15 | DNSNRRFAPAEVDQ | 0.08 |
| HLA-DPA1*02:01/DPB1*14:01 | 62-75 | 14 | PAREAGAEVSLALAG | 0.09 |
| HLA-DQA1*01:02/DQB1*06:02 | 144-158 | 15 | ASPLNFFESPLGRK | 0.1 |
| HLA-DRB1*15:01 | 89-102 | 14 | GRSYYQAALAALKA | 0.1 |
| HLA-DRB5*01:01 | 227-240 | 14 | VDNSNRRFAPAEVDQ | 0.11 |
| HLA-DPA1*02:01/DPB1*14:01 | 61-75 | 15 | SNRRFAPAEVDQRAL | 0.12 |
| HLA-DPA1*02:01/DPB1*14:01 | 64-78 | 15 | RSYYQAALAALKAGL | 0.12 |
| HLA-DRB5*01:01 | 228-242 | 15 | ASPLNFFESPLGRKV | 0.12 |
| HLA-DRB1*15:01 | 89-103 | 15 | SASPLNFFESPLGRK | 0.13 |
| HLA-DRB1*15:01 | 88-102 | 15 | PAREAGAEVSLALA | 0.14 |
| HLA-DQA1*01:02/DQB1*06:02 | 144-157 | 14 | PPALYQTLVDNSNRR | 0.16 |
| HLA-DRB4*01:01 | 53-67 | 15 | GRKVVAAEVLATRRD | 0.19 |
| HLA-DQA1*05:01/DQB1*03:01 | 100-114 | 15 | GRKVVAAEVLATRRD | 0.19 |
| HLA-DQA1*01:02/DQB1*06:02 | 100-114 | 15 | QAALAALKAGLAVD | 0.21 |
| HLA-DRB1*01:01 | 232-245 | 14 | NSNRRFAPAEVDQRA | 0.22 |

| HP6 | | | | |
| --- | --- | --- | --- | --- |
| allele | position | length | peptide | Percentile rank |
| HLA-DQA1*04:01/DQB1*04:02 | 246-260 | 15 | GVGYEYWHNKFGVDG | 0.13 |
| HLA-DQA1*04:01/DQB1*04:02 | 197-211 | 15 | AAKFQGFVNYVGEKG | 0.19 |
| HLA-DPA1*02:01/DPB1*01:01 | 64-78 | 15 | SNFFNLDVFLSDNRD | 0.2 |
| HLA-DPA1*01:03/DPB1*04:01 | 64-78 | 15 | SNFFNLDVFLSDNRD | 0.25 |
| HLA-DQA1*04:01/DQB1*04:02 | 245-259 | 15 | LGVGYEYWHNKFGVD | 0.27 |
| HLA-DRB1*13:02 | 87-101 | 15 | GSEVYAVYRHQLYAS | 0.29 |
| HLA-DRB1*13:02 | 88-102 | 15 | SEVYAVYRHQLYASR | 0.32 |
| HLA-DPA1*02:01/DPB1*14:01 | 47-61 | 15 | FAKRIYSFTHADGYR | 0.33 |
| HLA-DPA1*03:01/DPB1*04:02 | 64-78 | 15 | SNFFNLDVFLSDNRD | 0.39 |
| HLA-DRB1*03:01 | 69-83 | 15 | LDVFLSDNRDPRKGT | 0.39 |
| HLA-DQA1*01:01/DQB1*05:01 | 197-211 | 15 | AAKFQGFVNYVGEKG | 0.4 |
| HLA-DRB1*09:01 | 49-63 | 15 | KRIYSFTHADGYRYG | 0.41 |
| HLA-DPA1*02:01/DPB1*01:01 | 63-77 | 15 | GSNFFNLDVFLSDNR | 0.43 |
| HLA-DRB1*03:01 | 68-82 | 15 | NLDVFLSDNRDPRKG | 0.45 |
| HLA-DRB1*09:01 | 51-65 | 15 | IYSFTHADGYRYGSN | 0.48 |
| HLA-DPA1*01:03/DPB1*04:01 | 63-77 | 15 | GSNFFNLDVFLSDNR | 0.5 |
| HLA-DQA1*01:02/DQB1*06:02 | 2-16 | 15 | KKMMQGAAALIALVG | 0.5 |
| HLA-DQA1*04:01/DQB1*04:02 | 196-210 | 15 | HAAKFQGFVNYVGEK | 0.52 |
| HLA-DRB1*15:01 | 197-211 | 15 | AAKFQGFVNYVGEKG | 0.59 |
| HLA-DQA1*05:01/DQB1*03:01 | 2-16 | 15 | KKMMQGAAALIALVG | 0.6 |

| HP7 | | | | |
| --- | --- | --- | --- | --- |
| allele | position | length | peptide | Percentile rank |
| HLA-DRB1*01:01 | 179-193 | 15 | EDDYYLLRALGGRPA | 0.01 |
| HLA-DRB1*01:01 | 178-192 | 15 | AEDDYYLLRALGGRP | 0.02 |
| HLA-DRB4*01:01 | 84-98 | 15 | VDGIREMLIDPPAQP | 0.11 |
| HLA-DRB1*04:05 | 67-81 | 15 | LSFFSLAPDQNDGGP | 0.16 |
| HLA-DRB1*04:01 | 5-19 | 15 | DIEIVNDAAHAGEAN | 0.17 |
| HLA-DQA1*05:01/DQB1*03:01 | 281-295 | 15 | GYNFGGGNAQLDFRD | 0.18 |
| HLA-DRB4*01:01 | 83-97 | 15 | SVDGIREMLIDPPAQ | 0.19 |
| HLA-DRB1*04:01 | 4-18 | 15 | YDIEIVNDAAHAGEA | 0.2 |
| HLA-DQA1*01:02/DQB1*06:02 | 7-21 | 15 | EIVNDAAHAGEANHG | 0.21 |
| HLA-DRB1*01:01 | 180-194 | 15 | DDYYLLRALGGRPAA | 0.23 |
| HLA-DRB1*04:05 | 66-80 | 15 | ALSFFSLAPDQNDGG | 0.26 |
| HLA-DRB1*01:01 | 177-191 | 15 | WAEDDYYLLRALGGR | 0.27 |
| HLA-DRB4*01:01 | 85-99 | 15 | DGIREMLIDPPAQPP | 0.3 |
| HLA-DRB1*04:01 | 3-17 | 15 | AYDIEIVNDAAHAGE | 0.3 |
| HLA-DQA1*04:01/DQB1*04:02 | 179-193 | 15 | EDDYYLLRALGGRPA | 0.32 |
| HLA-DQA1*05:01/DQB1*03:01 | 8-22 | 15 | IVNDAAHAGEANHGW | 0.32 |
| HLA-DRB1*08:02 | 179-193 | 15 | EDDYYLLRALGGRPA | 0.34 |
| HLA-DQA1*01:02/DQB1*06:02 | 6-20 | 15 | IEIVNDAAHAGEANH | 0.35 |
| HLA-DRB1*03:01 | 5-19 | 15 | DIEIVNDAAHAGEAN | 0.4 |
| HLA-DRB1*09:01 | 54-68 | 15 | PEDYRVHGPEIVALS | 0.41 |

| HP8 | | | | |
| --- | --- | --- | --- | --- |
| allele | position | length | peptide | Percentile rank |
| HLA-DPA1*03:01/DPB1*04:02 | 98-112 | 15 | MVLFYGEEFHAAWTP | 0.02 |
| HLA-DPA1*02:01/DPB1*05:01 | 98-112 | 15 | MVLFYGEEFHAAWTP | 0.05 |
| HLA-DPA1*01:03/DPB1*02:01 | 163-177 | 15 | LLDFRGRSFVDVGGG | 0.07 |
| HLA-DPA1*02:01/DPB1*01:01 | 98-112 | 15 | MVLFYGEEFHAAWTP | 0.09 |
| HLA-DPA1*03:01/DPB1*04:02 | 97-111 | 15 | DMVLFYGEEFHAAWT | 0.09 |
| HLA-DPA1*01:03/DPB1*02:01 | 162-176 | 15 | RLLDFRGRSFVDVGG | 0.09 |
| HLA-DPA1*03:01/DPB1*04:02 | 99-113 | 15 | VLFYGEEFHAAWTPA | 0.1 |
| HLA-DPA1*01:03/DPB1*02:01 | 98-112 | 15 | MVLFYGEEFHAAWTP | 0.1 |
| HLA-DPA1*01:03/DPB1*02:01 | 164-178 | 15 | LDFRGRSFVDVGGGS | 0.1 |
| HLA-DPA1*01:03/DPB1*04:01 | 98-112 | 15 | MVLFYGEEFHAAWTP | 0.11 |
| HLA-DRB1*04:01 | 266-280 | 15 | GRVVVIERTISASEP | 0.13 |
| HLA-DRB1*11:01 | 265-279 | 15 | DGRVVVIERTISASE | 0.14 |
| HLA-DRB1*04:01 | 38-52 | 15 | ESGIDSDETLAAAVG | 0.15 |
| HLA-DPA1*02:01/DPB1*01:01 | 97-111 | 15 | DMVLFYGEEFHAAWT | 0.2 |
| HLA-DPA1*01:03/DPB1*02:01 | 161-175 | 15 | PRLLDFRGRSFVDVG | 0.2 |
| HLA-DRB1*04:01 | 37-51 | 15 | IESGIDSDETLAAAV | 0.2 |
| HLA-DRB1*04:01 | 265-279 | 15 | DGRVVVIERTISASE | 0.2 |
| HLA-DRB1*11:01 | 154-168 | 15 | NLAFHEIPRLLDFRG | 0.2 |
| HLA-DPA1*01:03/DPB1*04:01 | 97-111 | 15 | DMVLFYGEEFHAAWT | 0.22 |
| HLA-DPA1*03:01/DPB1*04:02 | 96-110 | 15 | RDMVLFYGEEFHAAW | 0.23 |

| HP9 | | | | |
| --- | --- | --- | --- | --- |
| HLA-DRB5*01:01 | position | length | peptide | Percentile rank |
| HLA-DRB1*15:01 | 141-155 | 15 | LKDFRMSSAAGRLEV | 0.04 |
| HLA-DRB1*15:01 | 130-144 | 15 | NEHILAYDGTMLKDF | 0.04 |
| HLA-DRB5*01:01 | 129-143 | 15 | FNEHILAYDGTMLKD | 0.04 |
| HLA-DRB1*07:01 | 140-154 | 15 | MLKDFRMSSAAGRLE | 0.05 |
| HLA-DRB5*01:01 | 38-52 | 15 | EGWYRQVPPAPVPLP | 0.08 |
| HLA-DRB1*01:01 | 142-156 | 15 | KDFRMSSAAGRLEVE | 0.12 |
| HLA-DRB1*07:01 | 108-122 | 15 | DMHFQVMEGEVLLSP | 0.14 |
| HLA-DRB1*07:01 | 36-50 | 15 | LREGWYRQVPPAPVP | 0.15 |
| HLA-DRB5*01:01 | 37-51 | 15 | REGWYRQVPPAPVPL | 0.15 |
| HLA-DQA1*05:01/DQB1*03:01 | 139-153 | 15 | TMLKDFRMSSAAGRL | 0.22 |
| HLA-DPA1*02:01/DPB1*14:01 | 14-28 | 15 | LLAASAAQAESAIDR | 0.24 |
| HLA-DRB1*01:01 | 142-156 | 15 | KDFRMSSAAGRLEVE | 0.25 |
| HLA-DRB1*09:01 | 38-52 | 15 | EGWYRQVPPAPVPLP | 0.29 |
| HLA-DRB1*15:01 | 38-52 | 15 | EGWYRQVPPAPVPLP | 0.31 |
| HLA-DRB3*01:01 | 128-142 | 15 | LFNEHILAYDGTMLK | 0.31 |
| HLA-DRB1*12:01 | 246-260 | 15 | QEAWLDDQGLHLRFS | 0.32 |
| HLA-DRB1*07:01 | 188-202 | 15 | RPDQLRVIGLPLYGA | 0.32 |
| HLA-DPA1*02:01/DPB1*14:01 | 318-332 | 15 | YRKVLATGTTQVNED | 0.32 |
| HLA-DRB1*12:01 | 141-155 | 15 | LKDFRMSSAAGRLEV | 0.33 |
| HLA-DRB5*01:01 | 30-44 | 15 | QRELALLREGWYRQV | 0.34 |

| HP10 | | | | |
| --- | --- | --- | --- | --- |
| allele | position | length | peptide | Percentile rank |
| HLA-DRB1*13:02 | 205-219 | 15 | EGRVKWWRKKAREGE | 0.13 |
| HLA-DRB1*09:01 | 273-287 | 15 | RERVLQSLAQRVDAP | 0.15 |
| HLA-DRB1*07:01 | 273-287 | 15 | RERVLQSLAQRVDAP | 0.15 |
| HLA-DRB5*01:01 | 314-328 | 15 | PRVLTRARAVLKEEQ | 0.19 |
| HLA-DRB1*01:01 | 86-100 | 15 | SPTLEALRGLRGEAR | 0.2 |
| HLA-DRB1*08:02 | 192-206 | 15 | NDALIALRRVEDDEG | 0.21 |
| HLA-DRB1*11:01 | 192-206 | 15 | NDALIALRRVEDDEG | 0.21 |
| HLA-DRB1*13:02 | 204-218 | 15 | DEGRVKWWRKKAREG | 0.23 |
| HLA-DQA1*01:02/DQB1*06:02 | 68-82 | 15 | VKSLVAASPAQASVP | 0.24 |
| HLA-DRB5*01:01 | 313-327 | 15 | WPRVLTRARAVLKEE | 0.25 |
| HLA-DQA1*01:02/DQB1*06:02 | 69-83 | 15 | KSLVAASPAQASVPP | 0.26 |
| HLA-DRB1*07:01 | 272-286 | 15 | QRERVLQSLAQRVDA | 0.26 |
| HLA-DRB4*01:01 | 195-209 | 15 | LIALRRVEDDEGRVK | 0.3 |
| HLA-DRB1*01:01 | 85-99 | 15 | DSPTLEALRGLRGEA | 0.32 |
| HLA-DPA1*03:01/DPB1*04:02 | 292-306 | 15 | RRPLDSEQLNQVLLR | 0.34 |
| HLA-DQA1*05:01/DQB1*03:01 | 68-82 | 15 | VKSLVAASPAQASVP | 0.37 |
| HLA-DRB1*11:01 | 191-205 | 15 | DNDALIALRRVEDDE | 0.4 |
| HLA-DQA1*05:01/DQB1*03:01 | 69-83 | 15 | KSLVAASPAQASVPP | 0.41 |
| HLA-DRB1*09:01 | 272-286 | 15 | QRERVLQSLAQRVDA | 0.42 |
| HLA-DQA1*04:01/DQB1*04:02 | 207-221 | 15 | RVKWWRKKAREGELP | 0.44 |

| HP11 | | | | |
| --- | --- | --- | --- | --- |
| allele | position | length | peptide | Percentile rank |
| HLA-DQA1*01:01/DQB1*05:01 | 40-54 | 15 | GLTVRVYTDDPSPYA | 0.01 |
| HLA-DRB1*09:01 | 302-316 | 15 | GPVWWEKAATNAIER | 0.02 |
| HLA-DRB1*13:02 | 74-88 | 15 | PHGYHFRAKHVALRD | 0.03 |
| HLA-DRB1*09:01 | 303-317 | 15 | PVWWEKAATNAIERY | 0.03 |
| HLA-DRB5*01:01 | 194-208 | 15 | LEEFVLAVAAHKRLE | 0.03 |
| HLA-DQA1*01:01/DQB1*05:01 | 39-53 | 15 | HGLTVRVYTDDPSPY | 0.04 |
| HLA-DRB5*01:01 | 195-209 | 15 | EEFVLAVAAHKRLEL | 0.05 |
| HLA-DRB1*09:01 | 101-115 | 15 | TDTFFHSSPLRLFDR | 0.06 |
| HLA-DRB5*01:01 | 193-207 | 15 | TLEEFVLAVAAHKRL | 0.06 |
| HLA-DRB1*13:02 | 248-262 | 15 | DDTLKVTDRLPRPPR | 0.07 |
| HLA-DRB1*13:02 | 75-89 | 15 | HGYHFRAKHVALRDA | 0.07 |
| HLA-DRB1*09:01 | 301-315 | 15 | CGPVWWEKAATNAIE | 0.08 |
| HLA-DRB5*01:01 | 192-206 | 15 | YTLEEFVLAVAAHKR | 0.08 |
| HLA-DRB1*07:01 | 101-115 | 15 | TDTFFHSSPLRLFDR | 0.11 |
| HLA-DQA1*01:01/DQB1*05:01 | 38-52 | 15 | AHGLTVRVYTDDPSP | 0.14 |
| HLA-DQA1*05:01/DQB1*02:01 | 41-55 | 15 | LTVRVYTDDPSPYAG | 0.14 |
| HLA-DRB1*15:01 | 311-325 | 15 | TNAIERYPQEPIAEQ | 0.14 |
| HLA-DRB1*07:01 | 100-114 | 15 | DTDTFFHSSPLRLFD | 0.15 |
| HLA-DRB1*07:01 | 335-349 | 15 | LRNLLGSRANEIRQH | 0.15 |
| HLA-DRB5*01:01 | 50-64 | 15 | PSPYAGLPVQVREVT | 0.16 |

| HP12 | | | | |
| --- | --- | --- | --- | --- |
| allele | position | length | peptide | Percentile rank |
| HLA-DQA1*01:01/DQB1*05:01 | 429-443 | 15 | DIDEYRVIVDYPIDV | 0.01 |
| HLA-DRB3*02:02 | 141-155 | 15 | LGDQFLSNPVVAGGE | 0.01 |
| HLA-DRB3*02:02 | 142-156 | 15 | GDQFLSNPVVAGGES | 0.01 |
| HLA-DRB1*04:05 | 420-434 | 15 | TGGYSAVDNDIDEYR | 0.01 |
| HLA-DQA1*01:01/DQB1*05:01 | 430-444 | 15 | IDEYRVIVDYPIDVF | 0.03 |
| HLA-DQA1*01:01/DQB1*05:01 | 428-442 | 15 | NDIDEYRVIVDYPID | 0.04 |
| HLA-DRB3*02:02 | 140-154 | 15 | KLGDQFLSNPVVAGG | 0.05 |
| HLA-DRB1*04:05 | 419-433 | 15 | GTGGYSAVDNDIDEY | 0.05 |
| HLA-DQA1*04:01/DQB1*04:02 | 373-387 | 15 | SPGYGGWYSADGKNA | 0.06 |
| HLA-DQA1*04:01/DQB1*04:02 | 259-273 | 15 | NPGAHYYKTVDSGDS | 0.06 |
| HLA-DRB3*02:02 | 143-157 | 15 | DQFLSNPVVAGGESR | 0.07 |
| HLA-DRB5*01:01 | 395-409 | 15 | DLQYVVQGGPAKDLS | 0.08 |
| HLA-DRB5*01:01 | 185-199 | 15 | FTKYYNQSGHRRLGS | 0.08 |
| HLA-DRB3*01:01 | 315-329 | 15 | GDSIFLDNSQQYSDF | 0.08 |
| HLA-DRB3*01:01 | 314-328 | 15 | QGDSIFLDNSQQYSD | 0.08 |
| HLA-DQA1*03:01/DQB1*03:02 | 420-434 | 15 | TGGYSAVDNDIDEYR | 0.1 |
| HLA-DRB5*01:01 | 184-198 | 15 | SFTKYYNQSGHRRLG | 0.1 |
| HLA-DRB5*01:01 | 100-114 | 15 | GTSILPITAPSKEGY | 0.1 |
| HLA-DRB3*01:01 | 247-261 | 15 | DYTYEIDDNWSLNPG | 0.1 |
| HLA-DRB5*01:01 | 99-113 | 15 | GGTSILPITAPSKEG | 0.11 |

| HP13 | | | | |
| --- | --- | --- | --- | --- |
| allele | position | length | peptide | Percentile rank |
| HLA-DQA1*01:02/DQB1*06:02 | 121-135 | 15 | HRSATVPAAVQEAAK | 0.01 |
| HLA-DRB1*09:01 | 150-164 | 15 | LENFDTANPFPIPQN | 0.01 |
| HLA-DQA1*01:02/DQB1*06:02 | 122-136 | 15 | RSATVPAAVQEAAKR | 0.01 |
| HLA-DRB1*04:01 | 364-378 | 15 | KRDFYIDEDTWQAAE | 0.01 |
| HLA-DPA1*02:01/DPB1*14:01 | 118-132 | 15 | YKTHRSATVPAAVQE | 0.02 |
| HLA-DPA1*02:01/DPB1*14:01 | 119-133 | 15 | KTHRSATVPAAVQEA | 0.02 |
| HLA-DRB1*09:01 | 432-446 | 15 | AYDFNYSASESDYTP | 0.03 |
| HLA-DRB1*09:01 | 149-163 | 15 | GLENFDTANPFPIPQ | 0.03 |
| HLA-DQA1*03:01/DQB1*03:02 | 371-385 | 15 | EDTWQAAEIDHYDGR | 0.05 |
| HLA-DQA1*01:02/DQB1*06:02 | 123-137 | 15 | SATVPAAVQEAAKRN | 0.06 |
| HLA-DRB1*15:01 | 169-183 | 15 | IWNHITRYRGGSVRR | 0.06 |
| HLA-DRB1*04:01 | 363-377 | 15 | AKRDFYIDEDTWQAA | 0.06 |
| HLA-DRB1*09:01 | 385-399 | 15 | RGTLWRVAEAHAEQY | 0.07 |
| HLA-DRB1*09:01 | 151-165 | 15 | ENFDTANPFPIPQNG | 0.07 |
| HLA-DRB3*02:02 | 210-224 | 15 | NLKDYNPNKPSNVLF | 0.07 |
| HLA-DRB3*02:02 | 211-225 | 15 | LKDYNPNKPSNVLFY | 0.07 |
| HLA-DRB1*15:01 | 170-184 | 15 | WNHITRYRGGSVRRL | 0.08 |
| HLA-DRB1*11:01 | 166-180 | 15 | LEVIWNHITRYRGGS | 0.08 |
| HLA-DRB1*11:01 | 167-181 | 15 | EVIWNHITRYRGGSV | 0.08 |
| HLA-DQA1*03:01/DQB1*03:02 | 370-384 | 15 | DEDTWQAAEIDHYDG | 0.09 |

| HP14 | | | | |
| --- | --- | --- | --- | --- |
| allele | position | length | peptide | Percentile rank |
| HLA-DQA1*01:02/DQB1*06:02 | 214-228 | 15 | HETQAAARLRAGQRL | 0.01 |
| HLA-DQA1*01:02/DQB1*06:02 | 213-227 | 15 | DHETQAAARLRAGQR | 0.01 |
| HLA-DQA1*05:01/DQB1*03:01 | 313-327 | 15 | GGGVAAGAAAGAGID | 0.01 |
| HLA-DRB1*01:01 | 426-440 | 15 | HPEWSSLNGRRVGGS | 0.02 |
| HLA-DRB1*09:01 | 149-163 | 15 | PVLNFVASPQHREEE | 0.03 |
| HLA-DRB1*09:01 | 150-164 | 15 | VLNFVASPQHREEEW | 0.04 |
| HLA-DQA1*05:01/DQB1*03:01 | 314-328 | 15 | GGVAAGAAAGAGIDL | 0.04 |
| HLA-DQA1*05:01/DQB1*03:01 | 311-325 | 15 | RLGGGVAAGAAAGAG | 0.05 |
| HLA-DQA1*05:01/DQB1*03:01 | 312-326 | 15 | LGGGVAAGAAAGAGI | 0.05 |
| HLA-DRB1*01:01 | 425-439 | 15 | AHPEWSSLNGRRVGG | 0.05 |
| HLA-DRB1*09:01 | 148-162 | 15 | LPVLNFVASPQHREE | 0.07 |
| HLA-DQA1*01:02/DQB1*06:02 | 212-226 | 15 | ADHETQAAARLRAGQ | 0.08 |
| HLA-DRB1*15:01 | 53-67 | 15 | EALLELYDTPGLEDA | 0.08 |
| HLA-DQA1*05:01/DQB1*03:01 | 309-323 | 15 | GIRLGGGVAAGAAAG | 0.11 |
| HLA-DPA1*02:01/DPB1*14:01 | 280-294 | 15 | RDDARAADLPLLDGR | 0.12 |
| HLA-DQA1*05:01/DQB1*03:01 | 310-324 | 15 | IRLGGGVAAGAAAGA | 0.14 |
| HLA-DQA1*05:01/DQB1*03:01 | 308-322 | 15 | LGIRLGGGVAAGAAA | 0.16 |
| HLA-DRB1*15:01 | 127-141 | 15 | EPVLAKYRDELAVLA | 0.16 |
| HLA-DQA1*04:01/DQB1*04:02 | 161-175 | 15 | EEEWRAALARLGLHA | 0.19 |
| HLA-DRB1*15:01 | 52-66 | 15 | GEALLELYDTPGLED | 0.19 |

| 15 | | | | |
| --- | --- | --- | --- | --- |
| allele | position | length | peptide | Percentile rank |
| HLA-DQA1*05:01/DQB1*03:01 | 124-138 | 15 | LGLLAGGEAAGALGR | 0.12 |
| HLA-DRB1*03:01 | 144-158 | 15 | SGKLARDARAAHLAP | 0.12 |
| HLA-DQA1*01:02/DQB1*06:02 | 148-162 | 15 | ARDARAAHLAPALLV | 0.19 |
| HLA-DQA1*05:01/DQB1*03:01 | 123-137 | 15 | ALGLLAGGEAAGALG | 0.19 |
| HLA-DRB1*04:01 | 201-215 | 15 | RYGFVWETTILGSDT | 0.19 |
| HLA-DRB1*04:01 | 200-214 | 15 | RRYGFVWETTILGSD | 0.21 |
| HLA-DRB1*03:01 | 361-375 | 15 | ADAGILDDGQQRRRL | 0.21 |
| HLA-DQA1*01:02/DQB1*06:02 | 147-161 | 15 | LARDARAAHLAPALL | 0.22 |
| HLA-DQA1*05:01/DQB1*03:01 | 125-139 | 15 | GLLAGGEAAGALGRL | 0.22 |
| HLA-DRB1*01:01 | 137-151 | 15 | GRLWLWLSGKLARDA | 0.23 |
| HLA-DRB4*01:01 | 285-299 | 15 | KRGLAHLDLDLDDPG | 0.24 |
| HLA-DRB1*03:01 | 362-376 | 15 | DAGILDDGQQRRRLL | 0.24 |
| HLA-DRB1*03:01 | 143-157 | 15 | LSGKLARDARAAHLA | 0.28 |
| HLA-DRB1*03:01 | 360-374 | 15 | VADAGILDDGQQRRR | 0.3 |
| HLA-DRB4*01:01 | 284-298 | 15 | WKRGLAHLDLDLDDP | 0.32 |
| HLA-DRB1*01:01 | 136-150 | 15 | LGRLWLWLSGKLARD | 0.32 |
| HLA-DQA1*01:02/DQB1*06:02 | 146-160 | 15 | KLARDARAAHLAPAL | 0.33 |
| HLA-DRB1*07:01 | 9-23 | 15 | LDALWLTEAVRLREE | 0.41 |
| HLA-DRB1*15:01 | 376-390 | 15 | LEQLTRYPPARLAIA | 0.5 |
| HLA-DPA1*02:01/DPB1*14:01 | 238-252 | 15 | ELIRASGDAALASEA | 0.55 |
