# Supplementary material for: Structural Characterization and Functional Annotation of Hypothetical Proteins in the Multidrug‐Resistant Strains of Pseudomonas aeruginosa
Source: Biomed Res Int. 2026 Feb 2;2026:2974616. doi: 10.1155/bmri/2974616 (PMC12864544; doi:10.1155/bmri/2974616)
Supplement: Supplementary file 5 — Supporting Information 5 Table S3. B cell epitopes identified in hypothetical proteins. [file BMRI-2026-2974616-s005.docx]

| **Score** | **Start position** | **Sequence** | **Rank** | **Name of protein** | |
| --- | --- | --- | --- | --- | --- |
| 0.94 | 63 | VNRVYDGREGFAGLSP | 1 | HP1 |  |
| 0.93 | 39 | DGKRFHTERRRYLASP | 2 |  |  |
| 0.93 | 151 | LRMPTWADEPDRDCEA | 2 |  |  |
| 0.84 | 13 | LILPTTAARNDGLCIP | 3 |  |  |
| 0.83 | 85 | AVSVLSGEVHNGGFDQ | 4 |  |  |
| 0.83 | 104 | NSSGDQYQAARAGLRE | 4 |  |  |
| 0.82 | 31 | GGYQQNIEDGKRFHTE | 5 |  |  |
| 0.76 | 72 | GFAGLSPAERSYYAVS | 6 |  |  |
| 0.76 | 22 | NDGLCIPCKGGYQQNI | 6 |  |  |
| 0.73 | 50 | YLASPQALYWSALVNR | 7 |  |  |
| 0.73 | 110 | YQAARAGLRELEAKDA | 7 |  |  |
| 0.72 | 134 | ALLFGDGPVPVSQAER | 8 |  |  |
| 0.70 | 164 | CEAALDALDTRFYALA | 9 |  |  |
| 0.69 | 94 | HNGGFDQYFGNSSGDQ | 10 |  |  |
| 0.69 | 128 | LLERAAALLFGDGPVP | 10 |  |  |
| 0.67 | 157 | ADEPDRDCEAALDALD | 11 |  |  |
| 0.63 | 122 | AKDALALLERAAALLF | 12 |  |  |
| 0.55 | 179 | ADALGERLLAHARHHA | 13 |  |  |

**Supplementary Table S3.** **B Cell Epitopes Identified in Hypothetical Proteins**

| 0.97 | 95 | AGREDYKPESKAFCYS | 1 | HP2 |
| --- | --- | --- | --- | --- |
| 0.90 | 60 | SFSGPGPAKGNAAVYD | 2 |  |
| 0.90 | 139 | TVEVKPREVKDWAKNP | 2 |  |
| 0.87 | 87 | GDSYDLTPAGREDYKP | 3 |  |
| 0.86 | 7 | RGLVAALPLFLAACSD | 4 |  |
| 0.82 | 119 | SVDPAKPDDYGPAVEK | 5 |  |
| 0.81 | 129 | GPAVEKGWLVTVEVKP | 6 |  |
| 0.79 | 176 | VSLVKPRGEEGYKLVN | 7 |  |
| 0.79 | 165 | LQQITQPQVGQVSLVK | 7 |  |
| 0.77 | 78 | VGVGLLRRDGDSYDLT | 8 |  |
| 0.76 | 27 | SEEIARLLAERGFDKP | 9 |  |
| 0.75 | 42 | PACASSTLFKTFPVTL | 10 |  |
| 0.73 | 182 | RGEEGYKLVNTRFSPR | 11 |  |
| 0.72 | 35 | AERGFDKPACASSTLF | 12 |  |
| 0.71 | 18 | AACSDSAPSSEEIARL | 13 |  |
| 0.70 | 155 | EVLKQASLTTLQQITQ | 14 |  |
| 0.70 | 104 | SKAFCYSSGFDVSVRS | 14 |  |
| 0.68 | 149 | DWAKNPEVLKQASLTT | 15 |  |
| 0.64 | 190 | VNTRFSPRQGFHFNQA | 16 |  |

| 0.87 | 171 | LEAWLADSAGDAVVLR | 1 | HP3 |
| --- | --- | --- | --- | --- |
| 0.86 | 5 | LPLRDAWFFFTRHLTT | 2 |  |
| 0.85 | 71 | MDARGRDERPRVGQLW | 3 |  |
| 0.82 | 177 | DSAGDAVVLRVALHTL | 4 |  |
| 0.79 | 36 | QQQIDEAVGPQQMGAW | 5 |  |
| 0.79 | 140 | LQALRESFEFTRGRFF | 5 |  |
| 0.75 | 50 | AWSLVAGLLFYPLYTA | 6 |  |
| 0.71 | 65 | AALILFMDARGRDERP | 7 |  |
| 0.69 | 83 | GQLWSAALRLWPAFAL | 8 |  |
| 0.69 | 187 | VALHTLSGFFQLLLTI | 8 |  |
| 0.69 | 111 | LSLLILPGIFVMVKLS | 8 |  |
| 0.68 | 44 | GPQQMGAWSLVAGLLF | 9 |  |
| 0.68 | 199 | LLTIVAFRIYSLGPAP | 9 |  |
| 0.65 | 118 | GIFVMVKLSFAEFCLV | 10 |  |
| 0.63 | 146 | SFEFTRGRFFLILACS | 11 |  |
| 0.58 | 160 | CSLVILLPVWSLEAWL | 12 |  |
| 0.57 | 12 | FFFTRHLTTLVPLCLP | 13 |  |

| 0.89 | 126 | AFEVQGTDDPQRFRYK | 1 | HP4 |
| --- | --- | --- | --- | --- |
| 0.87 | 39 | TGADAARSEADAQRQE | 2 |  |
| 0.85 | 3 | GWELQFRDPRRAWLVR | 3 |  |
| 0.84 | 214 | PKLIDVRAELNGQSKP | 4 |  |
| 0.81 | 194 | FQSIPEGGRFAELELP | 5 |  |
| 0.77 | 67 | LRTEIEVLRSGERLSQ | 6 |  |
| 0.75 | 79 | RLSQQATEQSRQTIKL | 7 |  |
| 0.75 | 147 | LGRDDRKLDGRLKVRI | 7 |  |
| 0.74 | 179 | SPELGKTGLSISLRHF | 8 |  |
| 0.74 | 164 | GKLARKDATYDLEQLS | 8 |  |
| 0.72 | 99 | VFKQQQDIAFYKGVVA | 9 |  |
| 0.72 | 223 | LNGQSKPLQRRFDWPK | 9 |  |
| 0.72 | 14 | AWLVRLGVGALLLAVP | 9 |  |
| 0.71 | 28 | VPLAFLGGRWSTGADA | 10 |  |
| 0.70 | 45 | RSEADAQRQETLIGEQ | 11 |  |
| 0.68 | 89 | RQTIKLLEDQVFKQQQ | 12 |  |
| 0.68 | 54 | ETLIGEQKAELERLRT | 12 |  |
| 0.65 | 61 | KAELERLRTEIEVLRS | 13 |  |
| 0.65 | 106 | IAFYKGVVAPASKADA | 13 |  |
| 0.63 | 115 | PASKADALEIRAFEVQ | 14 |  |
| 0.62 | 185 | TGLSISLRHFQSIPEG | 15 |  |
| 0.58 | 200 | GGRFAELELPKNFIPK | 16 |  |
| 0.53 | 134 | DPQRFRYKVMLSRLGR | 17 |  |

| 0.94 | 22 | QRLYQAAGWPDQRAHF | 1 | HP5 |
| --- | --- | --- | --- | --- |
| 0.93 | 202 | TLLYVYRDLSDAELTQ | 2 |  |
| 0.86 | 129 | SEPRRALVKRLAQVLP | 3 |  |
| 0.85 | 79 | NGLRENLPDSASPLNF | 4 |  |
| 0.82 | 54 | PALYQTLVDNSNRRFA | 5 |  |
| 0.79 | 122 | GLPRQTASEPRRALVK | 6 |  |
| 0.78 | 64 | SNRRFAPAEVDQRALN | 7 |  |
| 0.77 | 38 | SDALDAAQKRYQDKLP | 8 |  |
| 0.76 | 45 | QKRYQDKLPPALYQTL | 9 |  |
| 0.76 | 235 | LAALKAGLAVDQAVAP | 9 |  |
| 0.76 | 152 | VSLALAGVAADSLSQM | 9 |  |
| 0.76 | 100 | GRKVVAAEVLATRRDQ | 9 |  |
| 0.75 | 222 | AESPAGRSYYQAALAA | 10 |  |
| 0.75 | 158 | GVAADSLSQMLPGLPG | 10 |  |
| 0.74 | 166 | QMLPGLPGLLGGGQTG | 11 |  |
| 0.73 | 178 | GQTGQLLEGQRQRLVQ | 12 |  |
| 0.71 | 29 | GWPDQRAHFSDALDAA | 13 |  |
| 0.70 | 72 | EVDQRALNGLRENLPD | 14 |  |
| 0.68 | 192 | VQQIGADLENTLLYVY | 15 |  |
| 0.68 | 137 | KRLAQVLPAREAGAEV | 15 |  |
| 0.64 | 210 | LSDAELTQYVNFAESP | 16 |  |
| 0.63 | 186 | GQRQRLVQQIGADLEN | 17 |  |
| 0.63 | 115 | QLLQHSNGLPRQTASE | 17 |  |
| 0.62 | 109 | LATRRDQLLQHSNGLP | 18 |  |
|  |  |  |  |  |

| 0.91 | 262 | TGSRTSTPTLNMEFTF | 1 | Hp6 |
| --- | --- | --- | --- | --- |
| 0.91 | 227 | TALMVAALPGDKRQPN | 1 |  |
| 0.90 | 184 | NLTWMRPFKVGNHAAK | 2 |  |
| 0.90 | 164 | HFGVPGARHPDHTFDT | 2 |  |
| 0.89 | 29 | SVGFRYGQQFTNPNNP | 3 |  |
| 0.89 | 247 | VGYEYWHNKFGVDGGT | 3 |  |
| 0.89 | 172 | HPDHTFDTTYMLNLTW | 3 |  |
| 0.89 | 131 | ASAKKRALVFGPTLKF | 3 |  |
| 0.86 | 17 | AGESMALEWMSNSVGF | 4 |  |
| 0.86 | 118 | FTLGFDASRNNNLASA | 4 |  |
| 0.83 | 85 | HGGSEVYAVYRHQLYA | 5 |  |
| 0.83 | 48 | AKRIYSFTHADGYRYG | 5 |  |
| 0.82 | 100 | ASRVFDRPLGTGLVKD | 6 |  |
| 0.81 | 58 | DGYRYGSNFFNLDVFL | 7 |  |
| 0.81 | 23 | LEWMSNSVGFRYGQQF | 7 |  |
| 0.81 | 208 | GEKGEDYNDKDTAAET | 7 |  |
| 0.76 | 35 | GQQFTNPNNPDKFAKR | 8 |  |
| 0.75 | 217 | KDTAAETLVRTALMVA | 9 |  |
| 0.74 | 70 | DVFLSDNRDPRKGTDH | 10 |  |
| 0.74 | 191 | FKVGNHAAKFQGFVNY | 10 |  |
| 0.73 | 149 | PGVLDLSLLYYREKNH | 11 |  |
| 0.72 | 77 | RDPRKGTDHGGSEVYA | 12 |  |
| 0.71 | 9 | AALIALVGAGESMALE | 13 |  |
| 0.67 | 178 | DTTYMLNLTWMRPFKV | 14 |  |
| 0.66 | 235 | PGDKRQPNLWLGVGYE | 15 |  |
| 0.59 | 138 | LVFGPTLKFNGPGVLD | 16 |  |

| 0.94 | 195 | GHQQRYPLRWTARAQD | 1 | HP7 |
| --- | --- | --- | --- | --- |
| 0.93 | 131 | LILLDAAEFSGAPCQP | 2 |  |
| 0.92 | 30 | IRPEQWPLDPDTGYPL | 3 |  |
| 0.90 | 252 | PNHIGGTMRPVQGVPE | 4 |  |
| 0.90 | 243 | EHPWVEGHLPNHIGGT | 4 |  |
| 0.90 | 202 | LRWTARAQDPNAGVVP | 4 |  |
| 0.89 | 89 | EMLIDPPAQPPAEAEL | 5 |  |
| 0.87 | 266 | PEMSPFYVEFEEYLGG | 6 |  |
| 0.87 | 21 | GWCFGLPPGIRPEQWP | 6 |  |
| 0.87 | 13 | AHAGEANHGWCFGLPP | 6 |  |
| 0.85 | 80 | GPTSVDGIREMLIDPP | 7 |  |
| 0.85 | 176 | DWAEDDYYLLRALGGR | 7 |  |
| 0.84 | 275 | FEEYLGGYNFGGGNAQ | 8 |  |
| 0.84 | 233 | DGKIERDNYREHPWVE | 8 |  |
| 0.82 | 51 | LLLPEDYRVHGPEIVA | 9 |  |
| 0.82 | 213 | AGVVPSEYGDGGYQLP | 9 |  |
| 0.82 | 105 | RPFWEAGRNSHPRLHR | 9 |  |
| 0.81 | 284 | FGGGNAQLDFRDMRFD | 10 |  |
| 0.81 | 225 | YQLPHYWLDGKIERDN | 10 |  |
| 0.79 | 117 | RLHRMEDILGGAYALI | 11 |  |
| 0.78 | 186 | RALGGRPAAGHQQRYP | 12 |  |
| 0.77 | 99 | PAEAELRPFWEAGRNS | 13 |  |
| 0.77 | 36 | PLDPDTGYPLMHGFTL | 13 |  |
| 0.76 | 68 | SFFSLAPDQNDGGPTS | 14 |  |
| 0.76 | 60 | HGPEIVALSFFSLAPD | 14 |  |
| 0.75 | 167 | AGSAASFVPDWAEDDY | 15 |  |
| 0.71 | 140 | SGAPCQPPALPAGNPL | 16 |  |
| 0.67 | 259 | MRPVQGVPEMSPFYVE | 17 |  |
| 0.64 | 146 | PPALPAGNPLLAQTEP | 18 |  |
| 0.62 | 154 | PLLAQTEPPQWLEAGS | 19 |  |
| 0.55 | 125 | LGGAYALILLDAAEFS | 20 |  |

| 0.93 | 67 | AFEIFQGDTRDGYANT | 1 | HP8 |
| --- | --- | --- | --- | --- |
| 0.92 | 97 | DMVLFYGEEFHAAWTP | 2 |  |
| 0.92 | 34 | ADLIESGIDSDETLAA | 2 |  |
| 0.89 | 17 | TGEWKSRCVYVATRLG | 3 |  |
| 0.89 | 113 | ACEALLSGTPGFELAF | 3 |  |
| 0.88 | 184 | AILQAEPSARGVMLDR | 4 |  |
| 0.87 | 288 | DVHLFMACAGRHRTTE | 5 |  |
| 0.87 | 260 | EAMAGDGRVVVIERTI | 5 |  |
| 0.85 | 278 | SEPSPMSVLWDVHLFM | 6 |  |
| 0.85 | 267 | RVVVIERTISASEPSP | 6 |  |
| 0.83 | 77 | DGYANTPTSHLLRDVE | 7 |  |
| 0.83 | 126 | LAFGEDFYSYLKRCPD | 7 |  |
| 0.81 | 195 | VMLDREGSLGVARDNL | 8 |  |
| 0.81 | 107 | HAAWTPACEALLSGTP | 8 |  |
| 0.79 | 309 | LGRGGFAVERIVDLPM | 9 |  |
| 0.77 | 23 | RCVYVATRLGLADLIE | 10 |  |
| 0.76 | 157 | FHEIPRLLDFRGRSFV | 11 |  |
| 0.76 | 137 | KRCPDAGRRFLLAMKA | 11 |  |
| 0.75 | 315 | AVERIVDLPMETRMIV | 12 |  |
| 0.75 | 240 | SRIIGDLDEAASLRLL | 12 |  |
| 0.74 | 225 | DMLQEVPSNGDIYLLS | 13 |  |
| 0.74 | 173 | DVGGGSGELTKAILQA | 13 |  |
| 0.73 | 44 | DETLAAAVGSDAERIH | 14 |  |
| 0.73 | 231 | PSNGDIYLLSRIIGDL | 14 |  |
| 0.72 | 295 | CAGRHRTTEEVVDLLG | 15 |  |
| 0.71 | 86 | HLLRDVEGSFRDMVLF | 16 |  |
| 0.66 | 302 | TEEVVDLLGRGGFAVE | 17 |  |
| 0.64 | 206 | ARDNLSSLLAGERVSL | 18 |  |
| 0.61 | 53 | SDAERIHRLMRLLVAF | 19 |  |
| 0.51 | 59 | HRLMRLLVAFEIFQGD | 20 |  |

| 0.88 | 94 | PNRAGQPVDFDRPQDM | 1 | HP9 |
| --- | --- | --- | --- | --- |
| 0.88 | 85 | VPEVKGWLVPNRAGQP | 1 |  |
| 0.88 | 257 | LRFSQAPGQPSPVFSP | 1 |  |
| 0.86 | 151 | GRLEVEGRVRPLGVGP | 2 |  |
| 0.85 | 298 | AQVLLKTETPDQVLTF | 3 |  |
| 0.84 | 56 | AGPADSGQDGVGILLR | 4 |  |
| 0.84 | 237 | PLLGIDGHVQEAWLDD | 4 |  |
| 0.84 | 216 | LDRPGASLVGSAMRLD | 4 |  |
| 0.83 | 41 | YRQVPPAPVPLPMRTA | 5 |  |
| 0.81 | 304 | TETPDQVLTFNLQDYR | 6 |  |
| 0.8 | 183 | GALVYRPDQLRVIGLP | 7 |  |
| 0.79 | 248 | AWLDDQGLHLRFSQAP | 8 |  |
| 0.79 | 100 | PVDFDRPQDMHFQVME | 8 |  |
| 0.78 | 78 | RGNIGFHVPEVKGWLV | 9 |  |
| 0.77 | 50 | PLPMRTAGPADSGQDG | 10 |  |
| 0.77 | 330 | NEDGTFFMQVPPYANP | 10 |  |
| 0.77 | 324 | TGTTQVNEDGTFFMQV | 10 |  |
| 0.76 | 292 | EILITYAQVLLKTETP | 11 |  |
| 0.76 | 175 | GVEVDAASGALVYRPD | 11 |  |
| 0.75 | 139 | TMLKDFRMSSAAGRLE | 12 |  |
| 0.75 | 115 | EGEVLLSPRQLANLFN | 12 |  |
| 0.74 | 313 | FNLQDYRKVLATGTTQ | 13 |  |
| 0.74 | 222 | SLVGSAMRLDYRKVFP | 13 |  |
| 0.73 | 66 | VGILLRNVDMYLRGNI | 14 |  |
| 0.73 | 191 | QLRVIGLPLYGAMSLV | 14 |  |
| 0.71 | 163 | GVGPWLPLRLGGGVEV | 15 |  |
| 0.7 | 23 | ESAIDRQQRELALLRE | 16 |  |
| 0.69 | 271 | SPPAAAGPSFLWLQSG | 17 |  |
| 0.67 | 133 | ILAYDGTMLKDFRMSS | 18 |  |
| 0.62 | 263 | PGQPSPVFSPPAAAGP | 19 |  |
| 0.62 | 198 | PLYGAMSLVGLPMSAL | 19 |  |
| 0.61 | 11 | GCCLLAASAAQAESAI | 20 |  |
| 0.55 | 277 | GPSFLWLQSGDLKIFE | 21 |  |
| 0.51 | 2 | SRRPWGVLLGCCLLAA | 22 |  |

| 0.91 | 339 | ARERLDALEPIIRRVE | 1 | HP10 |
| --- | --- | --- | --- | --- |
| 0.91 | 215 | AREGELPPVLALRLNC | 1 |  |
| 0.9 | 157 | RSVGRDALLPDWRFYS | 2 |  |
| 0.89 | 80 | SVPPIDSPTLEALRGL | 3 |  |
| 0.88 | 37 | QSGGQARIRLMRGDQP | 4 |  |
| 0.88 | 234 | CVILDGHCRLRAGLLE | 4 |  |
| 0.86 | 281 | AQRVDAPVRRGRRPLD | 5 |  |
| 0.86 | 181 | WVQWLDWWGRDNDALI | 5 |  |
| 0.86 | 16 | EWLGLAVQVEGAHRAP | 5 |  |
| 0.85 | 101 | YKAWSRYFVETLRTSP | 6 |  |
| 0.84 | 109 | VETLRTSPGSCLEAGR | 7 |  |
| 0.83 | 151 | LERWRYRSVGRDALLP | 8 |  |
| 0.82 | 265 | PMPVDTAQRERVLQSL | 9 |  |
| 0.82 | 140 | PPQSHDPRERVLERWR | 9 |  |
| 0.82 | 116 | PGSCLEAGRWLLRLSL | 9 |  |
| 0.81 | 90 | EALRGLRGEARYKAWS | 10 |  |
| 0.81 | 330 | CAEVRDWLAARERLDA | 10 |  |
| 0.8 | 206 | GRVKWWRKKAREGELP | 11 |  |
| 0.79 | 309 | DDRPWPRVLTRARAVL | 12 |  |
| 0.78 | 287 | PVRRGRRPLDSEQLNQ | 13 |  |
| 0.78 | 199 | RRVEDDEGRVKWWRKK | 13 |  |
| 0.77 | 68 | VKSLVAASPAQASVPP | 14 |  |
| 0.77 | 52 | PLLWATQLPYHDGIWL | 14 |  |
| 0.77 | 134 | QVPAWQPPQSHDPRER | 14 |  |
| 0.76 | 2 | HTLYWRDLESPANGEW | 15 |  |
| 0.72 | 303 | VLLRLFDDRPWPRVLT | 16 |  |
| 0.72 | 256 | LVLCAYDEQPMPVDTA | 16 |  |
| 0.72 | 166 | PDWRFYSLEKVLDDDW | 16 |  |
| 0.65 | 61 | YHDGIWLVKSLVAASP | 17 |  |
| 0.65 | 45 | RLMRGDQPLLWATQLP | 17 |  |
| 0.64 | 22 | VQVEGAHRAPSFLHLQ | 18 |  |
| 0.63 | 122 | AGRWLLRLSLAEQVPA | 19 |  |
| 0.61 | 172 | SLEKVLDDDWVQWLDW | 20 |  |
| 0.6 | 8 | DLESPANGEWLGLAVQ | 21 |  |
| 0.58 | 74 | ASPAQASVPPIDSPTL | 22 |  |
| 0.58 | 241 | CRLRAGLLENVAPEIL | 22 |  |

| 0.97 | 288 | YGCHEYSNEFDRACGP | 1 | HP11 |
| --- | --- | --- | --- | --- |
| 0.93 | 161 | SGVIGLPREEAKILDR | 2 |  |
| 0.89 | 312 | NAIERYPQEPIAEQIE | 3 |  |
| 0.89 | 217 | HHYWSRKQLFRAKIQA | 3 |  |
| 0.89 | 146 | LFERGWADDEMHQLNS | 3 |  |
| 0.87 | 59 | QVREVTAEELVAWCQP | 4 |  |
| 0.86 | 70 | AWCQPHGYHFRAKHVA | 5 |  |
| 0.86 | 252 | KVTDRLPRPPRAKRLL | 5 |  |
| 0.85 | 43 | VRVYTDDPSPYAGLPV | 6 |  |
| 0.85 | 303 | PVWWEKAATNAIERYP | 6 |  |
| 0.84 | 78 | HFRAKHVALRDALEDA | 7 |  |
| 0.84 | 35 | DGAAHGLTVRVYTDDP | 7 |  |
| 0.83 | 297 | FDRACGPVWWEKAATN | 8 |  |
| 0.82 | 268 | YKLGTLPLPAPMRQFS | 9 |  |
| 0.8 | 277 | APMRQFSIELLYGCHE | 10 |  |
| 0.8 | 139 | YRDLSPYLFERGWADD | 10 |  |
| 0.8 | 109 | PLRLFDRIQPGSLLCN | 10 |  |
| 0.78 | 128 | ARYGEHRNFPLYRDLS | 11 |  |
| 0.77 | 318 | PQEPIAEQIEDWLNHS | 12 |  |
| 0.77 | 171 | AKILDRSLELMDELYP | 12 |  |
| 0.77 | 122 | LCNAIGARYGEHRNFP | 12 |  |
| 0.76 | 235 | CKHGSSLLSAAALDDT | 13 |  |
| 0.75 | 13 | YGRQTYHQEALFSIAS | 14 |  |
| 0.74 | 228 | AKIQAWLCKHGSSLLS | 15 |  |
| 0.72 | 209 | LVGCTDLIHHYWSRKQ | 16 |  |
| 0.7 | 186 | PIANGAYTLEEFVLAV | 17 |  |
| 0.7 | 180 | LMDELYPIANGAYTLE | 17 |  |
| 0.69 | 6 | KHLIYLVYGRQTYHQE | 18 |  |
| 0.68 | 96 | ALLIDTDTFFHSSPLR | 19 |  |
| 0.67 | 49 | DPSPYAGLPVQVREVT | 20 |  |
| 0.67 | 330 | LNHSLLRNLLGSRANE | 20 |  |
| 0.67 | 152 | ADDEMHQLNSGVIGLP | 20 |  |
| 0.66 | 324 | EQIEDWLNHSLLRNLL | 21 |  |
| 0.65 | 103 | TFFHSSPLRLFDRIQP | 22 |  |
| 0.63 | 84 | VALRDALEDADQALLI | 23 |  |
| 0.63 | 258 | PRPPRAKRLLYKLGTL | 23 |  |
| 0.62 | 340 | GSRANEIRQHLRQRDL | 24 |  |
| 0.61 | 26 | IASAFAHLRDGAAHGL | 25 |  |
| 0.56 | 200 | AVAAHKRLELVGCTDL | 26 |  |

| 0.96 | 114 | YESGKAPDEFSSGGAA | 1 | HP12 |
| --- | --- | --- | --- | --- |
| 0.94 | 245 | DVDYTYEIDDNWSLNP | 2 |  |
| 0.93 | 368 | RVDPDSPGYGGWYSAD | 3 |  |
| 0.92 | 217 | GGTWGGIEGFTSSLYA | 4 |  |
| 0.91 | 267 | TVDSGDSLLGRIDNNT | 5 |  |
| 0.91 | 154 | GESRMLPQTFRGVSLT | 5 |  |
| 0.91 | 123 | FSSGGAALKIRAFDTE | 5 |  |
| 0.9 | 293 | QHTVTAVLQKVNGNTP | 6 |  |
| 0.89 | 376 | YGGWYSADGKNAKHWE | 7 |  |
| 0.86 | 69 | FQSGYTPGVVGFGVDA | 8 |  |
| 0.86 | 336 | SWKLQYDYDFVALGLP | 8 |  |
| 0.86 | 323 | SQQYSDFNGPNEKSWK | 8 |  |
| 0.86 | 107 | TAPSKEGYESGKAPDE | 8 |  |
| 0.85 | 199 | SYYGELPGDRDSHHLS | 9 |  |
| 0.83 | 93 | DGGGGTGGTSILPITA | 10 |  |
| 0.83 | 56 | HDSKEWAQGFIATFQS | 10 |  |
| 0.82 | 99 | GGTSILPITAPSKEGY | 11 |  |
| 0.82 | 424 | SAVDNDIDEYRVIVDY | 11 |  |
| 0.82 | 417 | HRGTGGYSAVDNDIDE | 11 |  |
| 0.82 | 308 | PFDYINQGDSIFLDNS | 11 |  |
| 0.82 | 22 | ADEGEAKEGFIEGSSL | 11 |  |
| 0.81 | 79 | GFGVDAYGMLGLKLDG | 12 |  |
| 0.81 | 351 | PGLSASASYSRGKLDL | 12 |  |
| 0.8 | 359 | YSRGKLDLTRVDPDSP | 13 |  |
| 0.8 | 211 | HHLSWLGGTWGGIEGF | 13 |  |
| 0.8 | 184 | SFTKYYNQSGHRRLGS | 13 |  |
| 0.79 | 389 | HWERDLDLQYVVQGGP | 14 |  |
| 0.78 | 255 | NWSLNPGAHYYKTVDS | 15 |  |
| 0.77 | 47 | NHDRRHASGHDSKEWA | 16 |  |
| 0.76 | 16 | TASAAWADEGEAKEGF | 17 |  |
| 0.75 | 33 | EGSSLQLLTRNYYFNH | 18 |  |
| 0.74 | 205 | PGDRDSHHLSWLGGTW | 19 |  |
| 0.72 | 85 | YGMLGLKLDGGGGTGG | 20 |  |
| 0.72 | 8 | TPMALALCTASAAWAD | 20 |  |
| 0.7 | 147 | SNPVVAGGESRMLPQT | 21 |  |
| 0.69 | 39 | LLTRNYYFNHDRRHAS | 22 |  |
| 0.67 | 302 | KVNGNTPFDYINQGDS | 23 |  |
| 0.67 | 275 | LGRIDNNTYSLHFAVG | 23 |  |
| 0.67 | 233 | AELQNVWKQYYADVDY | 23 |  |
| 0.66 | 283 | YSLHFAVGYRQHTVTA | 24 |  |
| 0.66 | 227 | TSSLYAAELQNVWKQY | 24 |  |
| 0.65 | 129 | ALKIRAFDTELKLGDQ | 25 |  |
| 0.63 | 141 | LGDQFLSNPVVAGGES | 26 |  |
| 0.62 | 399 | VVQGGPAKDLSLRLRW | 27 |  |
| 0.62 | 315 | GDSIFLDNSQQYSDFN | 27 |  |
| 0.61 | 169 | TNNSFEDLTLTAGQVS | 28 |  |
| 0.58 | 1 | MRNLFALTPMALALCT | 29 |  |

| 0.95 | 291 | DMYNGAPDRYDWKLEG | 1 | HP13 |
| --- | --- | --- | --- | --- |
| 0.92 | 358 | ERHIYAKRDFYIDEDT | 2 |  |
| 0.91 | 307 | KKEIYIPYNSYKLDDP | 3 |  |
| 0.9 | 275 | DGPGTAADGLRTSDNF | 4 |  |
| 0.9 | 143 | LVEGGNGLENFDTANP | 4 |  |
| 0.89 | 83 | LFTITAQNVDQYKDKL | 5 |  |
| 0.89 | 297 | PDRYDWKLEGKKEIYI | 5 |  |
| 0.88 | 437 | YSASESDYTPAALRQE | 6 |  |
| 0.88 | 256 | AWLYNAGQRRVRRAPQ | 6 |  |
| 0.88 | 25 | AVSADEAAKLGTSLTP | 6 |  |
| 0.88 | 101 | GQLAMFKRYPDTYKIP | 6 |  |
| 0.87 | 375 | QAAEIDHYDGRGTLWR | 7 |  |
| 0.87 | 196 | YQLVYFQDAFTFRTNL | 7 |  |
| 0.87 | 184 | LVTQATPQVNGSYQLV | 7 |  |
| 0.87 | 112 | TYKIPVYKTHRSATVP | 7 |  |
| 0.85 | 349 | HVVATLKPGERHIYAK | 8 |  |
| 0.85 | 123 | SATVPAAVQEAAKRNA | 8 |  |
| 0.84 | 326 | YSEIVKAGHINQDLTR | 9 |  |
| 0.83 | 50 | DGSIPAWDGGLATNAG | 10 |  |
| 0.83 | 424 | GMKNEEKQAYDFNYSA | 10 |  |
| 0.83 | 391 | VAEAHAEQYYDKQVPW | 10 |  |
| 0.83 | 381 | HYDGRGTLWRVAEAHA | 10 |  |
| 0.82 | 366 | DFYIDEDTWQAAEIDH | 11 |  |
| 0.81 | 172 | HITRYRGGSVRRLVTQ | 12 |  |
| 0.81 | 166 | LEVIWNHITRYRGGSV | 12 |  |
| 0.8 | 204 | AFTFRTNLKDYNPNKP | 13 |  |
| 0.78 | 282 | DGLRTSDNFDMYNGAP | 14 |  |
| 0.76 | 137 | NATTTKLVEGGNGLEN | 15 |  |
| 0.75 | 91 | VDQYKDKLTPGQLAMF | 16 |  |
| 0.75 | 332 | AGHINQDLTRYELHRV | 16 |  |
| 0.75 | 229 | RVTAPSRLAGNVLLVH | 16 |  |
| 0.74 | 39 | TPLGAEKAGNADGSIP | 17 |  |
| 0.73 | 264 | RRVRRAPQVSYDGPGT | 18 |  |
| 0.7 | 68 | DSRGFLANPYASEQPL | 19 |  |
| 0.7 | 58 | GGLATNAGSVDSRGFL | 19 |  |
| 0.68 | 75 | NPYASEQPLFTITAQN | 20 |  |
| 0.68 | 408 | AVETLYDLLSGRYLAL | 20 |  |
| 0.68 | 397 | EQYYDKQVPWYAVETL | 20 |  |
| 0.68 | 14 | ALSLLATQVMAAVSAD | 20 |  |
| 0.65 | 416 | LSGRYLALGMKNEEKQ | 21 |  |
| 0.65 | 340 | TRYELHRVWHVVATLK | 21 |  |
| 0.65 | 242 | LVHETLNQVKEPRLAW | 21 |  |
| 0.64 | 130 | VQEAAKRNATTTKLVE | 22 |  |
| 0.62 | 150 | LENFDTANPFPIPQNG | 23 |  |
| 0.6 | 431 | QAYDFNYSASESDYTP | 24 |  |
| 0.58 | 159 | FPIPQNGLEVIWNHIT | 25 |  |
| 0.52 | 219 | PSNVLFYFKQRVTAPS | 26 |  |
| 0.52 | 210 | NLKDYNPNKPSNVLFY | 26 |  |

| 0.96 | 56 | LELYDTPGLEDAIALL | 1 | HP14 |
| --- | --- | --- | --- | --- |
| 0.95 | 80 | PGERLDGPERMARLLD | 2 |  |
| 0.88 | 426 | HPEWSSLNGRRVGGSD | 3 |  |
| 0.88 | 403 | LETPEDKQWREGKLPE | 3 |  |
| 0.86 | 346 | GGAWQTVGHYGSRLLG | 4 |  |
| 0.85 | 35 | HRPSTTRHVEGARLSV | 5 |  |
| 0.83 | 287 | DLPLLDGRWGDDLFNP | 6 |  |
| 0.82 | 102 | RFEQEAKVVRQLLASD | 7 |  |
| 0.81 | 94 | LDSNEARGRFEQEAKV | 8 |  |
| 0.8 | 170 | RLGLHALVRFDSVAPP | 9 |  |
| 0.79 | 420 | LRKARAHPEWSSLNGR | 10 |  |
| 0.79 | 242 | LVPGGESAVQEATRDL | 10 |  |
| 0.79 | 13 | TNTGKTSLLRTLTRDR | 10 |  |
| 0.78 | 439 | GSDRQEQIDALAELLE | 11 |  |
| 0.78 | 397 | AVEAIRLETPEDKQWR | 11 |  |
| 0.78 | 277 | AFARDDARAADLPLLD | 11 |  |
| 0.78 | 262 | RKREQACVEALLRLYA | 11 |  |
| 0.78 | 135 | DELAVLAGCGRPLLPV | 11 |  |
| 0.77 | 313 | GGGVAAGAAAGAGIDL | 12 |  |
| 0.77 | 26 | RDRGFGEVSHRPSTTR | 12 |  |
| 0.75 | 19 | SLLRTLTRDRGFGEVS | 13 |  |
| 0.75 | 125 | AREPVLAKYRDELAVL | 13 |  |
| 0.74 | 391 | QTRGHAAVEAIRLETP | 14 |  |
| 0.74 | 153 | FVASPQHREEEWRAAL | 14 |  |
| 0.73 | 414 | GKLPEALRKARAHPEW | 15 |  |
| 0.73 | 337 | AAAALGALAGGAWQTV | 15 |  |
| 0.73 | 208 | DRLIADHETQAAARLR | 15 |  |
| 0.71 | 62 | PGLEDAIALLDYLDAL | 16 |  |
| 0.71 | 358 | RLLGKLKGARELTVND | 16 |  |
| 0.71 | 3 | EPLILAVVGHTNTGKT | 16 |  |
| 0.71 | 252 | EATRDLHQQVRKREQA | 16 |  |
| 0.71 | 219 | AARLRAGQRLIAELLV | 16 |  |
| 0.71 | 179 | FDSVAPPLDGERRLYE | 16 |  |
| 0.7 | 42 | HVEGARLSVDGEALLE | 17 |  |
| 0.69 | 70 | LLDYLDALERPGERLD | 18 |  |
| 0.69 | 225 | GQRLIAELLVDVAACR | 18 |  |
| 0.68 | 119 | GLYVIDAREPVLAKYR | 19 |  |
| 0.65 | 86 | GPERMARLLDSNEARG | 20 |  |
| 0.65 | 161 | EEEWRAALARLGLHAL | 20 |  |
| 0.64 | 319 | GAAAGAGIDLLVGGVT | 21 |  |
| 0.62 | 112 | QLLASDAGLYVIDARE | 22 |  |
| 0.6 | 352 | VGHYGSRLLGKLKGAR | 23 |  |
| 0.57 | 141 | AGCGRPLLPVLNFVAS | 24 |  |
| 0.56 | 374 | AVLRLLALRQRQLLAA | 25 |  |
| 0.54 | 186 | LDGERRLYESLALLLE | 26 |  |
| 0.53 | 293 | GRWGDDLFNPETLRQL | 27 |  |

| 0.9 | 377 | EQLTRYPPARLAIACD | 1 | HP15 |
| --- | --- | --- | --- | --- |
| 0.89 | 263 | GVLLVYGVLPRALLGL | 2 |  |
| 0.88 | 343 | AVELDDRRPWPPKLAE | 3 |  |
| 0.86 | 53 | HWLGRREGLLDAQRTW | 4 |  |
| 0.86 | 425 | GEALDSDRLGDWHAAL | 4 |  |
| 0.85 | 318 | DAAPDWLPEPQGGQSS | 5 |  |
| 0.84 | 207 | ETTILGSDTFIALTQA | 6 |  |
| 0.83 | 386 | RLAIACDPRRSPDRGT | 7 |  |
| 0.82 | 20 | LREEQAGPLEDSEAVR | 8 |  |
| 0.82 | 143 | LSGKLARDARAAHLAP | 8 |  |
| 0.81 | 59 | EGLLDAQRTWRQGSRL | 9 |  |
| 0.81 | 36 | QALAQGGSLPRRILTR | 9 |  |
| 0.8 | 195 | GLLATRRYGFVWETTI | 10 |  |
| 0.79 | 362 | DAGILDDGQQRRRLLE | 11 |  |
| 0.78 | 253 | ARHAWAGWLVGVLLVY | 12 |  |
| 0.77 | 88 | AGLAFAALGDGQRPVN | 13 |  |
| 0.77 | 308 | MPASERLGVSDAAPDW | 13 |  |
| 0.74 | 247 | ALASEAARHAWAGWLV | 14 |  |
| 0.73 | 286 | RGLAHLDLDLDDPGYS | 15 |  |
| 0.72 | 328 | QGGQSSQEAAGAVLVA | 16 |  |
| 0.71 | 231 | GFPLPDAELIRASGDA | 17 |  |
| 0.69 | 414 | STRIWLLQAPPGEALD | 18 |  |
| 0.69 | 241 | RASGDAALASEAARHA | 18 |  |
| 0.68 | 96 | GDGQRPVNVFWALASL | 19 |  |
| 0.68 | 77 | ALLLVLALASGAGLAF | 19 |  |
| 0.68 | 12 | LWLTEAVRLREEQAGP | 19 |  |
| 0.67 | 441 | ERLQLPHGETSPLAWL | 20 |  |
| 0.67 | 278 | LLCLWRWKRGLAHLDL | 20 |  |
| 0.67 | 26 | GPLEDSEAVRQALAQG | 20 |  |
| 0.66 | 407 | ELARCAASTRIWLLQA | 21 |  |
| 0.66 | 161 | LVLLGRRRLARWGLGA | 21 |  |
| 0.66 | 127 | LAGGEAAGALGRLWLW | 21 |  |
| 0.66 | 104 | VFWALASLLGLHLLTL | 21 |  |
| 0.64 | 431 | DRLGDWHAALERLQLP | 22 |  |
| 0.64 | 170 | ARWGLGALVHGLWLLG | 22 |  |
| 0.63 | 42 | GSLPRRILTRAHWLGR | 23 |  |
| 0.63 | 292 | DLDLDDPGYSLLRERL | 23 |  |
| 0.63 | 1 | LSHTTHPGLDALWLTE | 23 |  |
| 0.62 | 215 | TFIALTQALGALPALL | 24 |  |
| 0.6 | 67 | TWRQGSRLALALLLVL | 25 |  |
| 0.6 | 151 | ARAAHLAPALLVLLGR | 25 |  |
| 0.58 | 368 | DGQQRRRLLEQLTRYP | 26 |  |
| 0.57 | 397 | PDRGTLALLGELARCA | 27 |  |

Ellipro

| 3D structure | score | Number of residues | residues | rank | Name of protein |
| --- | --- | --- | --- | --- | --- |
| 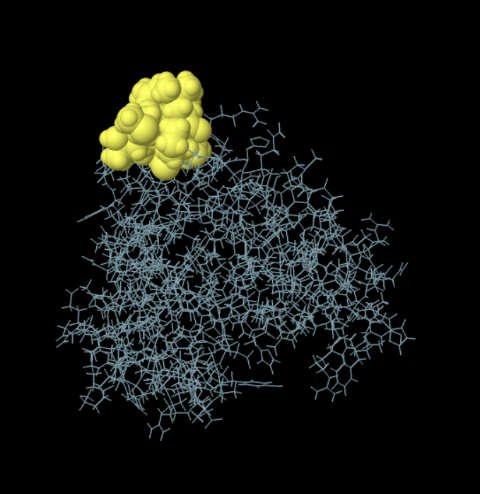 | 0.741 | 7 | A:M1, A:T2, A:D3, A:R4, A:L5, A:P6, A:C7 | 1 | HP1  HP1 |
| 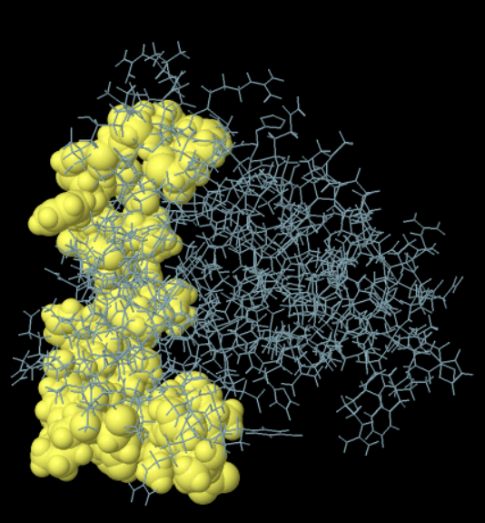 | 0.705 | 40 | A:R65, A:D68, A:G69, A:R70, A:G72, A:F73, A:A74, A:G75, A:L76, A:S77, A:L120, A:E121, A:A122, A:K123, A:D124, A:A177, A:L178, A:A179, A:D180, A:A181, A:E184, A:R185, A:L187, A:A188, A:H189, A:A190, A:R191, A:H192, A:H193, A:A194, A:L195, A:F196, A:A197, A:L198, A:D199, A:E200, A:P201, A:D202, A:E203, A:A204 | 2 |  |
| 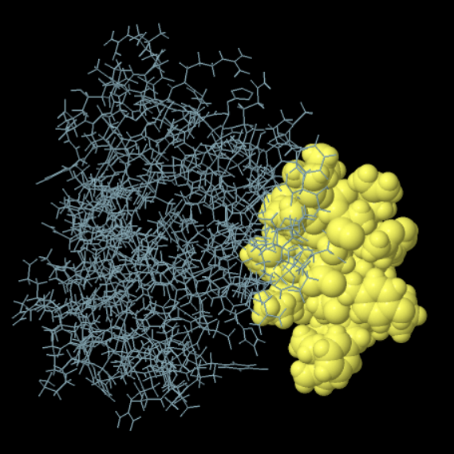 | 0.655 | 32 | A:T17, A:A19, A:A20, A:R21, A:D23, A:G24, A:L25, A:C26, A:P28, A:C29, A:K30, A:G31, A:G32, A:Y33, A:Q34, A:Q35, A:N36, A:I37, A:E38, A:D39, A:G40, A:K41, A:R42, A:F43, A:H44, A:T45, A:E46, A:R47, A:R48, A:R49, A:L51, A:A52 | 3 |  |
| 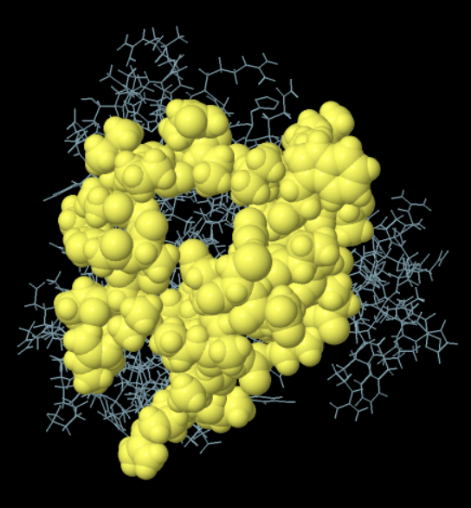 | 0.652 | 33 | A:R131, A:A134, A:L135, A:F137, A:G138, A:D139, A:G140, A:P141, A:V142, A:P143, A:Q146, A:A147, A:L151, A:M153, A:P154, A:T155, A:W156, A:A157, A:D158, A:E159, A:P160, A:D161, A:R162, A:D163, A:C164, A:E165, A:A166, A:A167, A:L168, A:D169, A:A170, A:T173, A:R174 | 4 |  |
| 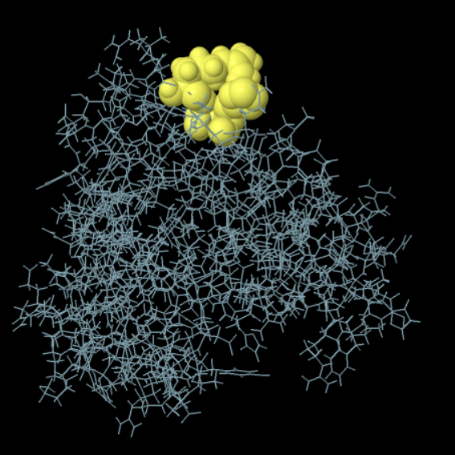 | 0.533 | 3 | A:R8, A:G11, A:H12 | 5 |  |
| 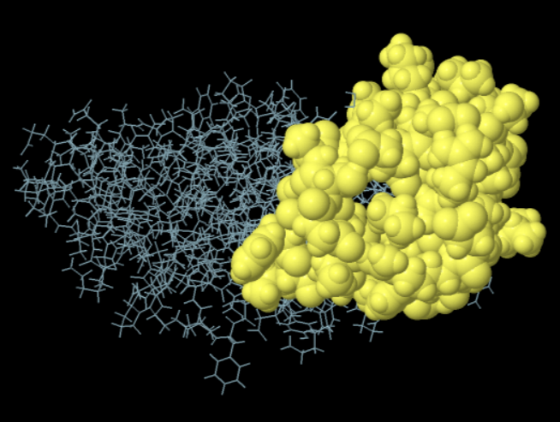 | 0.654 | 52 | A:F16, A:L17, A:A18, A:A19, A:C20, A:S21, A:D22, A:S23, A:A24, A:P25, A:S26, A:S27, A:E28, A:E29, A:A31, A:R32, A:L33, A:L34, A:A35, A:E36, A:R37, A:G38, A:F39, A:D40, A:K41, A:P42, A:F50, A:K51, A:T52, A:F53, A:P54, A:V55, A:T56, A:L57, A:S58, A:D59, A:S60, A:F61, A:S62, A:G63, A:P64, A:N70, A:A72, A:D75, A:A76, A:V78, A:G79, A:V80, A:G81, A:L82, A:L83, A:R85 | 1 | HP2  HP2 |
| 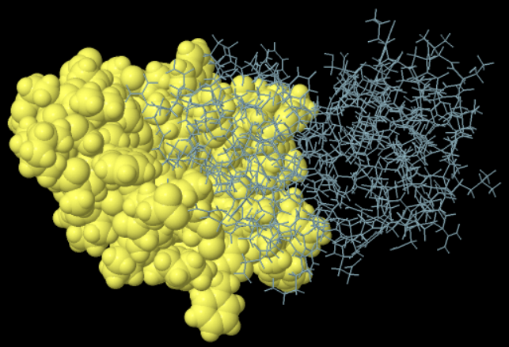 |  | 76 | A:P94, A:A95, A:Y109, A:S110, A:G112, A:F113, A:D114, A:V115, A:S116, A:V117, A:R118, A:S119, A:V120, A:D121, A:P122, A:A123, A:K124, A:P125, A:D126, A:D127, A:Y128, A:G129, A:P130, A:A131, A:V132, A:E133, A:K134, A:G135, A:W136, A:L137, A:T139, A:E141, A:V142, A:K143, A:P144, A:R145, A:E146, A:V147, A:K148, A:D149, A:W150, A:A151, A:K152, A:N153, A:P154, A:E155, A:V156, A:L157, A:K158, A:Q159, A:A160, A:S161, A:L162, A:T163, A:L165, A:Q166, A:Q167, A:I168, A:T169, A:Q170, A:P171, A:Q172, A:S177, A:V179, A:K180, A:P181, A:R182, A:G183, A:E184, A:E185, A:G186, A:Y187, A:K188, A:L189, A:V190, A:N191 | 2 |  |
| 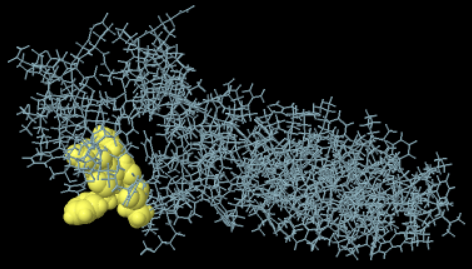 | 0.833 | 5 | A:D9, A:A10, A:F12, A:F13, A:R16 | 1 | HP3  HP3  HP3 |
| 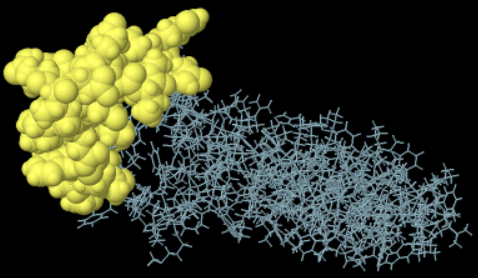 | 0.823 | 29 | A:H17, A:L18, A:T19, A:T20, A:L21, A:P23, A:L24, A:C25, A:P27, A:W28, A:I29, A:F30, A:L31, A:E32, A:S33, A:L34, A:L35, A:Q36, A:Q37, A:Q38, A:I39, A:D40, A:E41, A:A42, A:V43, A:G44, A:P45, A:Q46, A:Q47 | 2 |  |
| 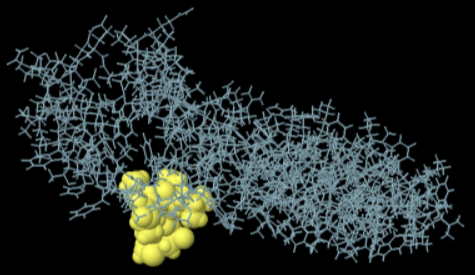 | 0.765 | 6 | A:M1, A:N2, A:P3, A:L4, A:L5, A:P6 | 3 |  |
| 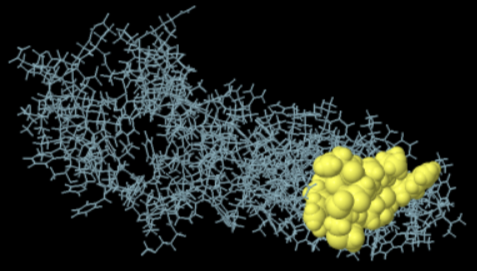 | 0.749 | 11 | A:R206, A:S209, A:L210, A:G211, A:P212, A:A213, A:P214, A:R215, A:N216, A:G217, A:M218 | 4 |  |
| 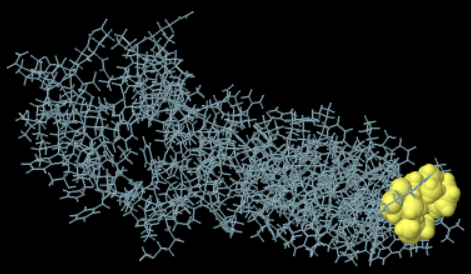 | 0.686 | 4 | A:F195, A:L198, A:L199, A:I202 | 5 |  |
| 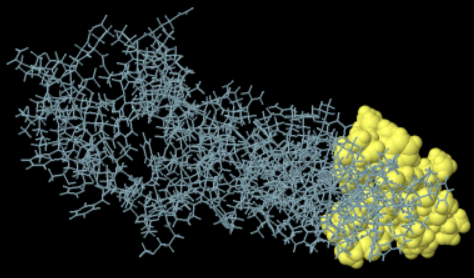 | 0.674 | 25 | A:A97, A:L98, A:L99, A:L102, A:S104, A:L105, A:L175, A:A176, A:D177, A:S178, A:A179, A:G180, A:D181, A:A182, A:V183, A:V184, A:L185, A:R186, A:V187, A:A188, A:L189, A:H190, A:T191, A:L192, A:F196 | 6 |  |
| 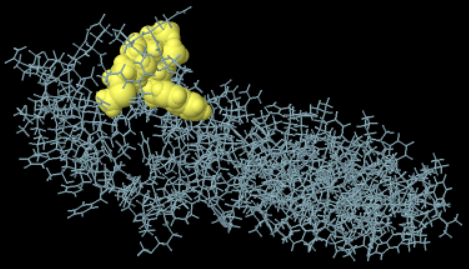 | 0.66 | 6 | A:M48, A:G49, A:A50, A:W51, A:S52, A:L53 | 7 |  |
| 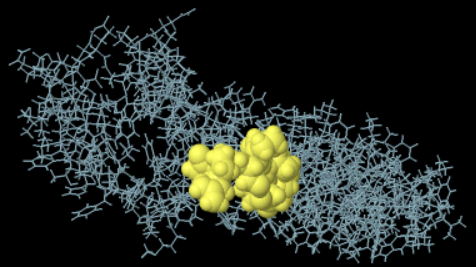 | 0.632 | 7 | A:G136, A:R137, A:S138, A:P139, A:Q141, A:A142, A:E145 | 8 |  |
| 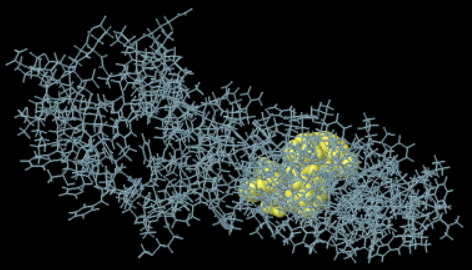 | 0.624 | 9 | A:S87, A:A88, A:A89, A:L90, A:R91, A:L92, A:P94, A:A95, A:F96 | 9 |  |
| 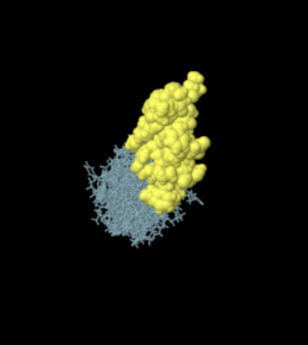 | 0.81 | 33 | A:G111, A:V113, A:A114, A:P115, A:A116, A:S117, A:K118, A:A119, A:D120, A:A121, A:L122, A:E123, A:I124, A:R125, A:A126, A:F127, A:E128, A:V129, A:Q130, A:G131, A:T132, A:D133, A:D134, A:P135, A:Q136, A:R137, A:F138, A:R139, A:Y140, A:K141, A:V142, A:L144, A:S145 | 1 | HP4  HP4  HP4 |
| 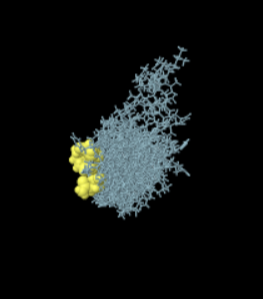 | 0.763 | 3 | A:Q90, A:K93, A:E96 | 2 |  |
| 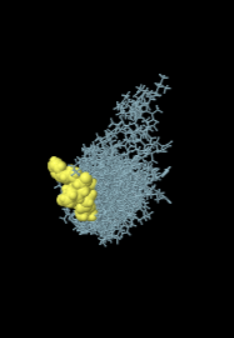 | 0.741 | 5 | A:L94, A:D97, A:Q98, A:F100, A:K101 | 3 |  |
| 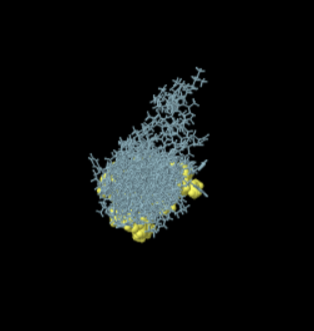 | 0.73 | 44 | A:M1, A:S2, A:G3, A:W4, A:E5, A:L6, A:Q7, A:F8, A:D10, A:P11, A:R12, A:R13, A:A14, A:W15, A:L16, A:V17, A:R18, A:L19, A:G20, A:G22, A:A23, A:L25, A:L26, A:A27, A:P29, A:L30, A:A31, A:F32, A:L33, A:G34, A:S38, A:G40, A:A41, A:A44, A:Q226, A:S227, A:L230, A:Q231, A:R233, A:F234, A:D235, A:P237, A:K238, A:Q239 | 4 |  |
| 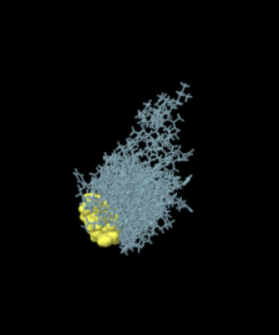 | 0.699 | 6 | A:E78, A:R79, A:Q82, A:Q83, A:E86, A:R89 | 5 |  |
| 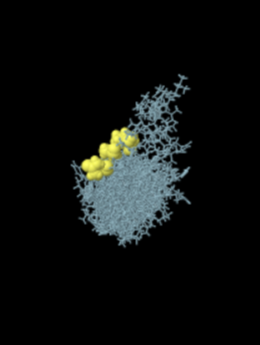 | 0.618 | 3 | A:Q104, A:A107, A:K110 | 6 |  |
| 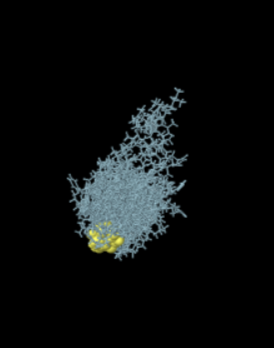 | 0.597 | 3 | A:E72, A:R75, A:S76 | 7 |  |
| 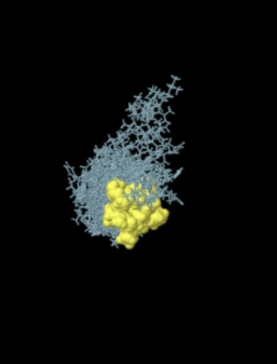 | 0.587 | 14 | A:R146, A:G148, A:R149, A:D150, A:D151, A:R152, A:K153, A:L154, A:D155, A:G156, A:R157, A:L158, A:K159, A:V160 | 8 |  |
| 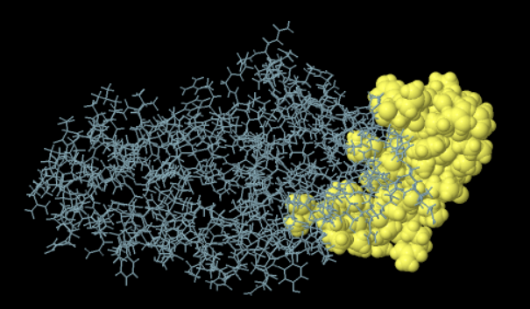 | 0.753 | 44 | A:V142, A:L143, A:P144, A:A145, A:R146, A:E147, A:A148, A:G149, A:A150, A:E151, A:V152, A:L154, A:A155, A:L156, A:V159, A:E200, A:L203, A:L204, A:Y205, A:V206, A:Y207, A:R208, A:D209, A:L210, A:S211, A:D212, A:A213, A:E214, A:L215, A:T216, A:Q217, A:Y218, A:V219, A:N220, A:F221, A:A222, A:E223, A:S224, A:P225, A:A226, A:R228, A:S229, A:Y230, A:Q232 | 1 | HP5  HP5  HP5 |
| 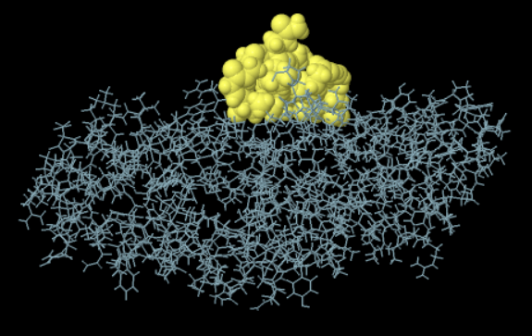 | 0.724 | 13 | A:L175, A:G176, A:G177, A:G178, A:Q179, A:T180, A:G181, A:Q182, A:L183, A:E185, A:G186, A:Q189, A:R190 | 2 |  |
| 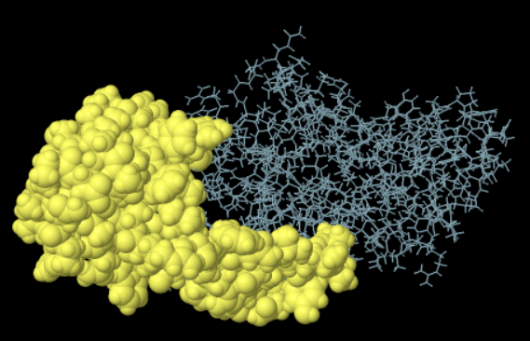 | 0.654 | 72 | A:L9, A:L10, A:L11, A:V12, A:G13, A:L14, A:P15, A:A16, A:L17, A:A18, A:D19, A:P20, A:Y21, A:Q22, A:R23, A:L24, A:Y25, A:Q26, A:A27, A:A28, A:G29, A:W30, A:P31, A:D32, A:D42, A:Q45, A:K46, A:Q49, A:K51, A:L52, A:P53, A:P54, A:A55, A:Y57, A:Q58, A:T59, A:V61, A:D62, A:N65, A:R66, A:F68, A:A69, A:P70, A:A71, A:E72, A:V73, A:Q75, A:R76, A:A77, A:L78, A:N79, A:G80, A:L81, A:R82, A:E83, A:N84, A:L85, A:P86, A:D87, A:S88, A:A89, A:S90, A:P91, A:L92, A:N93, A:F94, A:F95, A:E96, A:S97, A:P98, A:G100, A:R101 | 3 |  |
| 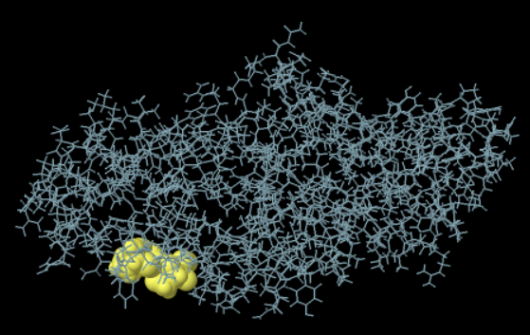 | 0.533 | 3 | A:R34, A:S38, A:D39 | 4 |  |
| 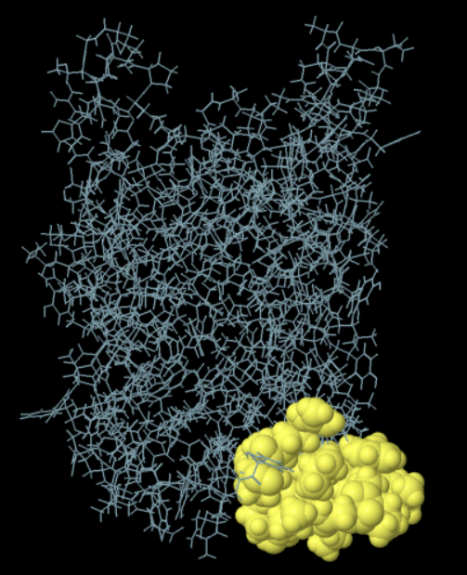 | 0.837 | 26 | A:Y99, A:A100, A:S101, A:R102, A:V103, A:F104, A:D105, A:R106, A:P107, A:L108, A:G109, A:T110, A:G111, A:L112, A:V113, A:K114, A:D115, A:F146, A:N147, A:G148, A:P149, A:G150, A:V151, A:L152, A:R189, A:P190 | 1 | HP6  HP6  HP6 |
| 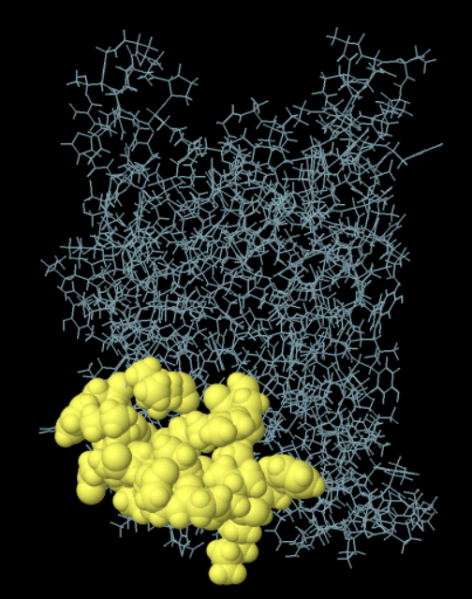 | 0.803 | 17 | A:F191, A:K192, A:V193, A:G194, A:N195, A:H196, A:A197, A:A198, A:D237, A:R239, A:Q240, A:P241, A:N242, A:L243, A:L245, A:F275, A:T276 | 2 |  |
| 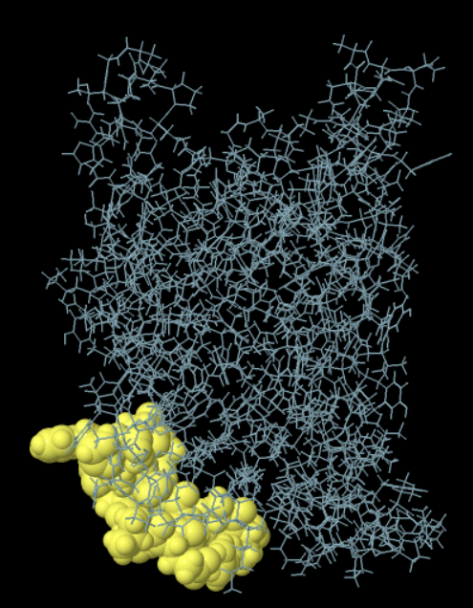 | 0.687 | 14 | A:A17, A:G18, A:E19, A:S20, A:M21, A:A22, A:L23, A:E24, A:W25, A:M26, A:N28, A:Y60, A:R61, A:F277 | 3 |  |
| 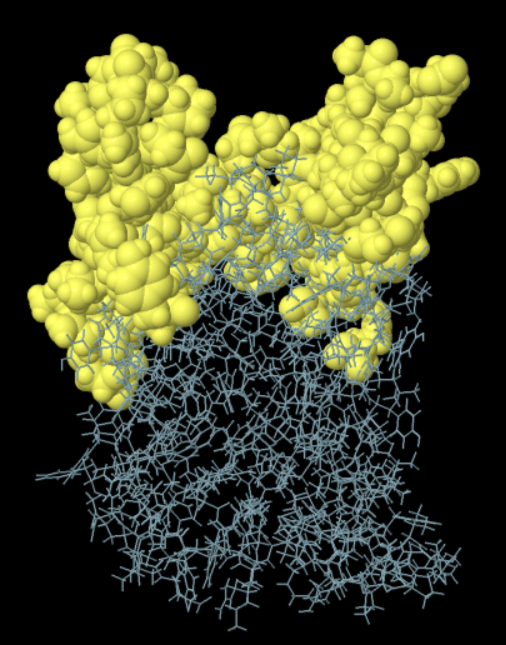 | 0.66 | 82 | A:M1, A:K2, A:K3, A:M4, A:M5, A:Q6, A:A9, A:A10, A:Y34, A:R50, A:L73, A:S74, A:D75, A:N76, A:R77, A:D78, A:P79, A:R80, A:K81, A:G82, A:T83, A:D84, A:H85, A:G86, A:G87, A:S88, A:E89, A:R126, A:N127, A:N128, A:N129, A:L130, A:A131, A:S132, A:A133, A:K134, A:R160, A:K162, A:H164, A:F165, A:G166, A:V167, A:P168, A:G169, A:A170, A:R171, A:H172, A:P173, A:D174, A:H175, A:T176, A:F177, A:D178, A:T179, A:K210, A:G211, A:E212, A:D213, A:Y214, A:N215, A:D216, A:K217, A:D218, A:T219, A:A220, A:A221, A:E222, A:T223, A:L224, A:V225, A:R226, A:T227, A:A228, A:N254, A:V258, A:D259, A:G260, A:G261, A:T262, A:G263, A:S264, A:R265 | 4 |  |
| 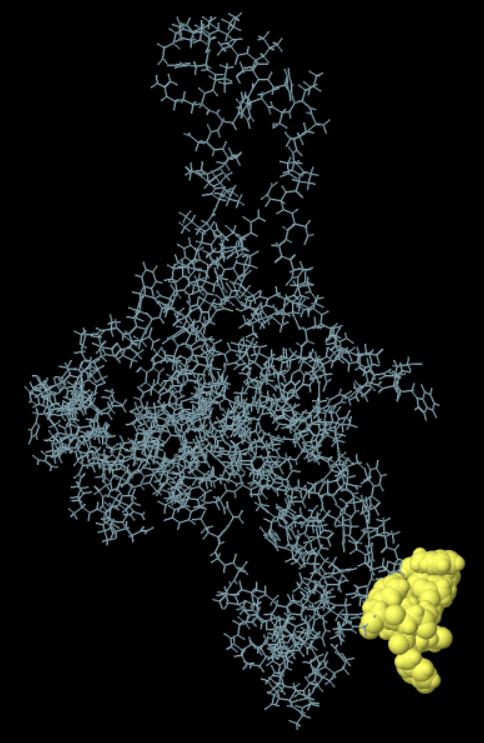 | 0.891 | 10 | A:F275, A:E276, A:E277, A:Y278, A:L279, A:G280, A:G281, A:Y282, A:N283, A:F284 | 1 | HP7  HP7  HP7  HP7  HP7  HP7  HP7 |
| 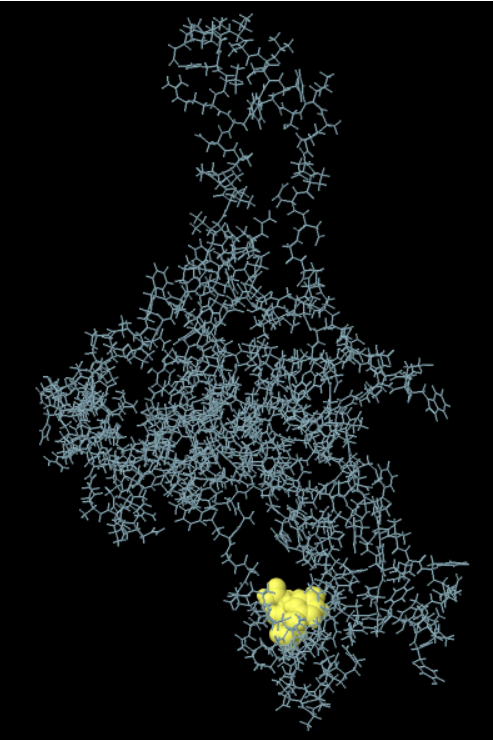 | 0.855 | 3 | A:N2, A:A3, A:Y4 | 2 |  |
| 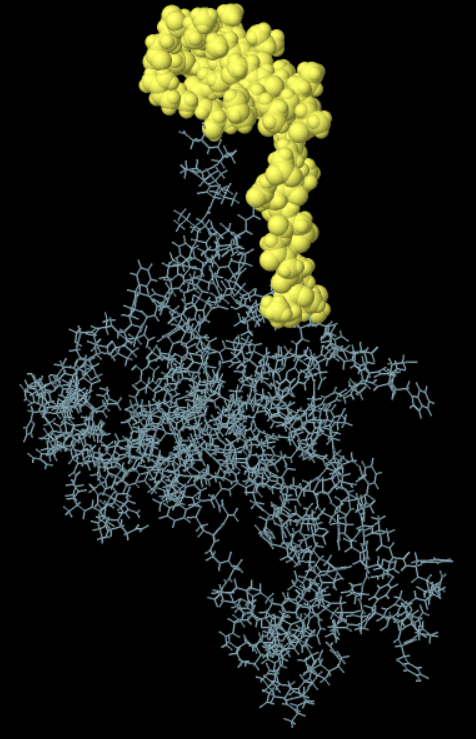 | 0.838 | 27 | A:T41, A:Y57, A:R58, A:V59, A:H60, A:G61, A:P62, A:E63, A:I64, A:V65, A:A66, A:L67, A:S68, A:F69, A:F70, A:S71, A:L72, A:A73, A:P74, A:D75, A:Q76, A:N77, A:D78, A:G79, A:G80, A:P81, A:T82 | 3 |  |
| 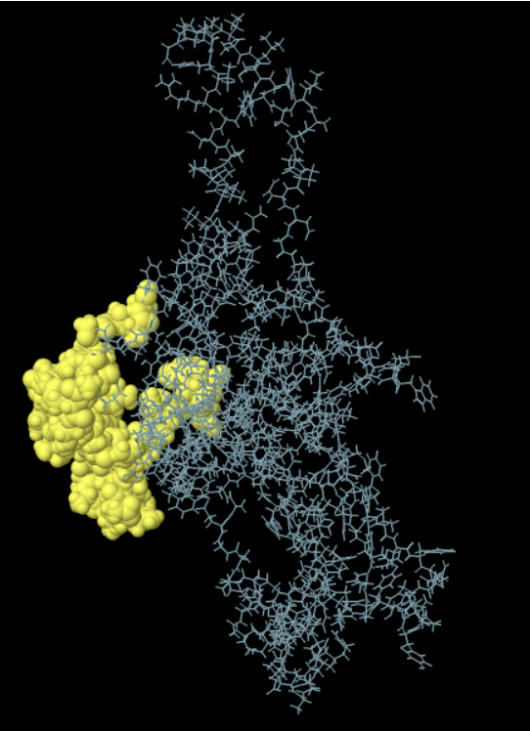 | 0.705 | 51 | A:L156, A:A157, A:Q158, A:T159, A:E160, A:P161, A:P162, A:W164, A:L165, A:E166, A:A167, A:G168, A:S169, A:A170, A:A171, A:S172, A:R186, A:A187, A:G189, A:G190, A:R191, A:P192, A:A193, A:A194, A:G195, A:H196, A:Q197, A:Q198, A:R199, A:Y200, A:P201, A:L202, A:R203, A:W204, A:T205, A:A206, A:R207, A:A208, A:Q209, A:D210, A:P211, A:N212, A:A213, A:G214, A:V215, A:V216, A:P217, A:S218, A:E219, A:Y220, A:D222 | 4 |  |
| 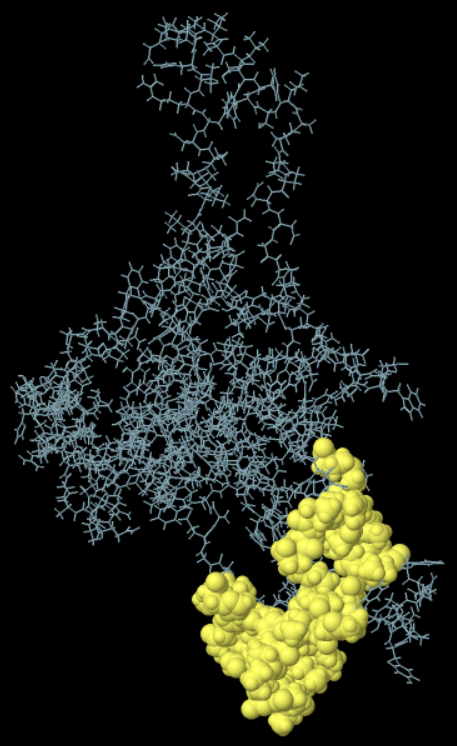 | 0.663 | 42 | A:D5, A:I6, A:E7, A:I8, A:V9, A:N10, A:D11, A:A12, A:A13, A:H14, A:L91, A:I92, A:D93, A:P94, A:P95, A:A96, A:Q97, A:P98, A:P99, A:A100, A:E101, A:A102, A:E103, A:L104, A:R105, A:P106, A:F107, A:W108, A:Y272, A:V273, A:E274, A:G285, A:G286, A:G287, A:N288, A:A289, A:Q290, A:L291, A:D292, A:F293, A:R294, A:M296 | 5 |  |
| 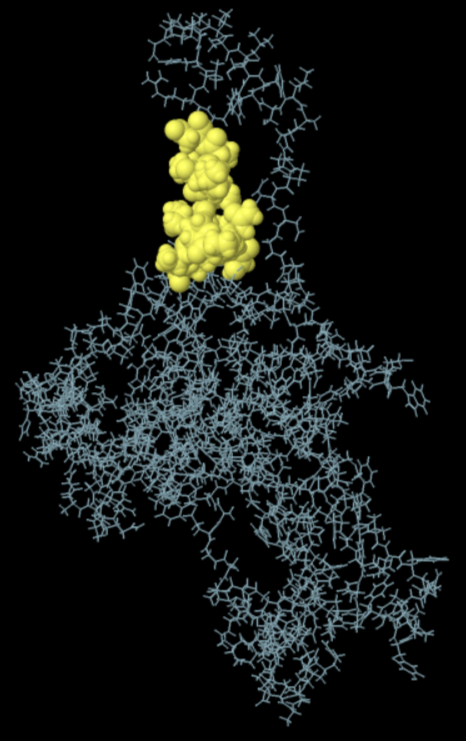 | 0.599 | 13 | A:T50, A:L51, A:L52, A:L53, A:P54, A:E55, A:D56, A:C144, A:Q145, A:P146, A:P147, A:A148, A:L149 | 6 |  |
| 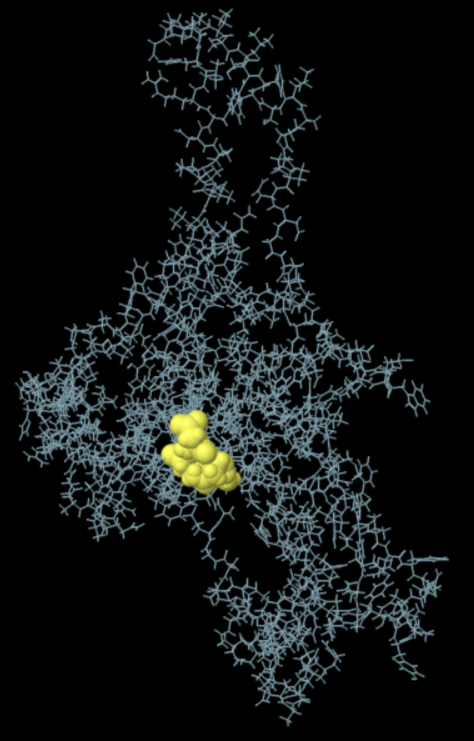 | 0.589 | 3 | A:R238, A:D239, A:N240 | 7 |  |
| 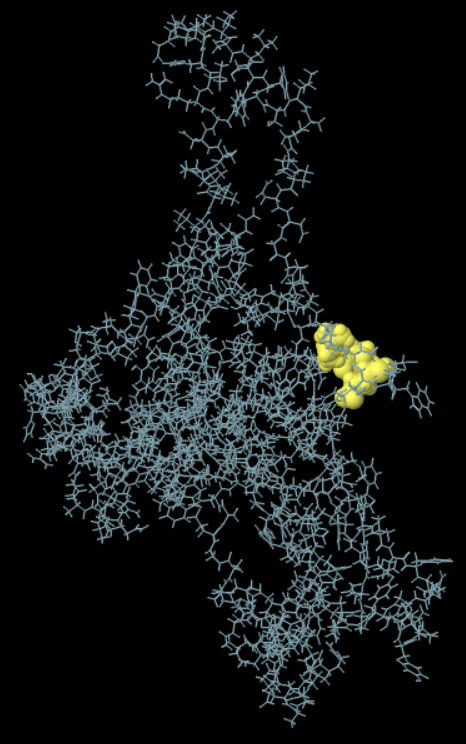 | 0.553 | 3 | A:R31, A:P32, A:E33 | 8 |  |
| 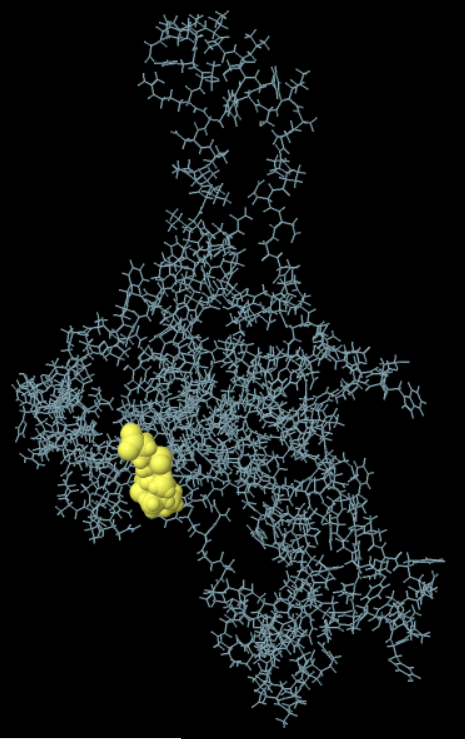 | 0.55 | 4 | A:G234, A:K235, A:I236, A:E237 | 9 |  |
| 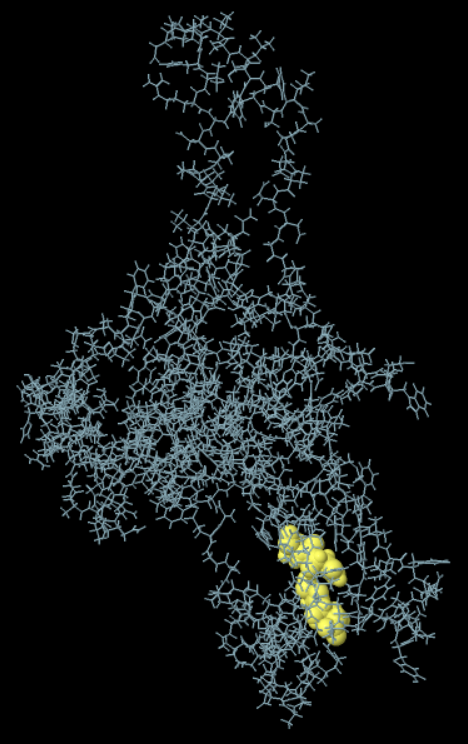 | 0.547 | 4 | A:E109, A:A110, A:N113, A:S114 | 10 |  |
| 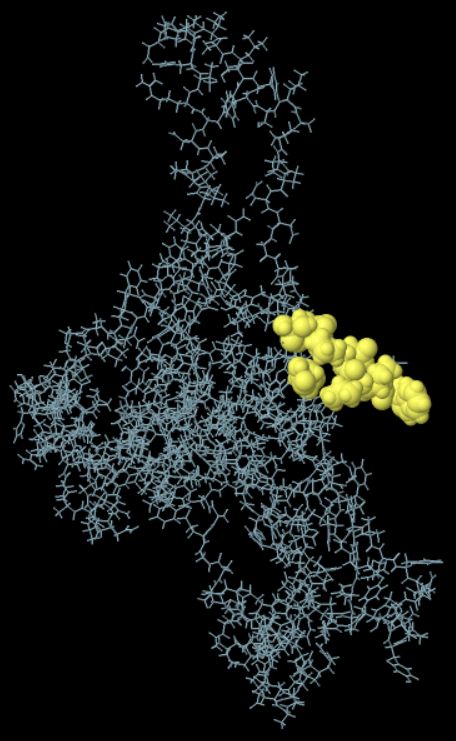 | 0.53 | 8 | A:W35, A:P36, A:L37, A:D38, A:P39, A:D40, A:S83, A:D85 | 11 |  |
| 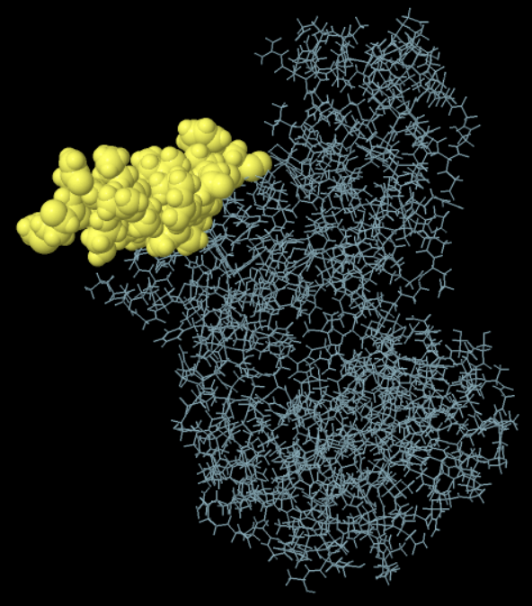 | 0.856 | 18 | A:M1, A:N2, A:N3, A:S4, A:N5, A:L6, A:A7, A:A8, A:A9, A:R10, A:N11, A:L12, A:I13, A:Q14, A:V15, A:V16, A:T17, A:W20 | 1 | HP8  HP8  HP8  HP8 |
| 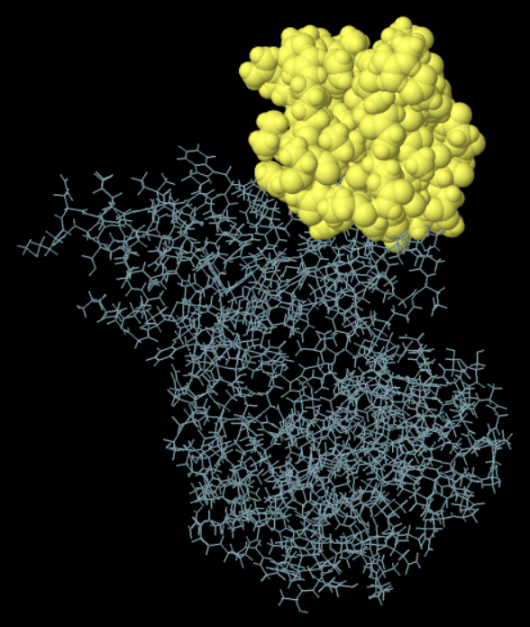 | 0.771 | 49 | A:L31, A:G32, A:L36, A:I37, A:E38, A:S39, A:G40, A:I41, A:D42, A:S43, A:D44, A:E45, A:T46, A:L47, A:A48, A:A49, A:A50, A:V51, A:G52, A:S53, A:D54, A:A55, A:E56, A:R57, A:I58, A:H59, A:R60, A:L61, A:M62, A:R63, A:L64, A:V66, A:A67, A:F68, A:E69, A:I70, A:F71, A:Q72, A:G73, A:D74, A:T75, A:R76, A:D77, A:G78, A:Y79, A:A80, A:N81, A:T82, A:R89 | 2 |  |
| 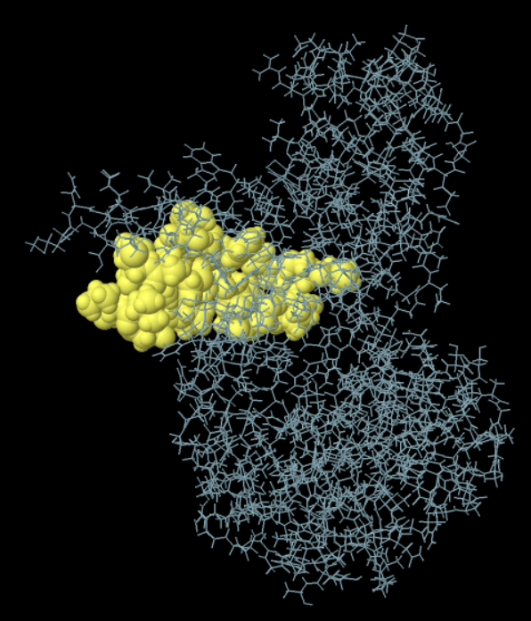 | 0.721 | 25 | A:T111, A:P112, A:A113, A:C114, A:E115, A:A116, A:L117, A:L118, A:S119, A:G120, A:T121, A:P122, A:G123, A:E125, A:L126, A:A127, A:F128, A:G129, A:E130, A:D131, A:S134, A:K137, A:R138, A:C139, A:P140 | 3 |  |
| 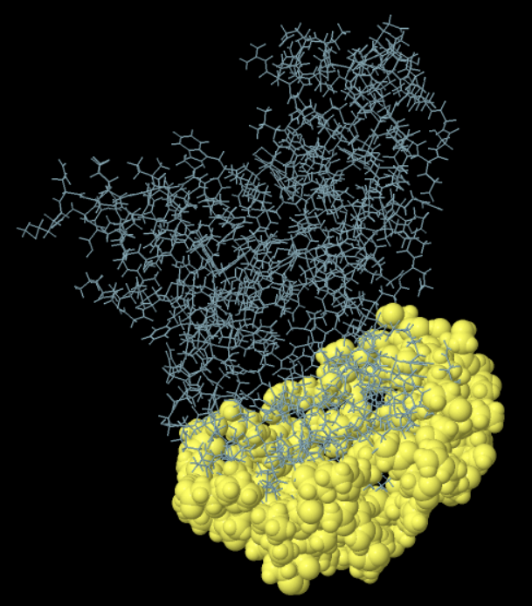 | 0.651 | 76 | A:L164, A:D165, A:F166, A:R167, A:G168, A:R169, A:S170, A:A188, A:E189, A:P190, A:S191, A:A192, A:R193, A:L197, A:R199, A:E200, A:G201, A:G204, A:R207, A:D208, A:L210, A:S211, A:S212, A:L213, A:L214, A:A215, A:G216, A:E217, A:R218, A:V219, A:S220, A:L221, A:V222, A:G223, A:G224, A:D225, A:L227, A:Q228, A:E229, A:V230, A:P231, A:S232, A:N233, A:G234, A:D235, A:L246, A:D247, A:E248, A:A249, A:L252, A:R253, A:G256, A:N257, A:R259, A:E260, A:A261, A:M262, A:A263, A:G264, A:D265, A:G266, A:R267, A:V306, A:D307, A:G310, A:R311, A:G312, A:G313, A:F314, A:A315, A:V316, A:E317, A:R318, A:A332, A:R333, A:A334 | 4 |  |
| 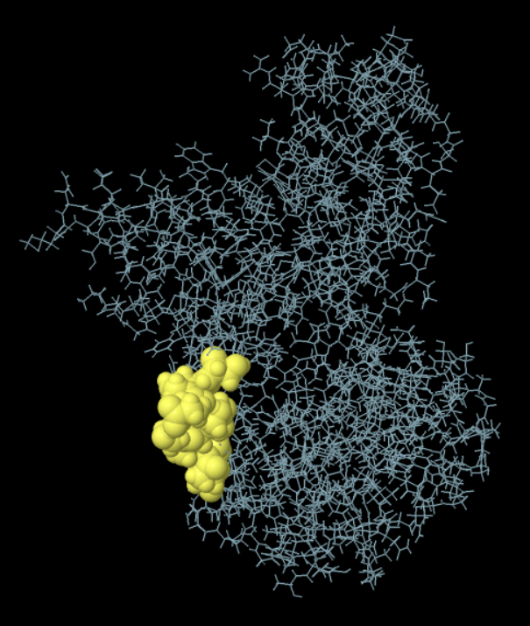 | 0.597 | 9 | A:I275, A:S276, A:A277, A:S278, A:E279, A:P280, A:S281, A:T301, A:E303 | 5 |  |
| 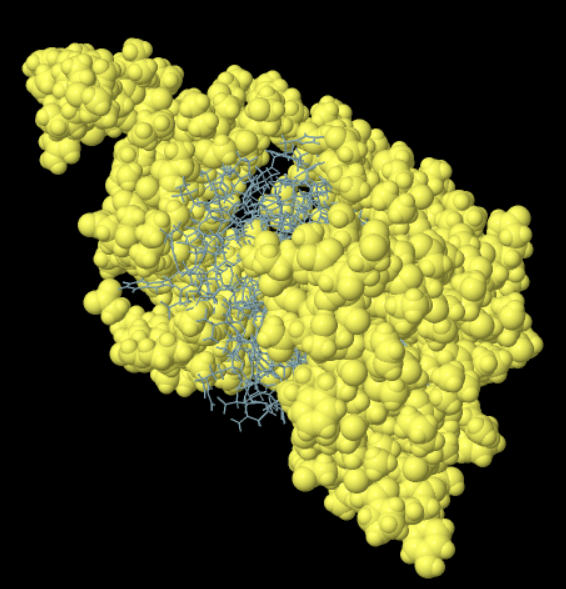 | 0.668 | 209 | A:V1, A:S2, A:R3, A:R4, A:P5, A:W6, A:G7, A:V8, A:L9, A:L10, A:G11, A:C12, A:C13, A:L14, A:L15, A:A16, A:A17, A:S18, A:A19, A:A20, A:Q21, A:A22, A:E23, A:S24, A:I26, A:D27, A:R28, A:Q30, A:R31, A:E32, A:L33, A:A34, A:L35, A:L36, A:R37, A:E38, A:G39, A:W40, A:Y41, A:R42, A:Q43, A:V44, A:P45, A:P46, A:A47, A:V49, A:L51, A:P52, A:M53, A:R54, A:T55, A:A56, A:G57, A:A59, A:D60, A:S61, A:G62, A:Q63, A:D64, A:G65, A:V66, A:G67, A:I68, A:L69, A:L70, A:R71, A:N72, A:V73, A:R78, A:G79, A:N80, A:I81, A:G82, A:K89, A:P94, A:N95, A:R96, A:A97, A:D104, A:R105, A:P106, A:Q107, A:D108, A:L119, A:L120, A:E131, A:Y136, A:D137, A:G138, A:T139, A:M140, A:L141, A:G151, A:R152, A:L153, A:E154, A:V155, A:E156, A:G157, A:R158, A:V159, A:R160, A:P161, A:L162, A:G163, A:V164, A:G165, A:P166, A:S204, A:L205, A:V206, A:G207, A:L208, A:P209, A:M210, A:S211, A:A212, A:L213, A:R229, A:F236, A:P237, A:L238, A:L239, A:V245, A:Q246, A:D252, A:Q253, A:G254, A:F259, A:S260, A:Q261, A:A262, A:P263, A:G264, A:Q265, A:P266, A:S267, A:S271, A:P272, A:P273, A:A274, A:A275, A:A276, A:G277, A:L283, A:Q284, A:S285, A:G286, A:D287, A:K289, A:I290, A:F291, A:E292, A:I293, A:L294, A:I295, A:T296, A:Y297, A:A298, A:Q299, A:V300, A:L301, A:L302, A:K303, A:T304, A:E305, A:T306, A:P307, A:D308, A:Q309, A:V310, A:L311, A:T312, A:F313, A:N314, A:L315, A:Q316, A:D317, A:R319, A:K320, A:V321, A:L322, A:A323, A:T324, A:G325, A:T326, A:T327, A:Q328, A:V329, A:N330, A:E331, A:D332, A:G333, A:T334, A:F335, A:F336, A:M337, A:Q338, A:V339, A:P340, A:P341, A:Y342, A:A343, A:N344, A:P345, A:L346 | 1 | HP9  HP9  HP9 |
| 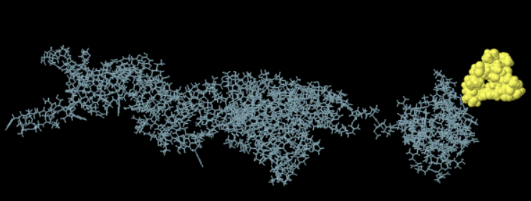 | 0.942 | 14 | A:E341, A:R342, A:L343, A:D344, A:A345, A:L346, A:E347, A:P348, A:I349, A:I350, A:R351, A:R352, A:V353, A:E354 | 1 | HP10  HP10  HP10  HP10 |
| 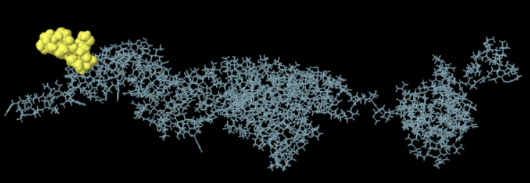 | 0.808 | 9 | A:L46, A:M47, A:R48, A:G49, A:D50, A:Q51, A:P52, A:L53, A:L54 | 2 |  |
| 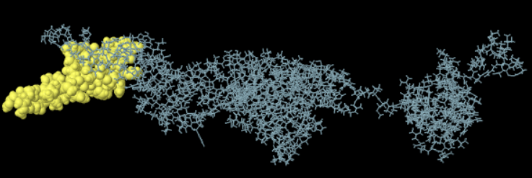 | 0.802 | 39 | A:M1, A:H2, A:T3, A:L4, A:Y5, A:W6, A:R7, A:D8, A:L9, A:E10, A:S11, A:P12, A:A13, A:N14, A:G15, A:E16, A:W17, A:L18, A:G19, A:L20, A:A21, A:V22, A:Q23, A:V24, A:E25, A:G26, A:A27, A:H28, A:R29, A:A30, A:P31, A:S32, A:F33, A:L34, A:H35, A:L36, A:Q37, A:G39, A:G40 | 3 |  |
| 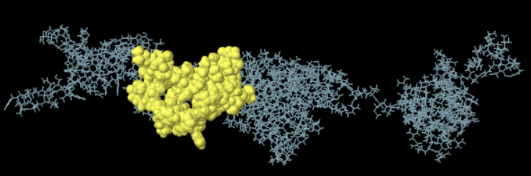 | 0.683 | 47 | A:P83, A:I84, A:D85, A:S86, A:P87, A:T88, A:L89, A:E90, A:A91, A:L92, A:R93, A:G94, A:L95, A:G97, A:E98, A:A99, A:R100, A:Y101, A:K102, A:A103, A:W104, A:S105, A:R106, A:Y107, A:F108, A:V109, A:E110, A:T111, A:L112, A:R113, A:T114, A:S115, A:P116, A:G117, A:S118, A:C119, A:L120, A:E121, A:A122, A:G123, A:R124, A:L126, A:L127, A:R128, A:L129, A:S130, A:L131 | 4 |  |
| 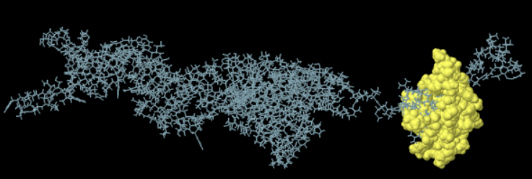 | 0.646 | 60 | A:D269, A:T270, A:A271, A:Q272, A:R273, A:E274, A:A286, A:P287, A:V288, A:R289, A:R290, A:G291, A:R292, A:R293, A:P294, A:L295, A:D296, A:S297, A:E298, A:Q299, A:L300, A:N301, A:Q302, A:V303, A:L304, A:L305, A:R306, A:L307, A:F308, A:D309, A:D310, A:P312, A:W313, A:P314, A:R315, A:V316, A:L317, A:T318, A:R319, A:A320, A:R321, A:A322, A:V323, A:L324, A:K325, A:E326, A:E327, A:Q328, A:W329, A:C330, A:A331, A:E332, A:V333, A:R334, A:D335, A:W336, A:L337, A:A338, A:A339, A:R340 | 5 |  |
| 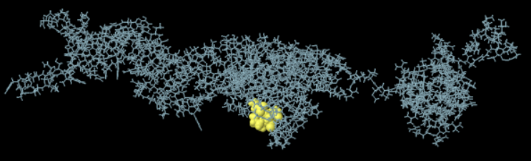 | 0.592 | 5 | A:D204, A:E205, A:G206, A:R207, A:K209 | 6 |  |
| 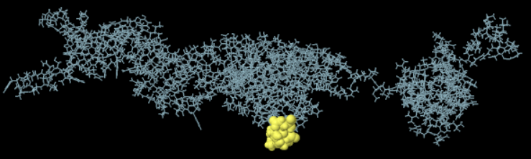 | 0.588 | 4 | A:K213, A:R216, A:E217, A:E219 | 7 |  |
| 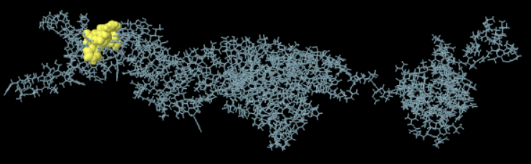 | 0.561 | 4 | A:R45, A:W55, A:A56, A:T57 | 8 |  |
| 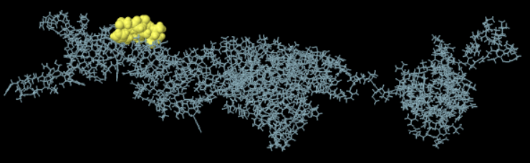 | 0.553 | 5 | A:Y61, A:H62, A:D63, A:G64, A:I65 | 9 |  |
| 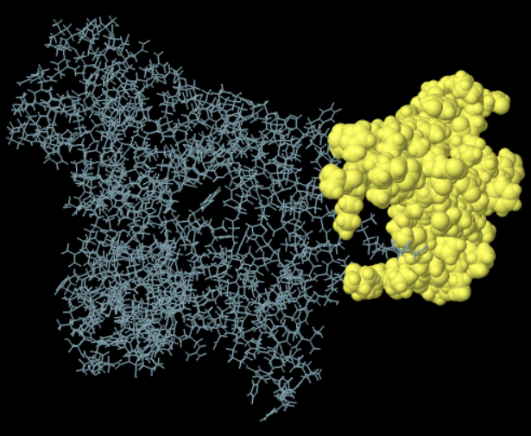 | 0.794 | 65 | A:R5, A:K6, A:H7, A:L8, A:I9, A:Y10, A:L11, A:V12, A:Y13, A:G14, A:R15, A:Q16, A:T17, A:Y18, A:H19, A:Q20, A:E21, A:A22, A:L23, A:F24, A:S25, A:I26, A:A27, A:S28, A:A81, A:K82, A:H83, A:V84, A:A85, A:L86, A:R87, A:D88, A:A89, A:L90, A:E91, A:D92, A:A93, A:D94, A:Q95, A:A96, A:L97, A:L98, A:I99, A:D100, A:T101, A:D102, A:T103, A:F104, A:F105, A:H106, A:S107, A:S108, A:P109, A:L110, A:R111, A:L112, A:F113, A:D114, A:R115, A:I116, A:Q117, A:P118, A:G119, A:S120, A:L121 | 1 | HP11  HP11  HP11  HP11 |
| 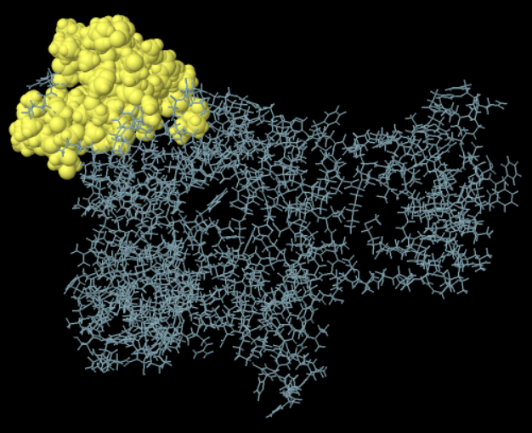 | 0.702 | 44 | A:G271, A:A309, A:A310, A:N312, A:E315, A:R316, A:Y317, A:P318, A:Q319, A:E320, A:P321, A:I322, A:A323, A:E324, A:Q325, A:I326, A:E327, A:D328, A:W329, A:L330, A:N331, A:H332, A:S333, A:L334, A:N337, A:L338, A:L339, A:G340, A:S341, A:R342, A:A343, A:N344, A:E345, A:I346, A:R347, A:Q348, A:H349, A:L350, A:R351, A:Q352, A:R353, A:D354, A:L355, A:L356 | 2 |  |
| 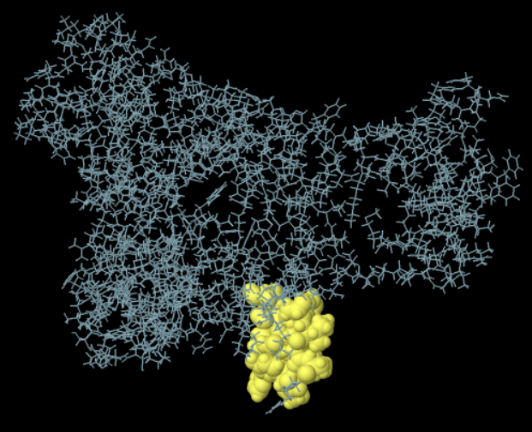 | 0.692 | 14 | A:A125, A:I126, A:G127, A:A128, A:Y130, A:G131, A:E132, A:H133, A:R134, A:N135, A:F136, A:P137, A:L138, A:D141 | 3 |  |
| 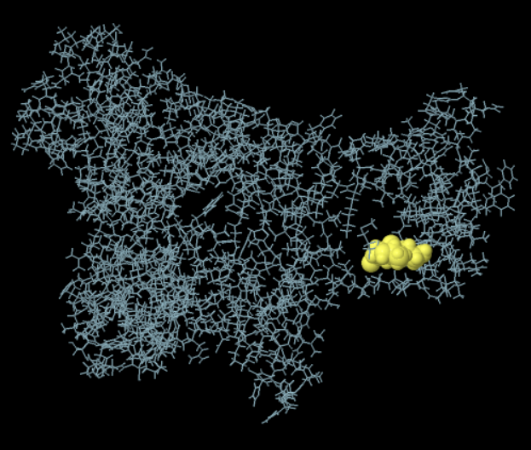 | 0.647 | 3 | A:A2, A:N3, A:S4 | 4 |  |
| 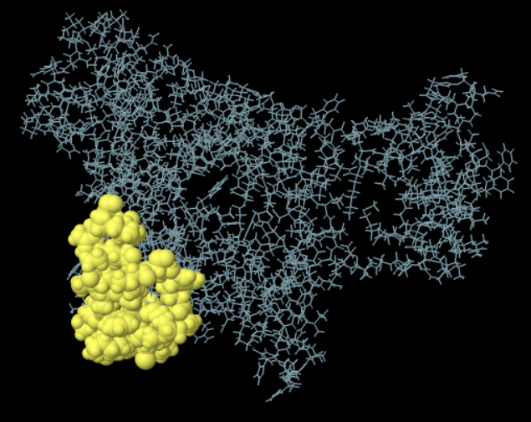 | 0.612 | 29 | A:A202, A:A203, A:H204, A:K205, A:R227, A:A228, A:K229, A:A232, A:W233, A:L234, A:C235, A:K236, A:H237, A:G238, A:S239, A:S240, A:L241, A:L242, A:S243, A:A245, A:A246, A:D249, A:T250, A:K252, A:V253, A:T254, A:L257, A:P258, A:R259 | 5 |  |
| 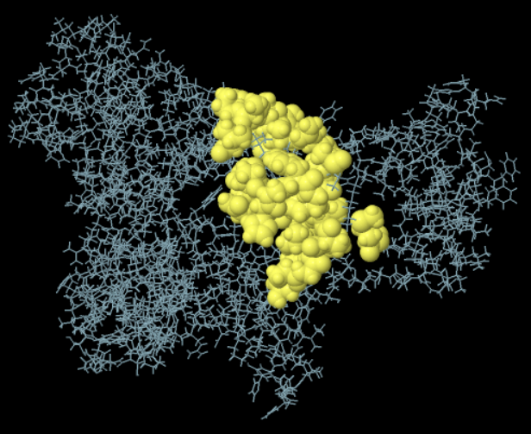 | 0.591 | 24 | A:M1, A:Y46, A:P52, A:Y53, A:A54, A:G55, A:L56, A:P57, A:V58, A:Q59, A:V63, A:T64, A:A65, A:E66, A:V69, A:C72, A:Q73, A:P74, A:H75, A:G76, A:Y77, A:H78, A:F79, A:R80 | 6 |  |
| 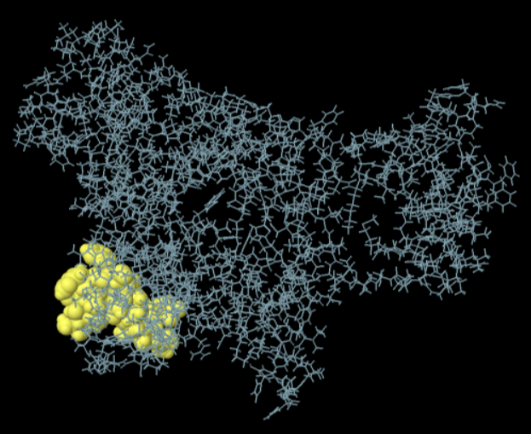 | 0.584 | 14 | A:R206, A:L207, A:E208, A:L209, A:V210, A:G211, A:C212, A:T213, A:D214, A:L215, A:I216, A:H217, A:H218, A:Y219 | 7 |  |
| 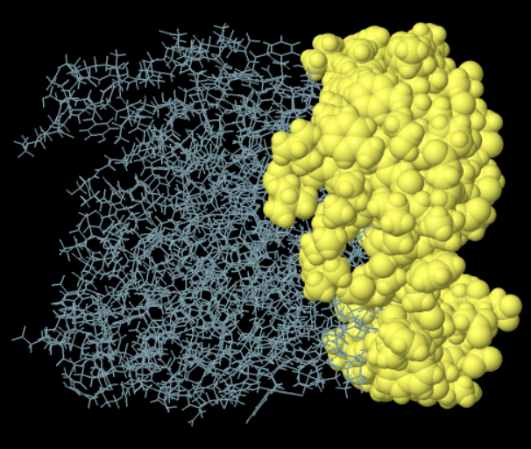 | 0.787 | 105 | A:A22, A:D23, A:E24, A:G25, A:E26, A:A27, A:K28, A:E29, A:G30, A:F31, A:I32, A:E33, A:G34, A:S35, A:S36, A:L37, A:L39, A:F69, A:Q70, A:S71, A:G72, A:Y73, A:T74, A:P75, A:G76, A:V77, A:V78, A:G79, A:F80, A:G81, A:V82, A:L130, A:K131, A:I132, A:R133, A:A134, A:F135, A:D136, A:T137, A:L139, A:L141, A:L168, A:N170, A:N171, A:S172, A:F173, A:E174, A:D175, A:L176, A:W220, A:G221, A:G222, A:I223, A:E224, A:G225, A:F226, A:Y248, A:T249, A:Y250, A:E251, A:I252, A:D253, A:D254, A:N255, A:W256, A:S257, A:L258, A:P260, A:F287, A:A288, A:V289, A:G290, A:Y291, A:R292, A:Q293, A:H294, A:T295, A:V296, A:Y343, A:D344, A:F345, A:V346, A:A347, A:L348, A:G349, A:L350, A:P351, A:G352, A:L353, A:Y398, A:V399, A:V400, A:Q401, A:G402, A:G403, A:P404, A:A405, A:K406, A:D407, A:L408, A:Y439, A:I441, A:D442, A:V443, A:F444 | 1 | HP12  HP12  HP12 |
| 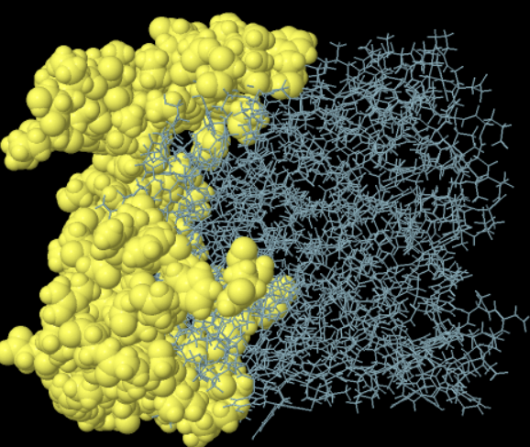 | 0.663 | 121 | A:Y45, A:N47, A:D49, A:R50, A:R51, A:H52, A:A53, A:S54, A:G55, A:H56, A:D57, A:K59, A:W61, A:Q63, A:F65, A:L88, A:L90, A:K91, A:L92, A:D93, A:G94, A:G95, A:G96, A:G97, A:T98, A:G99, A:G100, A:L104, A:P105, A:I106, A:T107, A:A108, A:P109, A:S110, A:K111, A:E112, A:G113, A:Y114, A:E115, A:S116, A:G117, A:K118, A:A119, A:P120, A:D121, A:E122, A:S124, A:Q182, A:R196, A:G202, A:L204, A:P205, A:G206, A:D207, A:R208, A:D209, A:S210, A:H211, A:H212, A:Q236, A:N237, A:V238, A:K266, A:V268, A:D269, A:S270, A:G271, A:D272, A:S273, A:L274, A:L275, A:G276, A:R277, A:I278, A:N304, A:G305, A:N306, A:T307, A:P332, A:N333, A:E334, A:K335, A:Y359, A:R361, A:K363, A:L364, A:D365, A:L366, A:T367, A:R368, A:V369, A:D370, A:P371, A:D372, A:S373, A:P374, A:Y376, A:G377, A:G378, A:W379, A:Y380, A:S381, A:A382, A:D383, A:G384, A:K385, A:N386, A:A387, A:K388, A:W390, A:R418, A:G419, A:T420, A:G421, A:G422, A:Y423, A:S424, A:A425, A:V426, A:N428, A:D429 | 2 |  |
| 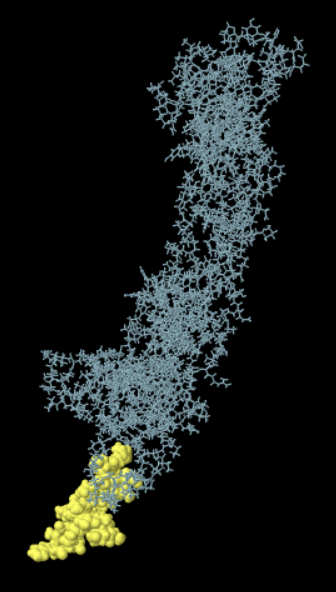 | 0.834 | 33 | A:A46, A:G47, A:N48, A:A49, A:D50, A:G51, A:S52, A:I53, A:P54, A:A55, A:W56, A:D57, A:G58, A:G59, A:L60, A:A61, A:T62, A:N63, A:A64, A:G65, A:S66, A:V67, A:D68, A:S69, A:R70, A:G71, A:F72, A:L73, A:A74, A:N75, A:P76, A:Y77, A:A78 | 1 | HP13  HP13  HP13  HP13  HP13 |
| 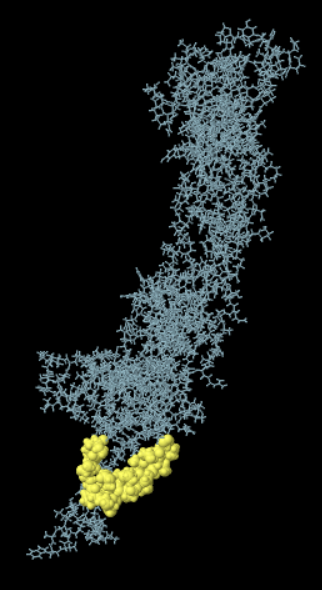 | 0.8 | 21 | A:R122, A:S123, A:A124, A:T125, A:V126, A:P127, A:A128, A:A129, A:V130, A:Q131, A:E132, A:A133, A:A134, A:K135, A:R136, A:N137, A:A138, A:T139, A:T140, A:T141, A:K142 | 2 |  |
| 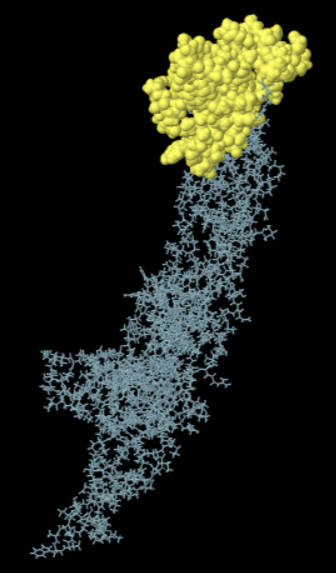 | 0.797 | 77 | A:T373, A:Q375, A:A376, A:A377, A:E378, A:I379, A:D380, A:H381, A:Y382, A:D383, A:G384, A:R385, A:G386, A:T387, A:L388, A:W389, A:R390, A:V391, A:A392, A:E393, A:A394, A:H395, A:A396, A:E397, A:Q398, A:Y399, A:K402, A:Q403, A:W406, A:Y407, A:A408, A:V409, A:E410, A:T411, A:L412, A:Y413, A:D414, A:L415, A:L416, A:S417, A:G418, A:R419, A:Y420, A:L421, A:A422, A:L423, A:G424, A:M425, A:K426, A:N427, A:E428, A:E429, A:K430, A:Q431, A:A432, A:Y433, A:D434, A:F435, A:N436, A:Y437, A:S438, A:A439, A:S440, A:E441, A:S442, A:D443, A:Y444, A:T445, A:P446, A:A447, A:A448, A:L449, A:R450, A:Q451, A:E452, A:G453, A:V454 | 3 |  |
| 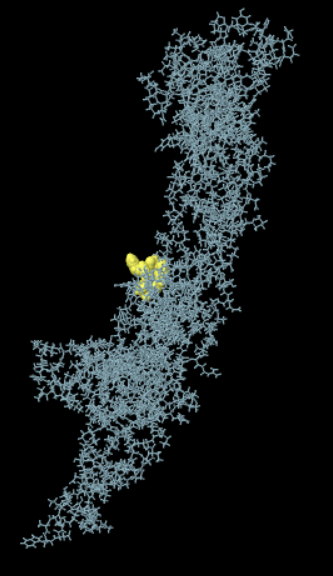 | 0.69 | 5 | A:F201, A:Q202, A:D203, A:A204, A:F205 | 4 |  |
| 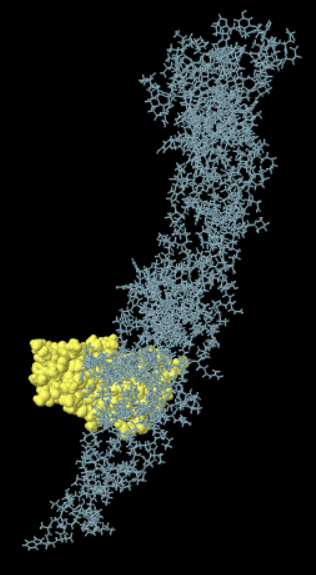 | 0.65 | 52 | A:M1, A:K2, A:T3, A:T4, A:K5, A:I6, A:L7, A:L8, A:H9, A:T10, A:G11, A:V12, A:L13, A:A14, A:L15, A:L17, A:L18, A:Q21, A:V22, A:M23, A:A24, A:A25, A:V26, A:S27, A:A28, A:A31, A:L34, A:G35, A:T36, A:S37, A:L38, A:T39, A:P40, A:L41, A:E80, A:Q81, A:P82, A:L83, A:F84, A:T85, A:I86, A:T87, A:A88, A:Q89, A:N90, A:V91, A:D92, A:Q93, A:Y94, A:D96, A:K97, A:L98 | 5 |  |
| 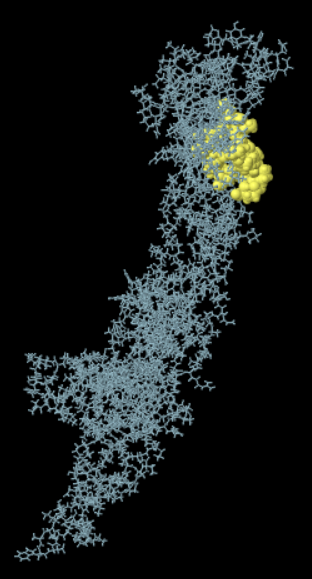 | 0.613 | 18 | A:K303, A:L304, A:E305, A:G306, A:K307, A:E309, A:V330, A:A332, A:G333, A:H334, A:I335, A:N336, A:Q337, A:D338, A:L339, A:T340, A:R341, A:Y342 | 6 |  |
| 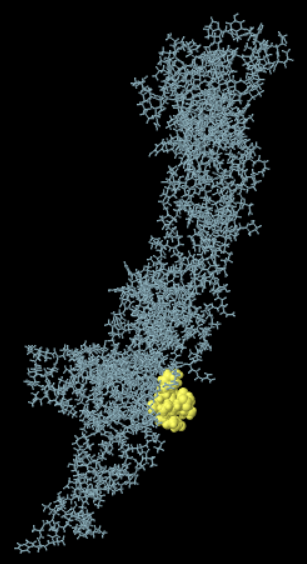 | 0.591 | 7 | A:R108, A:Y109, A:P110, A:D111, A:T112, A:Y113, A:K114 | 7 |  |
| 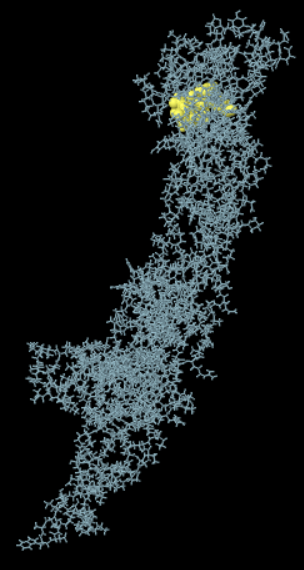 | 0.533 | 9 | A:M292, A:Y293, A:N294, A:G295, A:A296, A:P297, A:D298, A:R299, A:D301 | 8 |  |
| 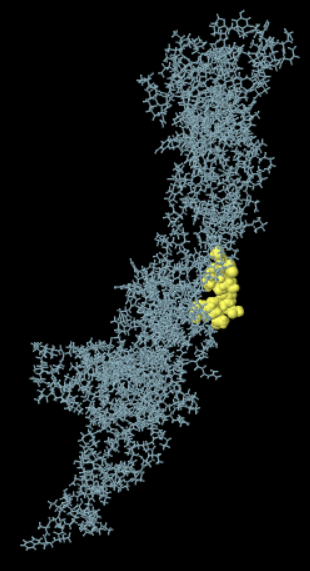 | 0.548 | 9 | A:L258, A:A269, A:P270, A:Q271, A:V272, A:S273, A:Y274, A:D275, A:G276 | 9 |  |
| 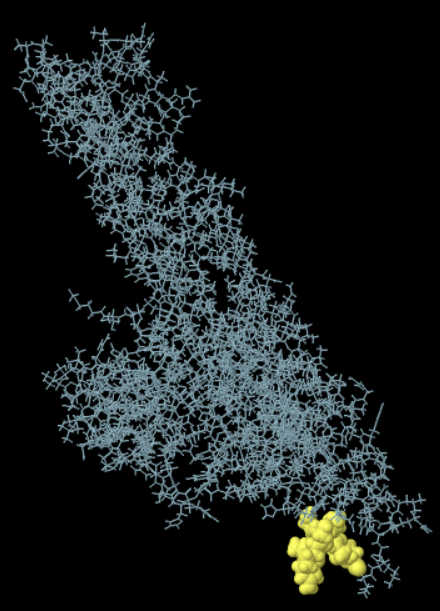 | 0.96 | 7 | A:G356, A:S357, A:R358, A:L359, A:L360, A:G361, A:K362 | 1 | HP14  HP14  HP14  HP14 |
| 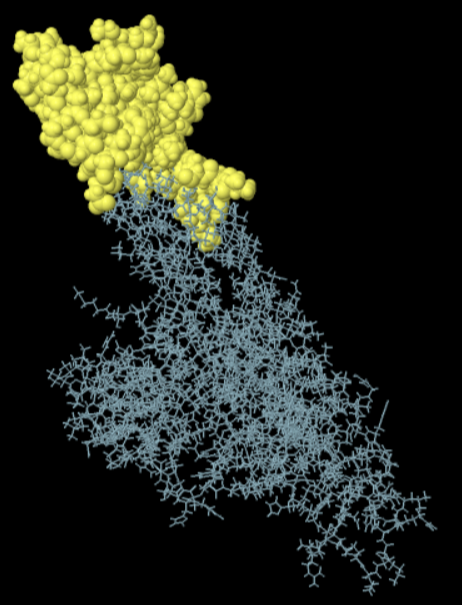 | 0.776 | 78 | A:R204, A:P205, A:Q206, A:L207, A:D208, A:R209, A:L210, A:I211, A:A212, A:D213, A:H214, A:E215, A:T216, A:Q217, A:A218, A:A219, A:A220, A:R221, A:L222, A:R223, A:A224, A:G225, A:Q226, A:R227, A:L228, A:I229, A:A230, A:E231, A:L232, A:L233, A:V234, A:D235, A:A237, A:A238, A:R241, A:L242, A:V243, A:P244, A:L328, A:L329, A:V330, A:R421, A:R424, A:A425, A:H426, A:P427, A:E428, A:W429, A:S430, A:S431, A:L432, A:N433, A:G434, A:R435, A:R436, A:V437, A:G438, A:G439, A:S440, A:D441, A:R442, A:Q443, A:E444, A:Q445, A:I446, A:D447, A:A448, A:L449, A:A450, A:E451, A:L452, A:L453, A:E454, A:S455, A:G456, A:R457, A:G458, A:E459 | 2 |  |
| 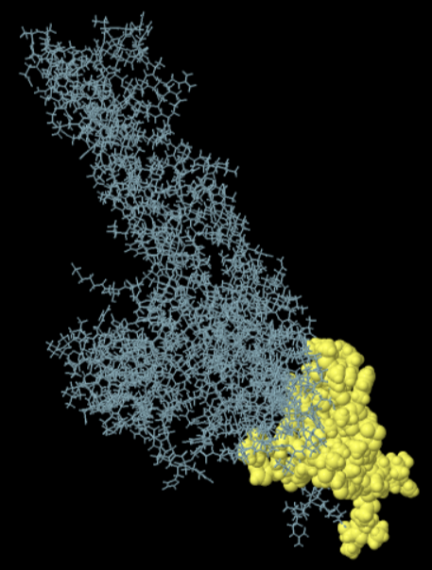 | 0.774 | 45 | A:E270, A:A271, A:L273, A:R274, A:L275, A:A277, A:F278, A:A279, A:R280, A:D281, A:D282, A:A283, A:R284, A:A285, A:A286, A:D287, A:L288, A:P289, A:T351, A:V352, A:G353, A:H354, A:Y355, A:L363, A:K364, A:G365, A:A366, A:R367, A:E368, A:L369, A:T370, A:V371, A:N372, A:D373, A:A374, A:V375, A:L376, A:R377, A:L378, A:L379, A:A380, A:L381, A:R382, A:Q383, A:R384 | 3 |  |
| 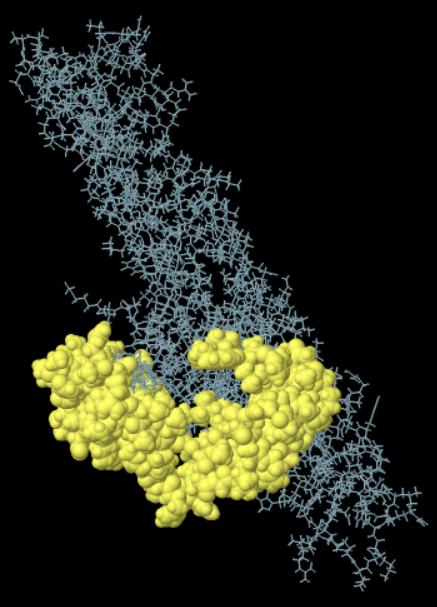 | 0.677 | 94 | A:T15, A:G16, A:S19, A:L20, A:R22, A:T23, A:L24, A:T25, A:R26, A:D27, A:R28, A:G29, A:F30, A:G31, A:E32, A:V33, A:S34, A:H35, A:R36, A:P37, A:S38, A:T39, A:R47, A:L48, A:S49, A:V50, A:D51, A:G52, A:E53, A:E57, A:D66, A:A67, A:I68, A:A69, A:L70, A:L71, A:D72, A:Y73, A:L74, A:D75, A:A76, A:L77, A:E78, A:R79, A:P80, A:G81, A:E82, A:R83, A:L84, A:D85, A:G86, A:P87, A:R89, A:D95, A:S96, A:N97, A:E98, A:A99, A:R100, A:D124, A:A125, A:R126, A:E127, A:P128, A:V129, A:V150, A:L151, A:N152, A:F153, A:V154, A:A155, A:S156, A:P157, A:Q158, A:H159, A:R160, A:E161, A:E162, A:E163, A:W164, A:R165, A:A166, A:A167, A:L168, A:A169, A:R170, A:L171, A:G172, A:L173, A:H174, A:A175, A:L176, A:V177, A:R178 | 4 |  |
| 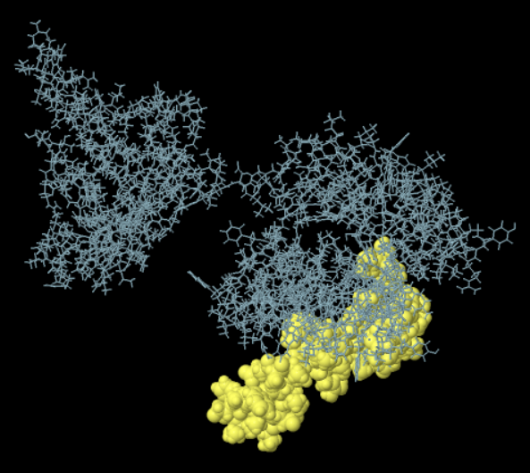 | 0.757 | 50 | A:L1, A:S2, A:H3, A:T4, A:T5, A:H6, A:P7, A:G8, A:L9, A:D10, A:A11, A:L12, A:W13, A:L14, A:T15, A:E16, A:A17, A:V18, A:R19, A:L20, A:R21, A:E22, A:E23, A:Q24, A:A25, A:G26, A:P27, A:L28, A:E29, A:D30, A:S31, A:E32, A:A33, A:V34, A:R35, A:Q36, A:A37, A:L38, A:A39, A:L78, A:A249, A:S250, A:E251, A:A252, A:A253, A:R254, A:H255, A:A256, A:L261, A:V264 | 1 | HP15  HP15  HP15  HP15 |
| 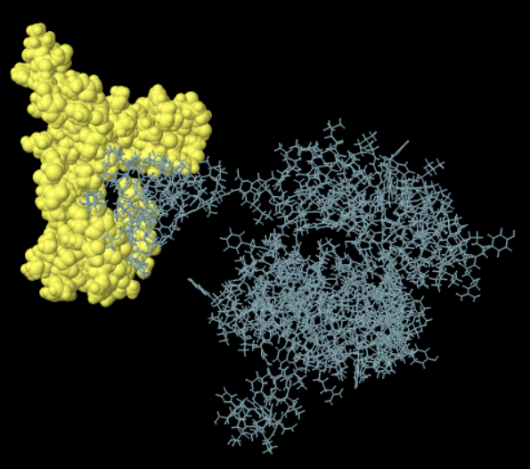 | 0.722 | 112 | A:R313, A:L314, A:G315, A:V316, A:S317, A:D318, A:A319, A:A320, A:P321, A:D322, A:W323, A:L324, A:P325, A:E326, A:P327, A:Q328, A:G329, A:G330, A:Q331, A:S332, A:S333, A:Q334, A:E335, A:A336, A:A337, A:G338, A:A339, A:V340, A:L341, A:V360, A:A361, A:D362, A:A363, A:G364, A:I365, A:L366, A:D367, A:D368, A:G369, A:Q370, A:Q371, A:R372, A:R373, A:R374, A:L375, A:L376, A:E377, A:Q378, A:L379, A:T380, A:R381, A:Y382, A:P383, A:P384, A:A385, A:R386, A:L387, A:A388, A:A390, A:R399, A:A403, A:G406, A:E407, A:L408, A:A409, A:R410, A:C411, A:A412, A:A413, A:S414, A:T415, A:R416, A:I417, A:W418, A:L419, A:L420, A:Q421, A:A422, A:P423, A:P424, A:G425, A:E426, A:A427, A:L428, A:D429, A:S430, A:L433, A:G434, A:W436, A:H437, A:A438, A:A439, A:L440, A:E441, A:R442, A:L443, A:Q444, A:L445, A:P446, A:H447, A:G448, A:E449, A:T450, A:S451, A:P452, A:L453, A:A454, A:W455, A:T458, A:G459, A:H460, A:D461 | 2 |  |
| 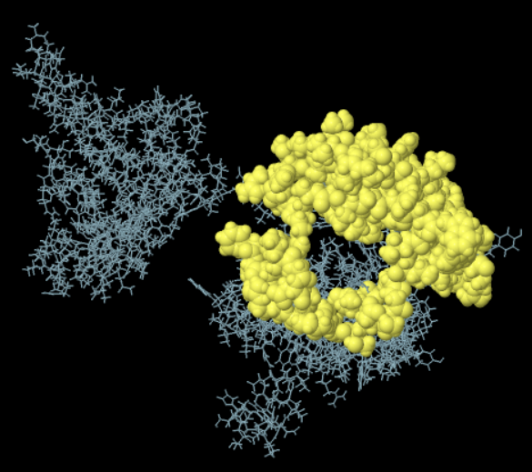 | 0.671 | 85 | A:L62, A:D63, A:G87, A:A91, A:F92, A:A93, A:A94, A:L95, A:G96, A:D97, A:G98, A:Q99, A:R100, A:L139, A:W140, A:L141, A:W142, A:L143, A:S144, A:G145, A:K146, A:L147, A:A148, A:R149, A:D150, A:A151, A:R152, A:A153, A:A154, A:H155, A:L156, A:A157, A:P158, A:A159, A:L160, A:L161, A:V162, A:L163, A:L164, A:G165, A:R166, A:R167, A:R168, A:L169, A:A170, A:R171, A:W172, A:G173, A:L174, A:G175, A:A176, A:L177, A:V178, A:H179, A:G180, A:L181, A:L183, A:L184, A:G185, A:L186, A:L187, A:A191, A:M192, A:L194, A:G195, A:L196, A:L197, A:A198, A:T199, A:R200, A:R201, A:G203, A:F204, A:V205, A:W206, A:E207, A:T208, A:T209, A:I210, A:L211, A:G212, A:S213, A:D214, A:T215, A:F216 | 3 |  |
